# Isolation of Sesquiterpenoids and Steroids from the Soft Coral *Sinularia brassica* and Determination of Their Absolute Configuration

**DOI:** 10.3390/md19090523

**Published:** 2021-09-17

**Authors:** Giang Nam Pham, Da Yeun Kang, Min Ju Kim, Se Jong Han, Jun Hyuck Lee, MinKyun Na

**Affiliations:** 1 College of Pharmacy, Chungnam National University, Daejeon 34134, Korea; PhamGiangNam_Y1@hus.edu.vn (G.N.P.); dykang@o.cnu.ac.kr (D.Y.K.); 2 Division of Life Sciences, Korea Polar Research Institute, Incheon 21990, Korea; mjkim1113@kopri.re.kr (M.J.K.); hansj@kopri.re.kr (S.J.H.); 3 Department of Polar Sciences, University of Science and Technology, Incheon 21990, Korea; junhyucklee@kopri.re.kr; 4 Research Unit of Cryogenic Novel Material, Korea Polar Research Institute, Incheon 21990, Korea

**Keywords:** *Sinularia brassica*, sesquiterpenoids, steroids, antileishmanial activity, antimicrobial activity

## Abstract

Two undescribed rearranged cadinane-type sesquiterpenoids (**1**–**2**), named sinulaketol A-B, together with one new chlorinated steroid (**3**), one new gorgosterol (**4**), one known sesquiterpene (**5**), one known dibromoditerpene (**6**) and two known polyhydroxylated steroids (**7**–**8**) were isolated from the soft coral *Sinularia brassica*. The structures of these compounds were established by extensive spectroscopic analysis, including HRESIMS, 1D, and 2D NMR spectroscopy. Their absolute configurations were also determined by the ECD calculations and DP4+ probability analysis. Antileishmanial activity of compounds **1**–**8** was evaluated in vitro against the amastigote forms of *Leishmania donovani*, in which compounds **3**, **6**, and **7** inhibited the growth of *L. donovani* by 58.7, 74.3, 54.7%, respectively, at a concentration of 50 μM. Antimicrobial effect of the isolated compounds were also evaluated against *Candida albicans*, *Staphylococcus aureus*, and *Escherichia coli.* Compound **6**, a brominated diterpene, exhibited antimicrobial effect against *S*. *aureus*.

## 1. Introduction

**Figure 1:**
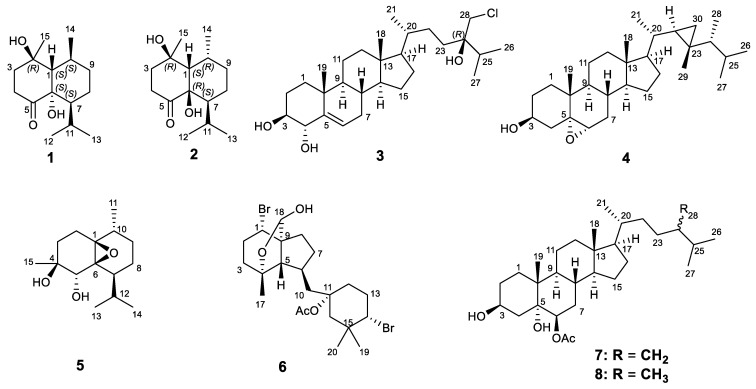
Structures of compounds **1**–**8** isolated from the *Sinularia brassica*.

Soft corals of the genus *Sinularia*, which belong to the order Alcyonacea, are important sources of bioactive natural products and have been the target of study since the middle of twentieth century. Numerous secondary metabolites have been isolated from various *Sinularia* species, particularly sesquiterpenoids, steroids, diterpenoids, and others [1,2,3,4,5]. There is a hypothesis that constituents from soft corals may possess remarkable bioactivities, because their derivatives may act as chemical defense compounds against their predators in various marine ecological environment, to ensure their protection and survival [6]. The object of our study *S. brassica* is an invertebrate which is widely distributed in the Red Sea and Indo-Pacific. To date, only limited steroids, which belong to the withanolide, ergostane, and pregnene type, have been reported in earlier studies on this species [7,8]. Therefore, a further chemical investigation may likely discover more interesting compounds from the soft coral *S. brassica*. Our investigation on the chemical compositions of *S. brassica* had led to the discovery of two undescribed rearranged cadinane-type sesquiterpenoids (**1**–**2**), one new chlorinated steroid (**3**), one new gorgosterol (**4**) (Figure 1), one known sesquiterpene (**5**), one known dibromoditerpene (**6**) and two known polyhydroxylated steroids (**7**–**8**). The structures of these compounds were established by spectrometric and spectroscopic approaches, quantum mechanics-based chemical shifts calculation with support of the DP4+ probability analysis, and comparison with previous literature. The antileishmanial activity of compounds **1–8** was evaluated in vitro against *L. donovani* amastigote forms. In addition, the antimicrobial activities of all the isolates were tested against *Candida albicans*, *Staphylococcus aureus*, and *Escherichia coli*.

## 2. Results

Compound **1** was obtained as colorless gum. The HRESIMS spectrum of **1** exhibited a sodium adduct ion peak at *m/z* 277.1782 [M+Na]^+^ (calcd for C_15_H_26_O_3_Na^+^ 277.1774) (Figure S1), indicating the molecular formula C_15_H_26_O_3_. The ^1^H-NMR spectrum of **1** in CDCl_3_ (Table 1, Figure S3) exhibited signals ascribed to an isopropyl group at δ_H_ 1.76 (m, H-11), 0.95 and 0.72 (each d, *J* = 6.5 Hz, H-12 and H-13), two other methyl groups at δ_H_ 1.43 (s, H-15) and 1.16 (d, *J* = 6.5 Hz, H-14). The evidence of the placement of ketone group was provided by the deshielded chemical shift of the proton resonance of H-4 at δ_H_ 2.75 (ddd, *J* = 15.3, 9.6, 4.7 Hz) and 2.53 (ddd, *J* = 15.3, 8.9, 4.3 Hz) which were ascribable to a neighboring group effect from a ketone group. Fifteen carbon signals were observed in the ^13^C-NMR spectrum of **1**, including one ketone group (δ_C_ 213.5, C-5), two quaternary oxygenated carbons (δ_C_ 72.9, 77.5, C-2 and C-6, respectively), and twelve other resonances which were classified unambiguously by HSQC analysis as four methyls, four methylenes, and four methines (Figure S6). The substituted moieties of **1** were similar to those of cadinanes, but COSY and HMBC analysis suggested a different skeleton. The locations of the methyl group at C-10 and the isopropyl group at C-7 were determined by COSY spectrum with a continuous spin system from H-1-H-10-H-9-H-8-H-7-H-11-H-12, H-14-H-10 and H-11-H-13 (Figure S5); and the strong HMBC correlations of H-14 (δ_H_ 1.16) to C-1 (δ_C_ 51.0), C-10 (δ_C_ 29.1), C-9 (δ_C_ 31.2), and H-12 (δ_H_ 0.95), H-13 (δ_H_ 0.72) to C-7 (δ_C_ 47.9), C-11 (δ_C_ 26.5) (Figure S7). Interestingly, the HMBC spectrum showed cross-peaks between H-15 (δ_H_ 1.43) and C-3 (δ_C_ 39.7), C-2 (δ_C_ 72.9), C-1 (δ_C_ 51.0), revealed the unusual position of methyl group C-15. This fact suggested that the planar architecture of **1** possessed an undescribed rearranged cadinane skeleton.

**Figure 2:**
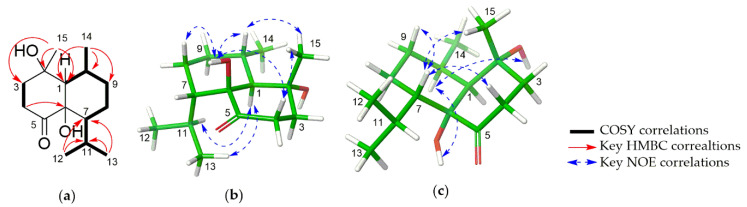
(**a**) Key COSY and HMBC correlations of **1** and **2**. (**b**) and (**c**) MMFF-energy minimized structures and key NOE correlations of **1** and **2**, respectively.

**Figure 3:**
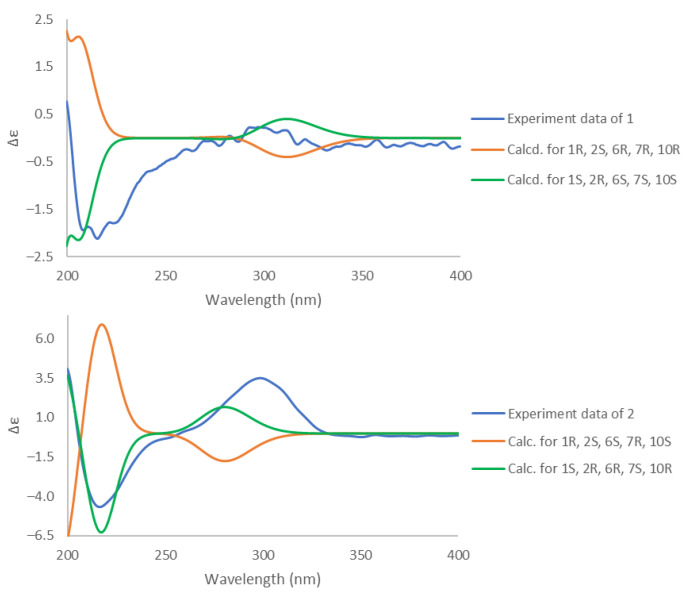
Experimental and calculated ECD spectra of **1** and **2**.

The relative configuration of **1** was determined by the analyses of coupling constants and the NOESY spectrum (Figure 2). The large coupling constant for H-1 at δ_H_ 1.81 (br d, *J* = 11.0 Hz) suggested that H-1 and H-10 should in axial positions, supporting a *trans*-fused ring junction. The NOESY spectrum was measured in DMSO-*d_6_* to observe the correlations of hydroxy groups (Figure S17). In particular, the correlations of H-10 with OH-6 and H-15 in the NOESY spectrum permitted both H-15, H-10 and OH-6 to be oriented on the same side, while the interactions between H-1/H-11, H-1/H-13 indicated that they are on the opposite side of the molecule. The absolute configuration of **1** was identified by ECD calculations performed by the time-dependent density functional theory (TD-DFT) method. The experimental CD spectrum of **1**, which showed good agreement with the calculated ECD spectrum for (1*S*, 2*R*, 6*S*, 7*S*, 10*S*) isomer, and showed mirror-image-like relationship with calculated ECD spectra for (1*R*, 2*S*, 6*R*, 7*R*, 10*R*) isomer, proved the (1*S*, 2*R*, 6*S*, 7*S*, 10*S*) absolute configuration for **1** (Figure 3). Hence, the structure of **1** was completely established and named as sinulaketol A.

Compound **2** shared the same molecular formula as **1**, C_15_H_26_O_3_, which was established from HRESIMS data analysis (Figure S18). The 1D and 2D NMR spectroscopic data of **2** (Table 2, Figures S19–S34) were also similar to those of **1**, suggesting that compound **2** possesses the same chemical backbone as **1** with different stereochemistry. The coupling constant for H-1 at δ_H_ 2.22 (br d, *J* = 4.4 Hz) in the ^1^H-NMR spectroscopic data in CDCl_3_ indicated an axial-equatorial relation between H-1 and H-10 (Table 2, Figure 2 and Figure S26). The NOE correlations (in DMSO-*d*_6_) of OH-6/H-10, OH-6/H-1, H-1/OH-2 and H-10/OH-2 indicated that they should be co-facial, suggesting a *cis*-fused ring (Figure S34). The NOE correlations of H-7/H-4a, H-7/H-15, H-1/H-13 suggested that the isopropyl group is oriented on the same site with H-1. Consequently, the relative configuration of **2** was reaffirmed as a *cis*-fused bicyclic system with 1S*, 2R*, 6R*, 7S*, 10R*. The absolute configuration of **2** was determined by comparison of experimental ECD data with theoretically calculated ECD curves. The pattern of the experimental ECD spectrum of compound **2** was consistent with the calculated ECD curve for (1*S*, 2*R*, 6*R*, 7*S*, 10*R*) isomer (Figure 3). Thus, the absolute configuration of compound **2** was determined as 1*S*, 2*R*, 6*R*, 7*S*, 10*R*, designated as sinulaketol B.

**Figure 4:**
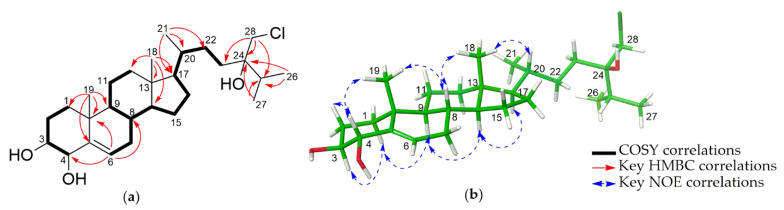
(**a**) Key COSY and HMBC correlations of **3**. (**b**) MMFF-energy minimized structure and key NOE correlations of **3**.

The molecular formula of compound **3** was identified as C_28_H_47_ClO_3_ based on HRESIMS data (Figure S35) at *m/z* 489.3110 [M + Na]^+^ (calcd for C_28_H_47_ClNaO_3_^+^ 489.3106). The ^1^H-NMR spectrum of **3** (Figure S36) showed one olefinic proton at δ_H_ 5.74 (dt, *J* = 2.1, 4.9 Hz, H-6), two oxygenated methines at δ_H_ 3.27 (ddd, *J* = 11.3, 9.4, 4.7 Hz, H-3) and 4.06 (dd, *J* = 9.4, 2.7 Hz, H-4), one chlorinated methylene at δ_H_ 3.68 (d, *J* = 11.2 Hz, H-28a) and 3.56 (d, *J* = 11.2 Hz, H-28b), and 5 methyl groups at δ_H_ 0.69 (s, H_3_-18), 1.02 (s, H_3_-19), 0.93–0,95 (9H, overlap, H_3_-21, H_3_-26, H_3_-27). The ^13^C and HSQC spectroscopic data (Figures S37 and S39) indicated that **3** contained five methyls, ten methylenes, nine methines, and four quaternary carbons, suggesting an ergostane-type steroid. The NMR spectroscopic data of the tetracyclic moiety closely resembled those of 3*β*,4*α*-dihydroxyergosta-5,24(28)-diene [9], indicating that **3** had two hydroxy groups at C-3 and C-4, and a double bond between C-5 and C-6. The proposed structure was confirmed by COSY data (H_2_-1/H_2_-2/H-3/H-4), and HMBC correlations of H_3_-19 to C-5, and H-6 to C-4, C-7, C-8, C-10 (Figure 4a). The relative configuration of **3** was also similar to the reference compound 3*β*,4*α*-dihydroxyergosta-5,24(28)-diene [9], which was supported by the ^1^H-^1^H coupling constants and NOE analysis. The hydroxyl groups at C-3 and C-4 were deduced as *β*- and *α*-forms, respectively, by observation of large diaxial coupling constants (*J* = 9.4 Hz) between H-3 and H-4, and NOE correlations of H-4/H_3_-19, H-3/H-1α, H-1*α*/H-9 (Figure 4b). A chlorinated methylene (C-28) of the side chain in compound **3** was unique, which was confirmed by molecular ion clusters at *m/z* 489.3106 (65%), 491.3093 (35%). The location of the chlorinated methylene and oxygenated quaternary carbon were identified by HMBC analysis, where the correlations of H_2_-28 to C-23, C-24, C25 and H_3_-26, H_3_-27 to C-25, C-24 were observed. The absolute configuration of C-24 was conducted by the gauge-including atomic orbital (GIAO) NMR chemical shifts calculation supported by DP4+ analysis (Tables S3–S5). The calculated ^1^H and ^13^C NMR chemical shifts of the diastereomers 24*S* and 24*R* were compared with those of the experimental NMR data of **3** using the advanced statistics DP4+ (Table 3 and Table S5). The DP4 + analyses demonstrated the probability of 24*R* is 100.0% (Table 3, Table S5). Consequently, the structure of **3** was elucidated as (24*R*)-28-chloroergost-5-ene-3*β*,4*α*,24-triol.

**Figure 5:**
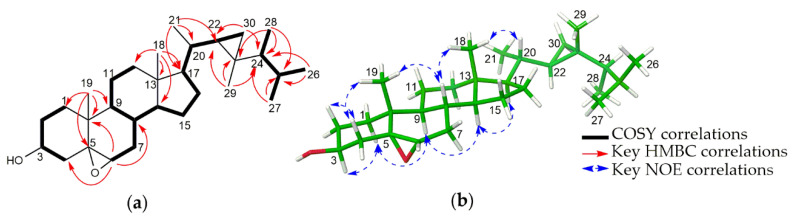
(**a**) Key COSY and HMBC correlations of **4**. (**b**) MMFF-energy minimized structure and key NOE correlations of **4**.

Compound **4** was obtained as amorphous powder. The molecular formula, C_30_H_50_O_2_, was established by quasi-molecular ion peaks at *m/z* 465.3705 [M+Na]^+^ (calcd for C_30_H_50_NaO_2_^+^ 465.3703), 443.3886 [M+H]^+^ (calcd for C_30_H_51_O_2_^+^ 443.3884), 425.3779 [M-H_2_O +H]^+^ (calcd for C_30_H_49_O^+^ 425.3778) in HRESIMS data (Figure S46). The ^1^H NMR spectroscopic data of **4** displayed characteristic proton signals at the very high magnetic field δ_H_-0.14 (dd, *J* = 5.9, 4.3 Hz, H_a_-30), 0.16 (td, *J* = 8.6, 5.7 Hz, H-22), 0.23 (dq, *J* = 9.0, 6.9 Hz, H-24), 0.45 (dd, *J* = 9.1, 4.3 Hz, H_b_-30), indicating a cyclopropyl ring in compound **4** [10]. The stereochemistry of the cyclopropyl ring was identical to that in gorgosterols [11]. The upfield shifted oxygen-bearing methine signal at δ_H_ 2.90 (d, *J* = 4.4 Hz, H-6) and the HMBC correlations from H-6 to C-4/C-7/C-8 indicated the presence of an epoxy bridge between C-5 and C-6 (Figure 5). Comparison of the NMR spectroscopic data of **4** with those of synthetic epoxysitosterols revealed the 5*α*,6*α*-orientation [12]. Therefore, **4** was determined to be 5*α*,6*α*-epoxygorgosterol, whose NMR spectroscopic data are shown in Table 4.

Four known compounds **5**–**8** were identified as nanolobatol A (**5**) [13], pinnaterpene C (**6**) [14], 24-methylenecholestane-3*β*-5α,6*β*-triol-6-monoacetate (**7**) [15,16], and cholestane-3*β*-5α,6*β*-triol-6-monoacetate (**8**) [16], respectively.

All the isolates **1**–**8** were tested in vitro against the amastigote forms of *L. donovani*. Among the compounds **1**–**8**, the new chlorinated steroid (**3**), dibromoditerpene (**6**) and the polyhydroxylated steroid (**7**) inhibited the growth of *L. donovani* by 58.7, 74.3, and 54.7%, respectively, at a concentration of 50 μM, while compounds **3**, **6**, and **7** did not exhibit cytotoxicity against the THP-1 cells (Table 5). In addition, all the isolated compounds were evaluated for their antimicrobial activity against *Candida albicans*, *Staphylococcus aureus*, and *Escherichia coli*. Among the tested metabolites, compound **6** showed antimicrobial activity against *S*. *aureus* (Table 5, Figure S51).

## 3. Discussion

As discussed above, we isolated two rare rearranged cadinene-type sesquiterpenoids (**1**–**2**) and two new steroids (**3**–**4**). Their structures were elucidated by 1D (^1^H, ^13^C) and 2D NMR experiments (HSQC, HMBC, COSY, and NOESY) and confirmed by HRESIMS. Their absolute configurations were comprehensively established by the ECD calculations and NMR chemical shifts calculations supported by DP4+ analysis.

Sesquiterpenoid is one of the significant metabolites of the genus *Sinularia*. During the period of 2013—2021, 35 new sesquiterpenes, including four new carbon skeletons, were isolated from this genus [5]. Although new sesquiterpenoids **1** and **2** belong to cadinane-type skeleton, it is the first report on the cadinane sesquiterpenoids with unprecedented carbon backbone at C-15. They might be derived from the cleavage of C1-C6 bond of ylangene-type sesquiterpenoids, rarely found in the soft corals belonging to the genus *Dendronephthya* and *Lemnalia* [17,18,19].

A new chlorinated steroid (**3**) suppressed the growth of *L. donovani* by 58.7% without cytotoxicity (at 50 μM). Pinnaterpene C (**6**), a dibrominated diterpene, displayed both antileishmanial and antimicrobial activities. This study is the first antileishmanial and antimicrobial investigation for known Pinnaterpene C.

## 4. Materials and Methods

### 4.1. General Experiment Procedures

Vacuum-liquid chromatography (VLC) was conducted on Merck silica gel (70–230 mesh), and Medium-Pressure Liquid Chromatography (MPLC) (Biotage IsoleraTM, Uppsala, Sweden) was performed using Silica gel SNAP cartridge KP-Sil and C_18_ SNAP cartridge KP-C18-HS (Biotage, Charlotte, NC, USA) at a flow rate of 20 mL/min. The sample separation was monitored by thin-layer chromatography (TLC). The TLC was performed on glass pre-coated silica gel 60 F254 plates (Merck, Darmstadt, Germany). Reversed-phase High-performance liquid chromatography (HPLC) was performed on a Gilson HPLC system (Gilson, Inc. Middleton, WI, USA) with a YMC C_18_ Pro Pack 5 µm column (250 × 21.20 mm^2^) (YMC Co., Kyoto, Japan) at a flow rate of 6 mL/min. ^1^H and ^13^C NMR, and 2D (COSY, HSQC, HMBC and NOESY) NMR spectra were recorded on a Bruker AscendTM 600 MHz (Bruker, Billerica, MA, USA). High-resolution Electrospray Ionization mass (HRESIMS) data were obtained utilizing a Synapt G2 Waters mass spectrometer (Waters, Milford, MA, USA). Optical rotations were obtained on a Jasco DIP-1000 automatic digital polarimeter (Tokyo, Japan). Circular dichroism spectrum was recorded on a Chirascan qCD (Applied Photophysics, Leatherhead, Surrey, UK).

### 4.2. Materials and Methods

The soft coral *Sinularia brassica* May 1898 was collected in Van Phong bay, Khanh Hoa province, Vietnam in May 2014 and identified by experts at Institute of Oceanography, Nha Trang, Vietnam. A voucher specimen (E54582) was deposited with the Oceanography Museum, Institute of Oceanography in Nha Trang, Vietnam.

### 4.3. Extraction and Isolation

Freeze-dried bodies of the soft coral *S. brassica* (6.0 kg) were cut into small pieces and then extracted three times with MeOH in ultrasonic condition (1 h) to obtain methanol extract (257.7 g). The methanol extract was suspended in water and then partitioned with *n*-hexane (4 × 2.5 L) to give *n*-hexane soluble fraction (220.8 g) and water layer. The *n*-hexane fraction was subjected to silica gel VLC (20 × 20 cm) and eluted with *n*-hexane/EtOAc (100:1 → 1:1) and EtOAc/MeOH/H_2_O (15:1:0.1 → 2:1:0.3) to yield twelve fractions (Fr 1 ~ Fr 12). Fraction 5 and 6 were combined (56.1 g) and further divided by silica gel VLC (12 × 15 cm, eluting with CH_2_Cl_2_/MeOH (100:1 → 0:1) to give nine fractions (Fr 5.1 ~ Fr 5.9). Fraction 5.4 (5.2 g) was separated by RP-MPLC (column: C_18_ SNAP cartridge KP-C18-HS 120 g) with a stepwise gradient of acetone/H_2_O (60:40, 70:30, 75:25, 80:20, 90:10, 95:5, 100:0, each ~0.3 L) and afforded eight subfractions (Fr 5.4.1 ~ Fr 5.4.8). The subfraction 5.4.2 was further separated by Sephadex LH-20 column chromatography (CC, 1.5 × 60 cm) eluting with CH_2_Cl_2_-MeOH (2:1) to yield two fractions (Fr 5.4.2.1 ~ Fr 5.4.2.2). Compounds **1** (t_R_ = 44.8 min, 2.0 mg) and **2** (t_R_ = 40.2 min, 2.5 mg) were purified from fraction 5.4.2.1 by preparative HPLC [column: YMC C_18_ Pro Pack 5 µm (250 × 21.20 mm)] using a mobile phase of MeOH/H_2_O (60:40, 6 mL/min). Compound **5** (t_R_ = 77.5 min, 4.0 mg) was purified from fraction 5.4.2.2 by preparative HPLC using MeOH/H_2_O (55:45, 6 mL/min). Subfraction 5.4.6 was further divided into eight fractions (Fr 5.4.6.1 ~ Fr 5.4.6.8) by silica gel CC (3.0 × 60 cm) eluting with *n*-hexane/acetone (90:10, 75:25). Fraction 5.4.6.2 was subjected to preparative HPLC using a gradient of MeOH/H_2_O (75:25 for 120 min, 75:25→100:0 for 80 min, 6 mL/min) to yield compound **6** (t_R_ = 142.5 min, 6.5 mg). Compound **4** (t_R_ = 65.3 min, 5.1 mg) was isolated from subfraction 5.4.6.8 by preparative HPLC using MeOH/H_2_O (98:2, 6 mL/min). Fraction 5.6 (2.2 g) was divided into 8 subfractions (Fr 5.6.1 ~ Fr 5.6.8) by NP-MPLC (column: SNAP cartridge KP-Sil 100 g) with a gradient of *n*-hexane/EtOAc (80:20 → 0:100). Subfraction 5.6.7 (120 mg) was separated by Sephadex LH-20 CC with CH_2_Cl_2_-MeOH (4:1), yielding five fractions (Fr 5.6.7.1 ~ Fr 5.6.7.5). Compound **3** (t_R_ = 40.7 min, 6.6 mg) was purified from fraction 5.6.7.2 (15 mg) by preparative HPLC using MeOH/H_2_O (90:10, 6 mL/min). Fraction 5.6.3 and fraction 5.6.4 were combined and separated by preparative HPLC using MeOH/H_2_O (90:10 → 100:0 for 120 min, 6 mL/min) to obtain compounds **7** (t_R_ = 98.0 min, 26.0 mg) and **8** (t_R_ = 109.0 min, 9.9 mg). Full details of the isolation procedures could be found in Supplementary Materials (Figure S51)

Compound **1**: colorless gum; ${\left[a\right]}_{D}^{25}$ +31.1 (*c* 0.1, CHCl_3_); ^1^H and ^13^C NMR data see Table 1; HRESIMS [M+Na]^+^ *m/z* 277.1782 (calculated for C_15_H_26_O_3_Na^+^, 277.1774).

Compound **2**: colorless gum; ${\left[a\right]}_{D}^{25}$ +73.3 (*c* 0.08, CHCl_3_); ^1^H and ^13^C NMR data see Table 2; HRESIMS [M+Na]^+^ *m/z* 277.1782 (calculated for C_15_H_26_O_3_Na^+^, 277.1774).

Compound **3**: white amorphous powder; ${\left[a\right]}_{D}^{25}$ −29.7 (*c* 0.1, CHCl_3_); ^1^H and ^13^C NMR data see Table 3; HRESIMS [M+Na]^+^ *m/z* 489.3110 (calculated for C_28_H_47_ClNaO_3_^+^ 489.3106).

Compound **4**: white amorphous powder; ${\left[a\right]}_{D}^{25}$ −31.6 (*c* 0.1, CHCl_3_); ^1^H and ^13^C NMR data see Table 3; HRESIMS [M+Na]^+^ *m/z* 465.3705 (calculated for C_30_H_50_NaO_2_^+^ 465.3703), [M+H]^+^ *m/z* 443.3886 (calculated for C_30_H_51_O_2_^+^ 443.3884), [M-H_2_O+H]^+^ *m/z* 425.3779 (calculated for C_30_H_49_O^+^ 425.3778).

Nanolobatol A (**5**): colorless gum; ${\left[a\right]}_{D}^{25}$ +27.1 (*c* 0.1, CHCl_3_); ^1^H NMR (600 MHz, CDCl_3_) δ_H_ 3.75 (1H, d, *J* = 9.5 Hz, H-5), 2.39 (1H, d, *J* = 10.7 Hz, 4-OH), 2.27 (1H, pd, *J* = 6.8 3.3 Hz, H-11), 2.04 (1H, ddd, *J* = 15.0, 5.4, 3.2 Hz, H-2a), 1.96 (1H, ddd, *J* = 9.9, 6.3, 3.3 Hz, H-7), 1.81 (1H, ddd, *J* = 15.0, 11.5, 5.5 Hz, H-2b), 1.79 (1H, m, H-10), 1.55 (1H, m, H-8b), 1.51 (1H, m, H-3b), 1.45 (1H, m, H-9b), 1.29 (1H, dddd, *J* = 14.0, 5.5, 3.3, 1.6 Hz, H-3a), 1.26 (3H, s, H-15), 1.14 (1H, m, H-8a), 1.12 (1H, m, H-9a), 1.02 (3H, d, *J* = 6.8 Hz, H-14), 0.98 (3H, d, *J* = 6.9 Hz, H-13), 0.92 (3H, d, *J* = 6.8 Hz, H-12). ^13^C NMR (150 MHz, CDCl_3_) δ_c_ 71.0 (C-1, C-5), 71.0 (C-4), 67.9 (C-6), 42.0 (C-7), 34.6 (C-10), 28.2 (C-11), 27.9 (C-3), 27.8 (C-9), 26.7 (C-15), 24.0 (C-2), 21.9 (C-13), 19.9 (C-8), 17.3 (C-14), 17.2 (C-12); ESI-MS [M+Na]^+^ *m/z* 277.062

Pinnaterpene C (**6**): amorphous powder; ${\left[a\right]}_{D}^{25}$ +8.0 (*c* 0.1, CHCl_3_); ^1^H NMR (600 MHz, CDCl_3_) δ_H_ 5.32 (1H, s, H-18), 4.09 (1H, dd, *J* = 11.8, 6.1 Hz, H-1), 3.93 (1H, dd, *J* = 12.6, 4.0 Hz, H-14), 2.57 (1H, dd, *J* = 14.8, 3.6 Hz, H-16a), 2.41 (1H, m, H-6), 2.40 (1H, m, H-12b), 2.23 (2H, overlap, H-2b, H-10b), 2.20 (1H, m, H-13b), 2.08-2.02 (3H, overlap, H-2a, H-7b, H-13a), 2.02 (3H, s, acetyl), 1.88 (1H, dd, *J* = 14.4, 11.3 Hz, H-10a), 1.62 (1H, m, H-3b), 1.60 (1H, m, H-8b), 1.40 (1H, m, H-3a), 1.35 (1H, m, H-12a), 1.34 (1H, d, *J* = 10.7 Hz, H-5), 1.33 (2H, overlap, H-7a, H-8a), 1.29 (3H, s, H-17), 1.20 (1H, d, *J* = 14.8 Hz, H-16b), 1.11 (3H, s, H-19), 1.03 (3H, s, H-20). ^13^C NMR (150 MHz, CDCl_3_) δ_c_ 170.9 (-COO-), 99.1 (C-18), 83.5 (C-11), 83.2 (C-4), 65.1 (C-14), 63.6 (C-5), 62.4 (C-9), 58.9 (C-1), 46.8 (C-16), 45.3 (C-10), 40.8 (C-3), 37.1 (C-12), 36.7 (C-15), 36.1 (C-6), 32.6 (C-2), 32.6 (C-7), 32.6 (C-20), 32.0 (C-8), 30.4 (C-13), 23.0 (C-22), 22.1 (C-17), 21.8 (C-19); ESI-MS [M+Na]^+^ *m/z* 544.925

24-Methylenecholestane-3*β*-5α,6*β*-triol-6-monoacetate (**7**): white amorphous powder; ${\left[a\right]}_{D}^{25}$ −32.4 (*c* 0.1, CHCl_3_); ^1^H NMR (300 MHz, CDCl_3_) δ_H_ 4.71 (2H, br s, H-6, H-28a), 4.65 (1H, br s, H-28b), 4.08 (1H, m, H-3), 2.22 (1H, m, H-25), 2.08 (1H, m, H-23b), 2.06 (3H, s, acetyl), 1.15 (3H, s, H-19), 1.01 (3H, d, *J* = 6.9 Hz, H-26), 1.01 (3H, d, *J* = 6.9 Hz, H-27), 0.93 (3H, d, *J* = 6.3 Hz, H-21), 0.68 (3H, s, H-18). ^13^C NMR (75 MHz, CDCl_3_) δ_c_ 170.4 (-COO-), 157.0 (C-24), 106.1 (C-28), 76.2 (C-6), 75.5 (C-5), 67.4 (C-3), 56.1 (C-17), 56.0 (C-14), 45.5 (C-9), 42.9 (C-13), 40.6 (C-4), 40.0 (C-12), 38.6 (C-10), 35.9 (C-20), 34.8 (C-22), 33.9 (C-25), 32.1 (C-1), 31.1 (C-23), 30.9 (C-2), 30.7 (C-8), 28.3 (C-16), 24.2 (C-15), 22.1 (C-27), 22.0 (C-26), 21.6 (C-11), 21.2 (C-30), 18.8 (C-21), 16.6 (C-19), 12.3 (C-18)

Cholestane-3*β*-5α,6*β*-triol-6-monoacetate (**8**): white amorphous powder; ${\left[a\right]}_{D}^{25}$ −56.0 (*c* 0.1, CHCl_3_); ^1^H NMR (300 MHz, CDCl_3_) δ_H_ 4.71 (1H, br s, H-6), 4.08 (1H, m, H-3), 2.01 (3H, s, acetyl), 1.14 (3H, s, H-19), 0.90 (3H, s, H-21), 0.85 (3H, d, *J* = 6.9 Hz, H-26), 0.77 (3H, d, *J* = 6.8 Hz, H-26), 0.76 (3H, d, *J* = 6.8 Hz, H-28), 0.68 (3H, s, H-18). ^13^C NMR (75 MHz, CDCl_3_) δ_c_ 170.4 (-COO-), 76.2 (C-6), 75.4 (C-5), 67.4 (C-3), 56.1 (C-17), 56.0 (C-14), 45.5 (C-9), 42.9 (C-13), 40.6 (C-4), 40.0 (C-12), 39.2 (C-24), 38.6 (C-10), 36.4 (C-20), 33.8 (C-22), 32.1 (C-1), 31.6 (C-25), 30.9 (C-2), 30.7 (C-8), 30.7 (C-23), 28.3 (C-16), 24.2 (C-15), 21.6 (C-11), 21.2 (C-30), 20.6 (C-26), 19.0 (C-21), 17.7 (C-27), 16.6 (C-19), 15.6 (C-28), 12.3 (C-18)

### 4.4. Computational Details

The conformational searches of each possible isomer were performed by applying 10,000 steps of the Monte Carlo multiple minimum method with PRCG energy minimization using the Merck Molecular Force Field (MMFF) in gas phase to obtain five conformers for each isomer of **1** and **2**, 18 and 16 conformers for 24*S* and 24*R* isomers of **3** respectively, with a 10 kJ/mol energy window limit. Those occurring conformers were then subjected to geometrical optimization and vibrational frequencies calculation using DFT/B3LYP/6-31G(d,p) level with Gaussian 16 package (Gaussian Inc., Wallingford, CT, USA). All optimized structures have no imaginary frequency, and those of compounds **1** and **2** were then proceeded to ECD calculations at TD-DFT/CAM-B3LYP/6–31+G(d,p) (CPCM, acetonitrile) level. ECD curves were Boltzmann averaged and extracted by SpecDis v.1.7 software with half-band of 0.3 eV.

The optimized conformers of two possible diastereomers of compound **3** (18 and 16 conformers for 24*S* and 24*R* isomers, respectively) were calculated for NMR shielding constants using the gauge—independent atomic orbitals (GIAO) method at the DFT/rmPW1PW91/6-311+G(d,p) (CPCM, chloroform) level. Chemical shift values were calculated by an equation below where $\delta_{calc}^{x}$ is the calculated NMR shift for nucleus *x*, and σ^0^ is the shielding tensor for the proton or carbon nuclei in tetramethylsilane calculated at the same condition.

(1) $$\delta_{calc}^{x}=\frac{\sigma^{0}-\sigma^{x}}{1-\sigma^{0}/10^{6}}$$

The calculated NMR properties were averaged based on the Boltzmann populations of all conformers, and the DP4+ probability analysis was conducted using the Excel sheet provided by Grimblat et al [20,21].

### 4.5. Biological Assays

Antileishmanial activity was evaluated according to the protocol of Institut Pasteur Korea [22]. Antimicrobial activity was determined by dropping compounds on agar plate covered with *S. aureus* KCTC 3881 (bacterium), *E. coli* DH5α (bacterium) and *C. albicans* KCTC 27242 (fungus) (Korean Collection for Type Cultures, Daejeon, Korea). The cell culture was spread on each agar plate up to 10^8^ cells/plate, and then 10 μL of each compound (0.3 and 3 mM) dissolved in 50% DMSO in water were dropped on the plate followed by incubating at 37 °C for 16 h. Antimicrobial activity was determined by the mark of the cell inhibition. Kanamycin and nystatin were used as positive controls against the bacterium and fungus, respectively. The IC_50_ values of the compounds were determined using *S. aureus* KCTC 3881, *E. coli* DH5α and *C. albicans* KCTC 27242 in a 96-well-plate. The cell culture was diluted up to 0.5 McFarland Standard with sterilized media. For *C. albicans*, the culture broth was 100 times more diluted before use. Each well was filled with 95 μL of culture broth. The compounds dissolved in DMSO were added until the final concentrations (1, 2, 5, 10, 20, 50, 100, 200, and 500 μg/mL), and the final volume of each well was 100 μL [23]. The plate was incubated at 37 °C for 16 h. Cell inhibition was measured at 600 nm (for *S. aureus* and *E. coli*) and 530 nm (for *C. albicans*) using Multiskan™ GO Microplate Spectrophotometer (Thermo Scientific, Waltham, MA, USA). The IC_50_ value was calculated using an exponential trend line calculated in Excel (Microsoft, Redmond, WA, USA). Kanamycin and nystatin were used as positive controls against the bacterium and fungus, respectively.

## Figures and Tables

**Table 1:** ^1^H and ^13^C NMR data of compound **1**.

No	δ_H_ * (*J* in Hz)	δ_C_ *	δ_H_^#^ (*J* in Hz)	δ_C_^#^
1	1.81 (br d, 11.0)	51.0	1.55 (br d, 11.0)	50.7
2	-	72.9	-	70.7
3	1.99 (m)	39.7	1.80 (m)	40.1
	1.90 (ddd, 14.0, 9.6, 4.3)		1.74 (m)	
4	2.75 (ddd, 15.3, 9.6, 4.7)	35.4	2.52 (m)	34.6
	2.53 (ddd, 15.3, 8.9, 4.3)		2.35 (ddd, 15.8, 6.3, 4.3)	
5	-	213.5	-	209
6	-	77.5	-	76.2
7	1.72 (m)	47.9	1.77 (m)	44.1
8	1.98 (m)	22.8	1.88 (m)	22.4
	1.62 (ddt, 13.8, 4.4, 2.3)		1.41 (ddd, 13.0, 4.3, 2.0)	
9	1.49 (m)	31.2	1.35 (ddt, 11.9, 4.9, 2.4)	31.2
	1.30 (m)		1.21 (m)	
10	2.12 (m)	29.1	2.04 (m)	28.5
11	1.76 (m)	26.5	1.68 (m)	26.1
12	0.95 (d, 6.3)	22.5	0.90 (d, 6.5)	22.7
13	0.72 (d, 6.5)	23.8	0.66 (d, 6.8)	23.3
14	1.16 (d, 6.5)	22.7	1.06 (d, 6.5)	22.6
15	1.43 (s)	25.5	1.26 (s)	23.1
OH-2	-	-	4.14 (br s)	-
OH-6	-	-	4.79 (br s)	-

* measured in CDCl_3_; ^#^ measured in DMSO-*d_6_*.

**Table 2:** ^1^H and ^13^C NMR data of compound **2**.

No	δ_H_ * (*J* in Hz)	δ_C_ *	δ_H_^#^ (*J* in Hz)	δ_C_^#^
1	2.22 (d, 4.4)	48.6	1.85 (m)	48.5
2	-	71.9	-	70.0
3	2.01 (m)	40.6	1.75 (m)	40.7
	1.90 (m)		1.70 (m)	
4	2.66 (ddd, 18.2, 8.3, 4.5)	35.9	2.52 (m)	35.2
	2.60 (m)		2.33 (ddd, 18.1, 5.7, 3.0)	
5	-	215.3	-	210.3
6	-	79.8	-	78.0
7	1.59 (m)	50.8	1.73 (m)	46.5
8	2.16 (tt, 14.2, 4.2)	19.4	2.06 (m)	18.9
	1.53 (m)		1.36 (m)	
9	1.82 (m)	30.2	1.76 (m)	29.9
	1.36 (dq, 13.6, 3.4)		1.20 (m)	
10	2.44 (m)	28.4	2.39 (m)	27.7
11	1.80 (m)	26.7	1.74 (m)	26.4
12	0.96 (d, 6.6)	22.3	0.90 (d, 6.0)	22.8
13	0.71 (d, 6.7)	24.2	0.64 (d, 6.2)	23.7
14	1.31 (d, 7.3)	18.5	1.25 (d, 7.2)	19.0
15	1.50 (s)	26.1	1.40 (s)	24.9
OH-2	-	-	4.37 (s)	-
OH-6	-	-	4.64 (d, 1.9)	-

* measured in CDCl_3_; ^#^ measured in DMSO-*d*_6_.

**Table 3:** DP4+ probabilities for **3**.

	Isomer 24*S*	Isomer 24*R*
sDP4+ (H data)	0.25%	99.75%
sDP4+ (C data)	12.83%	87.17%
sDP4+ (all data)	0.04%	99.96%
uDP4+ (H data)	0.39%	99.61%
uDP4+ (C data)	2.53%	97.47%
uDP4+ (all data)	0.01%	99.99%
DP4+ (H data)	0.00%	100.00%
DP4+ (C data)	0.38%	99.62%
DP4+ (all data)	0.00%	100.00%

**Table 4:** ^1^H and ^13^C-NMR data of compound **3** and **4** in CDCl_3_.

No	3		4	
	δ_H_ (*J* in Hz)	δ_C_	δ_H_ (*J* in Hz)	δ_C_
1	1.84 (m) 1.13 (m)	36.8	1.69 (m) 1.37 (m)	32.6
2	1.90 (m) 1.60 (m)	28.2	1.92 (m) 1.61 (m)	31.3
3	3.27 (ddd, 11.3, 9.4, 4.7)	76.7	3.91 (tt, 11.3, 4.8)	68.9
4	4.06 (dd, 9.4, 2.7)	75.3	2.07 (dd, 12.7, 11.3) 1.30 (m)	40.0
5	-	142.1	-	65.8
6	5.74 (dt, 2.1, 4.9)	117.9	2.90 (d, 4.4)	59.5
7	2.10 (m) 1.58 (m)	31.6	1.92 (m) 1.49 (dd, 15.6, 9.9)	29.0
8	1.44 (m)	31.7	1.36 (m)	30.1
9	0.99 (m)	50.6	1.25 (m)	42.7
10	-	38.2	-	35.0
11	1.49 (m)	21.0	1.38 (m) 1.25 (m)	20.8
12	1.16 (m) 2.02 (m)	39.8	1.13 (m) 1.97 (m)	39.6
13	-	42.4	-	42.9
14	1.00 (m)	56.8	0.96 (m)	56.9
15	1.10 (m); 1.62 (m)	24.4	1.00 (m); 1.58 (m)	24.4
16	1.27 (m); 1.89 (m)	28.4	1.28 (m); 2.00 (m)	28.2
17	1.14 (m)	55.8	1.20 (m)	57.8
18	0.69 (s)	12.0	0.59 (s)	12.1
19	1.02 (s)	20.4	1.06 (s)	16.1
20	1.40 (m)	36.3	0.98 (m)	35.4
21	0.95 (overlap)	18.9	0.97 (br s)	21.2
22	1.42 (m) 1.07 (m)	29.0	0.16(td, 8.6, 5.7)	32.2
23	1.70 (m); 1.41 (m)	31.1	-	25.9
24	-	75.4	0.23 (dq, 9.0, 6.9)	50.9
25	1.95 (m)	33.2	1.56 (m)	32.2
26	0.93 (overlap)	16.8	0.85 (d, 6.6)	21.7
27	0.94 (overlap)	17.2	0.94 (d, 6.7)	22.3
28	3.56 (d, 11.2) 3.68 (d, 11.2)	51.7	0.93 (d, 6.9)	15.6
29	-	-	0.89 (s)	14.4
30	-	-	−0.14 (dd, 5.9, 4.3) 0.45 (dd, 9.1, 4.3)	21.4

**Table 5:** Antileishmanial and antimicrobial activity of compounds **1**–**8.**

Compound	Antileishmanial		Antimicrobial		
	Inhibition of Parasites (%) ^a^	Cell Viability (%) ^b^	*C. albicans*	*S. aureus*	*E. coli*
			IC_50_ (μg/mL)		
1	−5.3	96.2	NA^c^	NA	NA
2	1.4	96.5	NA	NA	NA
3	58.7	88.8	NA	NA	NA
4	−11.9	97.1	NA	NA	NA
5	−12.3	97.0	NA	NA	NA
6	74.3	106.2	NA	>104	NA
7	54.7	96.1	NA	NA	NA
8	39.0	92.7	NA	NA	NA
kanamycin			NA	13.5	1.50 ± 0.24
nystatin			0.93 ± 0.18	NA	NA

^a^ Inhibition of a growth of *L. donovani* at 50 μM, ^b^ Cell viability of compounds in THP-1 cell at 50 μM, NA: Not active at 500 μg/mL.

## Supplementary Materials

The following are available online at https://www.mdpi.com/article/10.3390/md19090523/s1, Figure S1: HRESIMS of **1**, Figure S2: ^1^H NMR spectrum of **1** in CDCl_3_, Figure S3: ^1^H NMR spectrum of **1** in CDCl_3_ (expanded), Figure S4: 13C NMR spectrum of **1** in CDCl_3_, Figure S5: COSY spectrum of **1** in CDCl_3_, Figure S6: HSQC spectrum of **1** in CDCl_3_, Figure S7: HMBC spectrum of **1** in CDCl_3_, Figure S8: HMBC spectrum of **1** in CDCl_3_ (expanded), Figure S9: ^1^H NMR data of **1** in DMSO-*d_6_*, Figure S10: ^1^H NMR data of **1** in DMSO-*d_6_* (expanded), Figure S11: ^13^C NMR data of **1** in DMSO-*d_6_*, Figure S12: COSY spectrum of **1** in DMSO-*d_6_*, Figure S13: HSQC spectrum of **1** in DMSO-*d_6_*, Figure S14: HMBC spectrum of **1** in DMSO-*d_6_*, Figure S15: HMBC spectrum of **1** in DMSO-*d_6_* expanded, Figure S16: NOESY spectrum of 1 in DMSO-d6, Figure S17: NOESY spectrum of **1** in DMSO-*d_6_* (expanded), Figure S18: HRESIMS of **2**, Figure S19: ^1^H NMR data of **2** in CDCl_3_, Figure S20: ^1^H NMR data of **2** in CDCl_3_ (expanded), Figure S21: ^13^C NMR data of **2** in CDCl_3_, Figure S22: COSY spectrum of **2** in CDCl_3_, Figure S23: HSQC spectrum of **2** in CDCl_3_, Figure S24: HMBC spectrum of **2** in CDCl_3_ (expanded-1), Figure S25: HMBC spectrum of **2** in CDCl_3_ (expanded-2), Figure S26: NOESY spectrum of **2** in CDCl_3_, Figure S27: ^1^H NMR spectrum of **2** in DMSO-*d_6_*, Figure S28: ^1^H NMR spectrum of **2** in DMSO-*d_6_* (expanded), Figure S29: ^13^C NMR spectrum of **2** in DMSO-*d_6_*, Figure S30: COSY spectrum of **2** in DMSO-*d_6_*, Figure S31: HSQC spectrum of **2** in DMSO-*d_6_*, Figure S32: HMBC spectrum of **2** in DMSO-*d_6_* (expanded-1), Figure S33: HMBC spectrum of **2** in DMSO-*d_6_* (expanded-2), Figure S34: NOESY spectrum of **2** in DMSO-*d_6_*, Figure S35: HRESIMS of **3**, Figure S36: ^1^H NMR data of **3** in CDCl_3_, Figure S37: ^13^C NMR data of **3** in CDCl_3_, Figure S38: COSY spectrum of **3** in CDCl_3_, Figure S39: HSQC spectrum of **3** in CDCl_3_, Figure S40: HSQC spectrum of **3** in CDCl_3_ (expanded), Figure S41: HMBC spectrum of **3** in CDCl_3_, Figure S42: HMBC spectrum of **3** in CDCl_3_ (expanded-1), Figure S43: HMBC spectrum of **3** in CDCl_3_ (expanded-2), Figure S44: NOESY spectrum of **3** in CDCl_3_, Figure S45: NOESY spectrum of **3** in CDCl_3_ (expanded), Figure S46: HRESIMS of **4**, Figure S47: ^1^H NMR spectrum of **4** in CDCl_3_, Figure S48: ^13^C NMR spectrum of **4** in CDCl_3_, Figure S49: COSY spectrum of **4** in CDCl_3_, Figure S50: HSQC spectrum of **4** in CDCl_3_, Figure S51: HMBC spectrum of **4** in CDCl_3_, Figure S52: NOESY spectrum of **4** in CDCl_3_, Figure S53: ESI-MS of **5**, Figure S54: ^1^H NMR spectrum of **5** in CDCl_3_, Figure S55: ^13^C NMR spectrum of **5** in CDCl_3_, Figure S56: ESI-MS of **6**, Figure S57: ^1^H NMR spectrum of **6** in CDCl_3_, Figure S58: ^13^C NMR spectrum of **6** in CDCl_3_, Figure S59: ^1^H NMR spectrum of **7** in CDCl_3_, Figure S60: ^13^C NMR spectrum of **7** in CDCl_3_, Figure S61: ^1^H NMR spectrum of **8** in CDCl_3_, Figure S62: ^13^C NMR spectrum of **8** in CDCl_3_, Figure S63: Isolation scheme of **1**–**8**, Figure S64: Antimicrobial activity of **1**–**8**, Table S1: Gibbs Free Energy and Boltzmann Population of **1a** (4*S*, 5*R*, 6*R*, 9*R*, 10*R*) and **1b** (4*R*, 5*S*, 6*S*, 9*S*, 10*S*) for ECD computation, Table S2: Gibbs Free Energy and Boltzmann Population of **2a** (4*S*, 5*R*, 6*S*, 9*R*, 10*S*) and **2b** (4*R*, 5*S*, 6*R*, 9*S*, 10*R*) for ECD computation, Table S3: Gibbs Free Energy and Boltzmann Population of 24*S* isomer of **3** for NMR computation, Table S4: Gibbs Free Energy and Boltzmann Population of 24*R* isomer of **3** for NMR computation, Table S5: Experimental and calculated NMR chemical shift values (ppm) of **3** with diastereomers 24*S* and 24*R*.
