# Supplementary material for: Isolation of Sesquiterpenoids and Steroids from the Soft Coral Sinularia brassica and Determination of Their Absolute Configuration
Source: Mar Drugs. 2021 Sep 17;19(9):523. doi: 10.3390/md19090523 (PMC8466225; doi:10.3390/md19090523)
Supplement: Supplementary file 1 [file marinedrugs-19-00523-s001.zip › marinedrugs-1367063_SI.pdf]

Supplementary materials for

Isolation of Sesquiterpenoids and Steroids from the Soft Coral *Sinularia brassica* and Determination of their Absolute Configuration

Giang Nam Pham[1], Da Yeun Kang[1], Min Ju Kim[2], Se Jong Han[2,3], Jun Hyuck Lee[3,4] and MinKyun Na[1,*]

[1] College of Pharmacy, Chungnam National University, Daejeon 34134, Republic of Korea; nampg@cnu.ac.kr (P.G.N); dykang@o.cnu.ac.kr (D.Y.K.)
[2] Division of Life Sciences, Korea Polar Research Institute, Incheon 21990, Republic of Korea; mjkim1113@kopri.re.kr (M.J.K.); hansj@kopri.re.kr (S.J.H.)
[3] Department of Polar Sciences, University of Science and Technology, Incheon 21990, Republic of Korea
[4] Research Unit of Cryogenic Novel Material, Korea Polar Research Institute, Incheon, 21990, Republic of Korea; junhyucklee@kopri.re.kr (J.H.L.)

* Correspondence: mkna@cnu.ac.kr; Tel.: +82-42-823-6566 (M.N.)

Contents

Figure S1. HRESIMS of **1** 4
Figure S2. ^{1}H NMR spectrum of **1** in $CDCl_3$ 5
Figure S3. ^{1}H NMR spectrum of **1** in $CDCl_3$ (expanded) 6
Figure S4. ^{13}C NMR spectrum of **1** in $CDCl_3$ 7
Figure S5. COSY spectrum of **1** in $CDCl_3$ 8
Figure S6. HSQC spectrum of **1** in $CDCl_3$ 9
Figure S7. HMBC spectrum of 1 in $CDCl_3$ 10
Figure S8. HMBC spectrum of **1** in $CDCl_3$ (expanded) 11
Figure S9. 1H NMR data of **1** in DMSO-d_6 12
Figure S10. ^{1}H NMR data of **1** in DMSO-d_6 (expanded) 13
Figure S11. ^{13}C NMR data of **1** in DMSO-d_6 14
Figure S12. COSY spectrum of **1** in DMSO-d_6 15
Figure S13. HSQC spectrum of **1** in DMSO-d_6 16
Figure S14. HMBC spectrum of **1** in DMSO-d_6 17
Figure S15. HMBC spectrum of **1** in DMSO-d_6 (expanded) 18
Figure S17. NOESY spectrum of **1** in DMSO-d_6 (expanded) 20
Figure S18. HRESIMS of **2** 21

Figure S19. ^{1}H NMR data of **2** in $CDCl_3$ 22
Figure S20. ^{1}H NMR data of **2** in $CDCl_3$ (expanded) 23
Figure S21. ^{13}C NMR data of **2** in $CDCl_3$ 24
Figure S22. COSY spectrum of **2** in $CDCl_3$ 25
Figure S23. HSQC spectrm of **2** in $CDCl_3$ 26
Figure S24. HMBC spectrum of **2** in $CDCl_3$ (expanded - 1) 27
Figure S25. HMBC spectrum of **2** in $CDCl_3$ (expanded - 2) 28
Figure S26. NOESY spectrum of **2** in $CDCl_3$ 29
Figure S27. ^{1}H NMR spectrum of **2** in DMSO-d_6 30
Figure S28. ^{1}H NMR spectrum of **2** in DMSO-d_6 (expanded) 31
Figure S29. ^{13}C NMR spectrum of **2** in DMSO-d_6 32
Figure S30. COSY spectrum of **2** in DMSO-d_6 33
Figure S31. HSQC spectrum of **2** in DMSO-d_6 34
Figure S32. HMBC spectrum of **2** in DMSO-d_6 (expanded – 1) 35
Figure S33. HMBC spectrum of **2** in DMSO-d_6 (expanded - 2) 36
Figure S34. NOESY spectrum of **2** in DMSO-d_6 37
Figure S35. HRESIMS of **3** 38
Figure S36. ^{1}H NMR data of **3** in $CDCl_3$ 39
Figure S37. ^{13}C NMR data of **3** in $CDCl_3$ 40
Figure S38. COSY spectrum of **3** in $CDCl_3$ 41
Figure S39. HSQC spectrum of **3** in $CDCl_3$ 42
Figure S40. HSQC spectrum of **3** in $CDCl_3$ (expanded) 43
Figure S41. HMBC spectrum of **3** in $CDCl_3$ 44
Figure S42. HMBC spectrum of **3** in $CDCl_3$ (expanded - 1) 45
Figure S43. HMBC spectrum of **3** in $CDCl_3$ (expanded- 2) 46
Figure S44. NOESY spectrum of **3** in $CDCl_3$ 47
Figure S45. NOESY spectrum of **3** in $CDCl_3$ (expanded) 48
Figure S46. HRESIMS of **4** 49
Figure S47. ^{1}H NMR spectrum of **4** in $CDCl_3$ 50
Figure S48. ^{13}C NMR spectrum of **4** in $CDCl_3$ 51

Figure S49. COSY spectrum of **4** in $CDCl_3$ 52
Figure S50. HSQC spectrum of **4** in $CDCl_3$ 53
Figure S51. HMBC spectrum of **4** in $CDCl_3$ 54
Figure S52. NOESY spectrum of **4** in $CDCl_3$ 55
Figure S53. ESI-MS of **5** 56
Figure S54. ^1H NMR spectrum of **5** in $CDCl_3$ 57
Figure S55. ^{13}C NMR spectrum of **5** in $CDCl_3$ 58
Figure S56. ESI-MS of **6** 59
Figure S57. ^1H NMR spectrum of **6** in $CDCl_3$ 60
Figure S58. ^{13}C NMR spectrum of **6** in $CDCl_3$ 61
Figure S59. ^1H NMR spectrum of **7** in $CDCl_3$ 62
Figure S60. ^{13}C NMR spectrum of **7** in $CDCl_3$ 63
Figure S61. ^1H NMR spectrum of **8** in $CDCl_3$ 64
Figure S62. ^{13}C NMR spectrum of **8** in $CDCl_3$ 65
Figure S63. Isolation scheme of **1** – **8** 66
Figure S64. Antimicrobial activity of **1** – **8** 67
Table S1. Gibbs Free Energy and Boltzmann Population of **1a** (1*R*, 2*S*, 6*R*, 7*R*, 10*R*) and **1b** (1*S*, 2*R*, 6*S*, 7*S*, 10*S*) for ECD computation 68
Table S2. Gibbs Free Energy and Boltzmann Population of **2a** (1*R*, 2*S*, 6*S*, 7*R*, 10*S*) and **2b** (1*S*, 2*R*, 6*R*, 7*S*, 10*R*) for ECD computation 69
Table S3. Gibbs Free Energy and Boltzmann Population of 24*S* isomer of **3** for NMR computation 70
Table S4. Gibbs Free Energy and Boltzmann Population of 24*R* isomer of **3** for NMR computation 71
Table S5. Experimental and calculated NMR chemical shift values (ppm) of compound **3** with diastereomers 24*S* and 24*R* 72

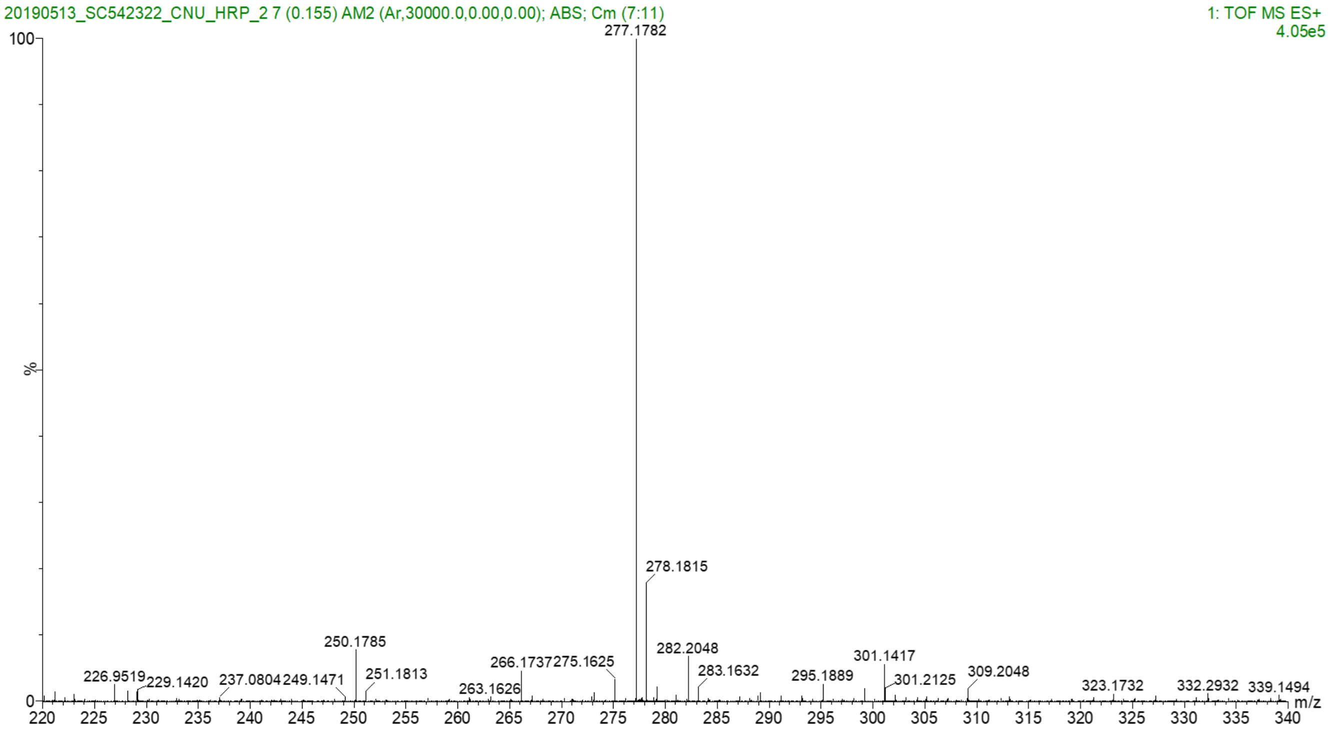

Figure S1. HRESIMS of **1**

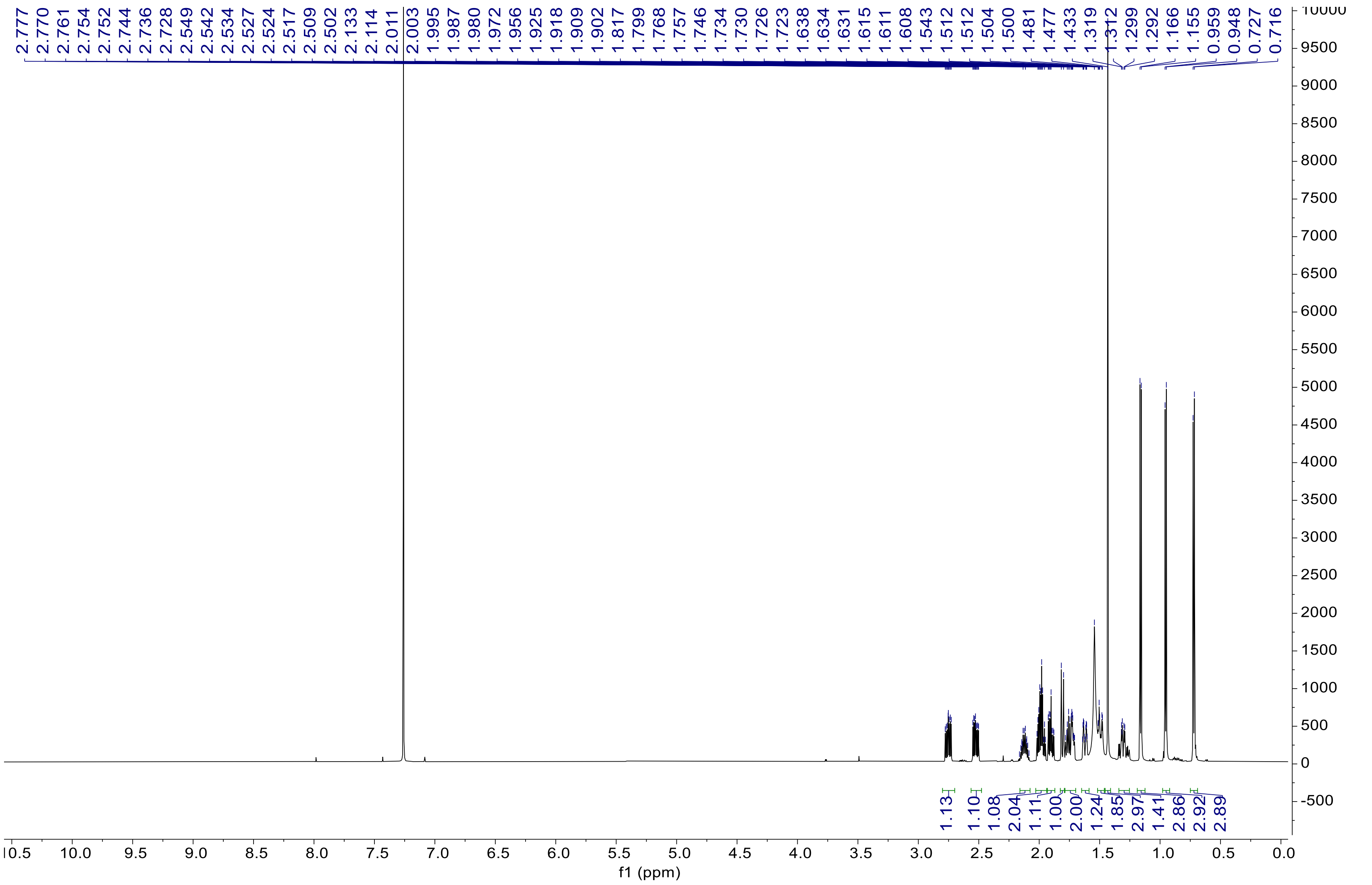

Figure S2. ^{1}H NMR spectrum of **1** in $CDCl_3$

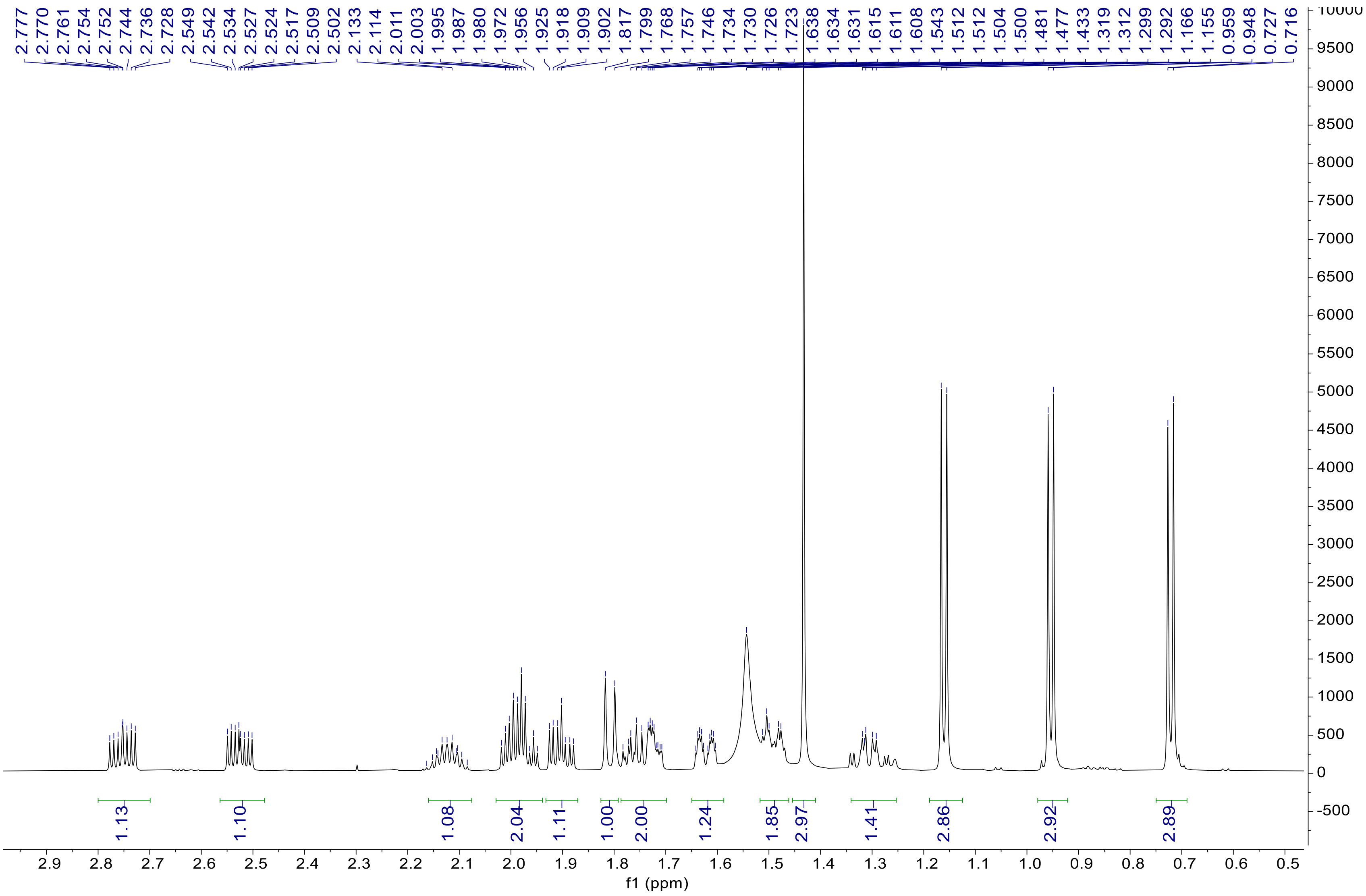

Figure S3. ^{1}H NMR spectrum of **1** in $CDCl_3$ (expanded)

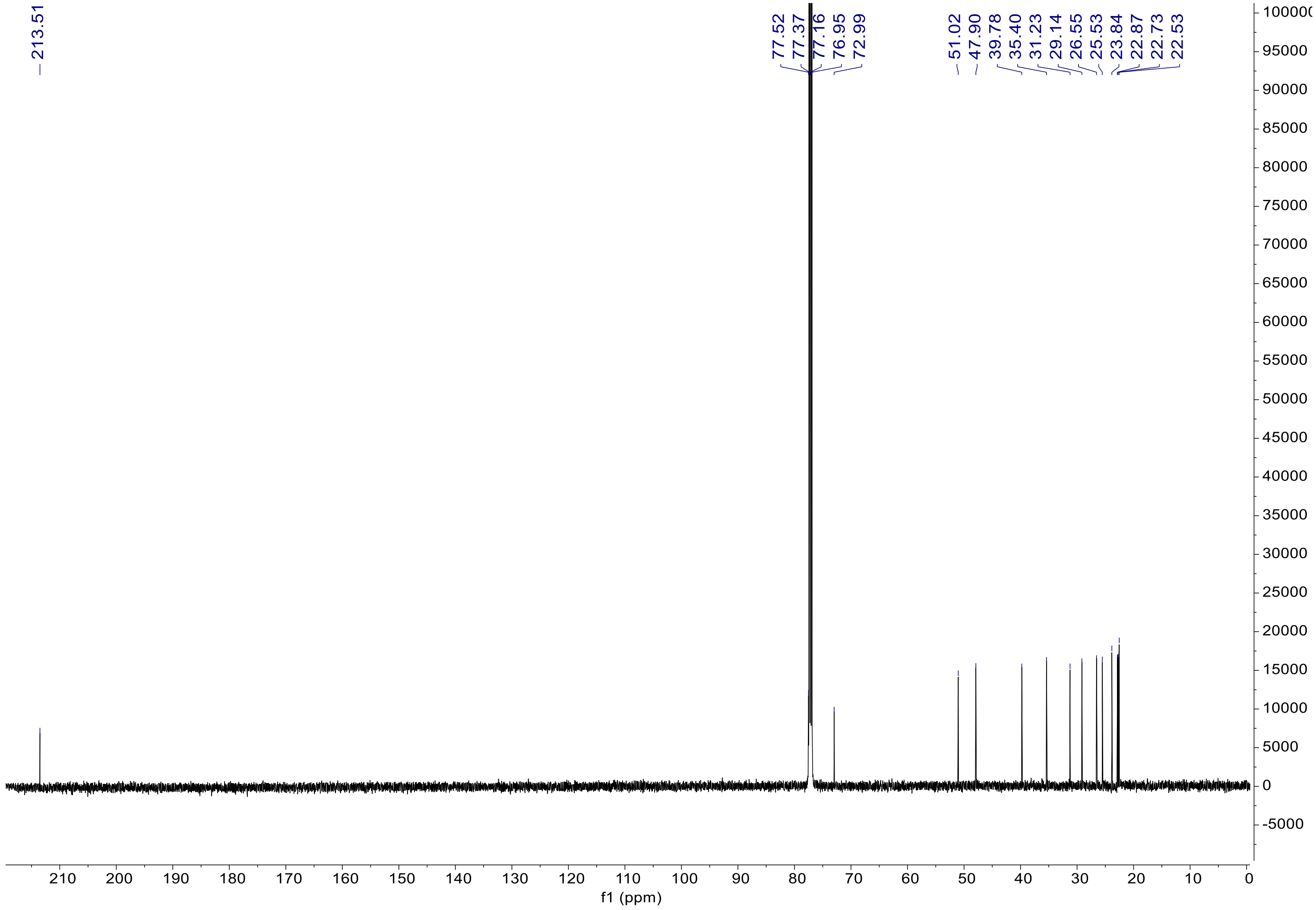

Figure S4. ^{13}C NMR spectrum of **1** in $CDCl_3$

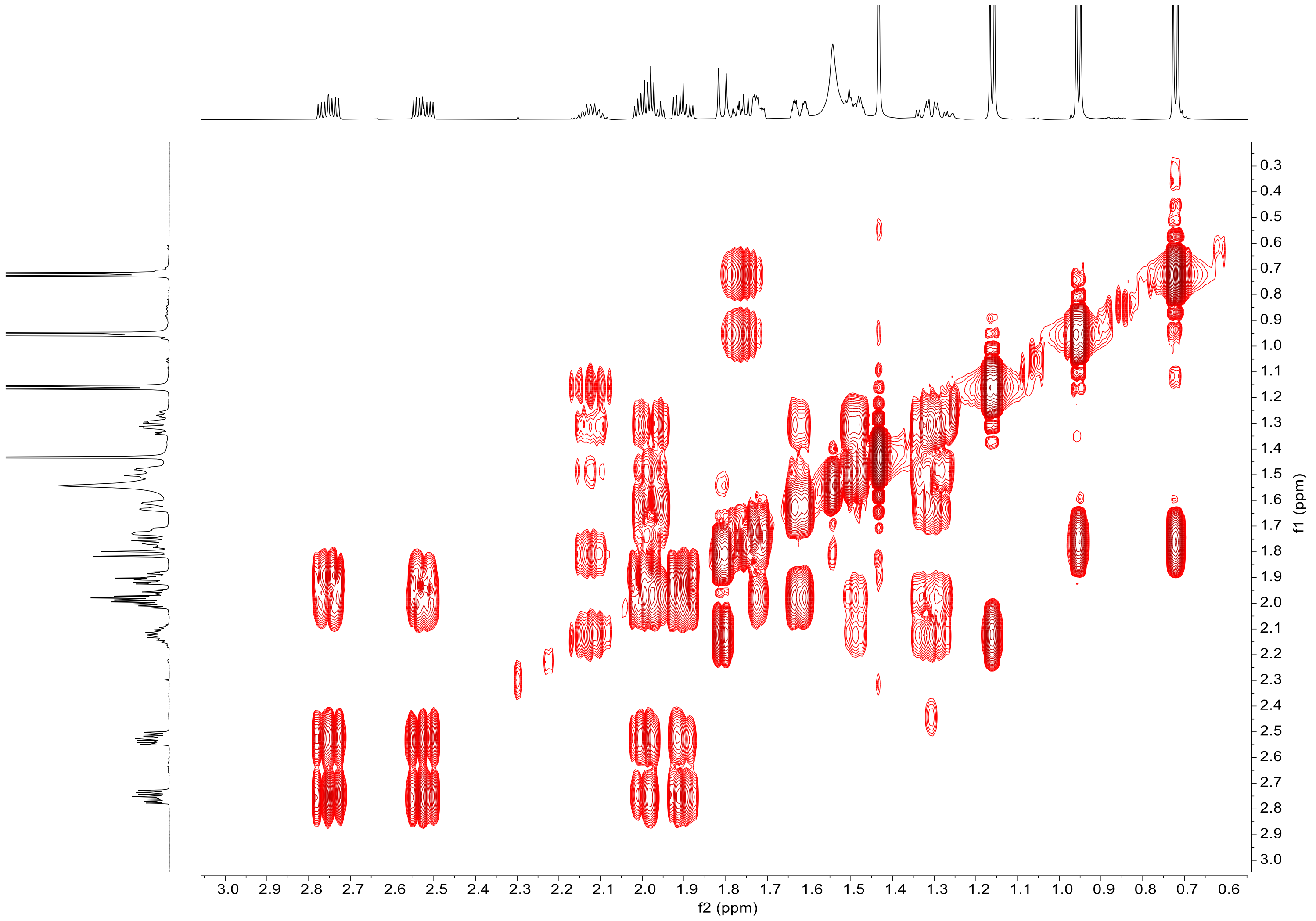

Figure S5. COSY spectrum of **1** in $CDCl_3$

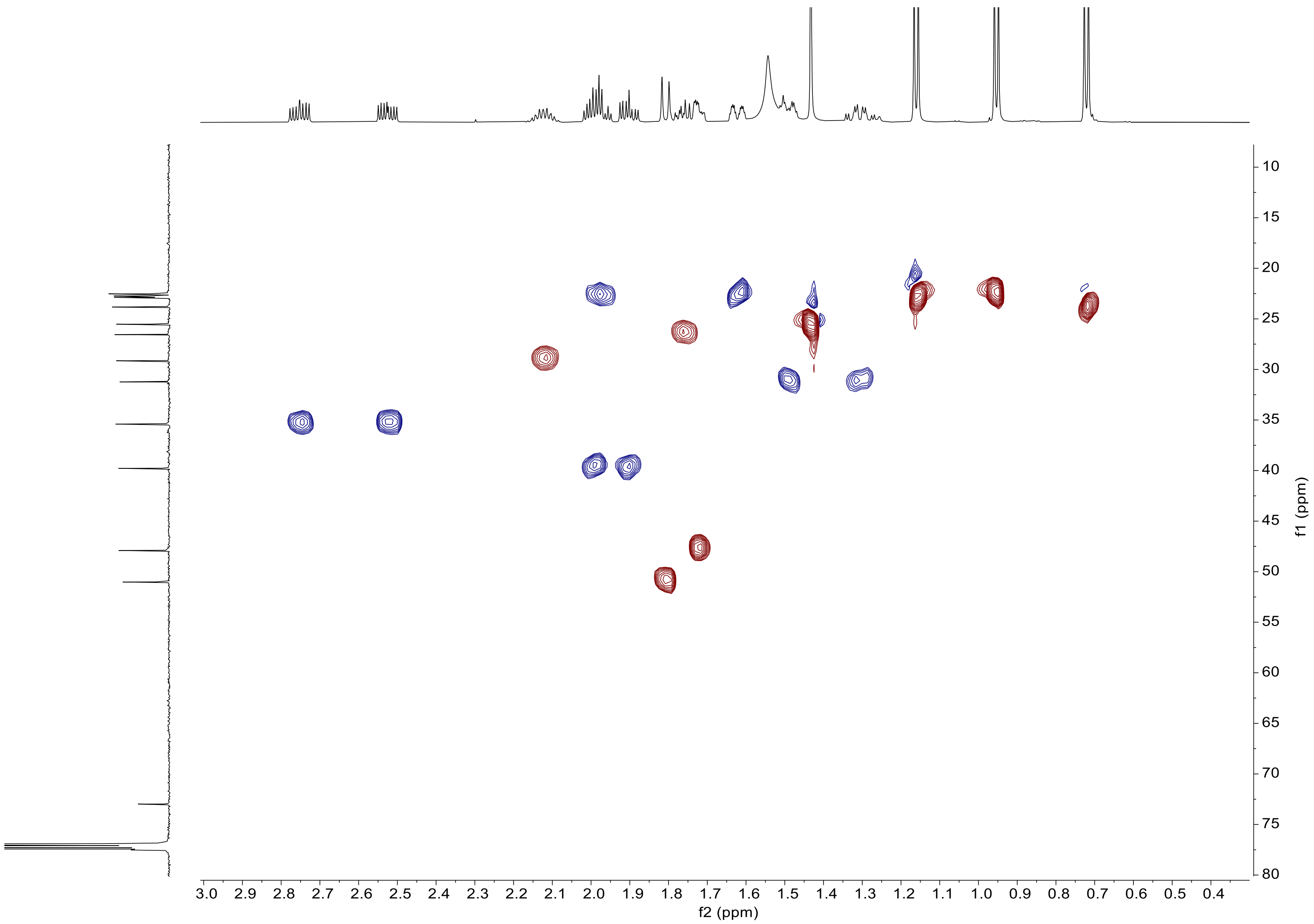

Figure S6. HSQC spectrum of **1** in $CDCl_3$

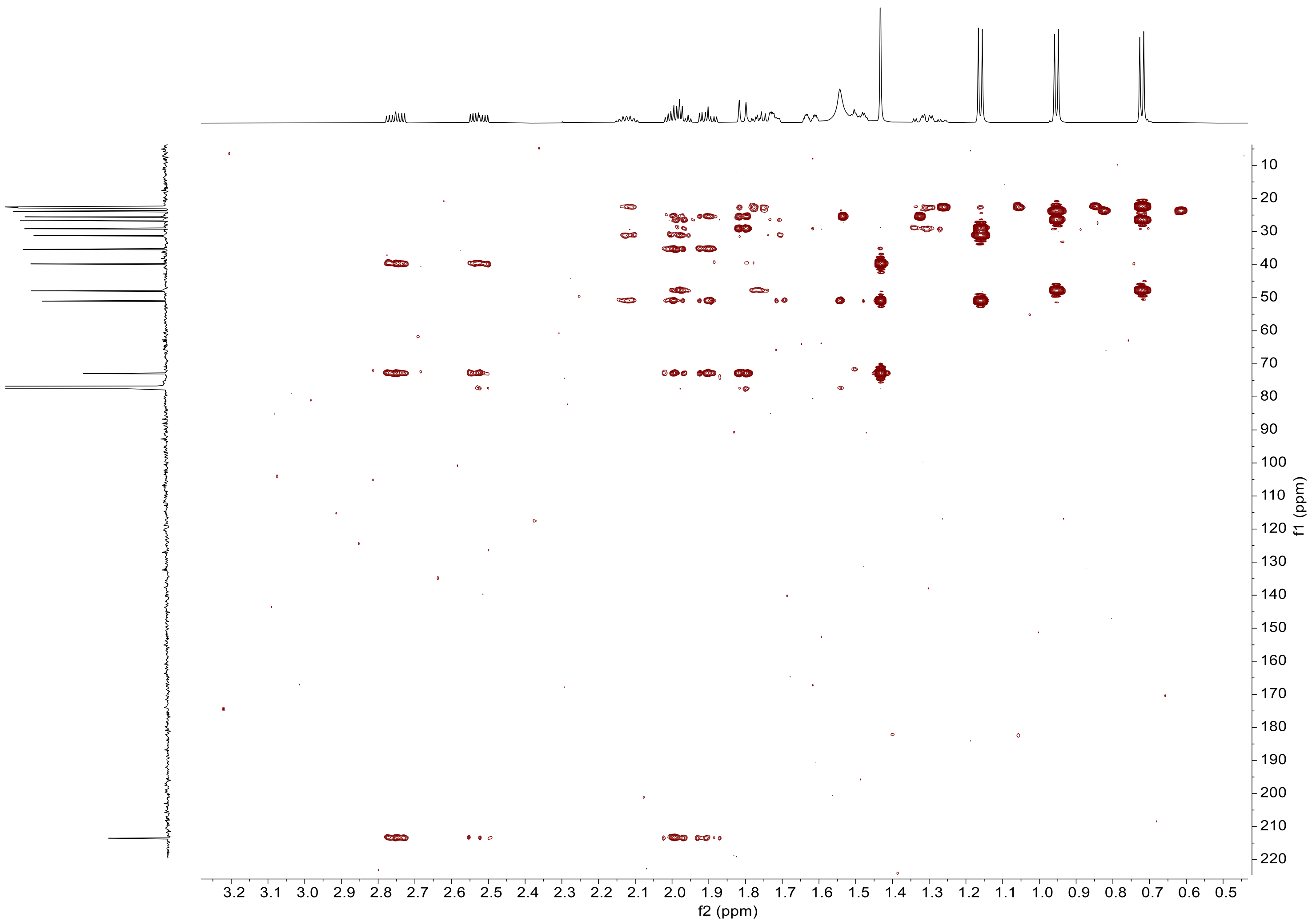

Figure S7. HMBC spectrum of 1 in $CDCl_3$

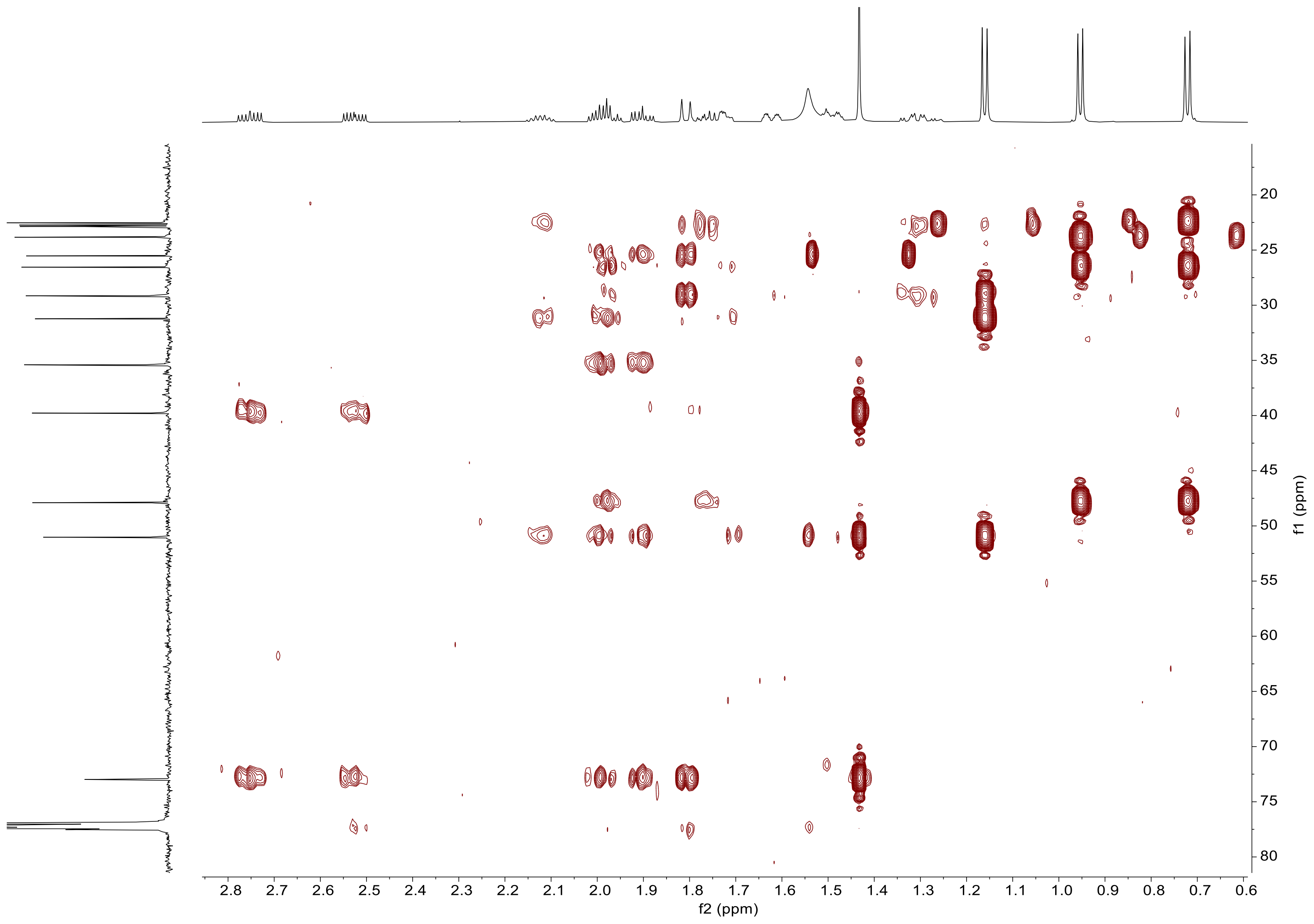

Figure S8. HMBC spectrum of **1** in $CDCl_3$ (expanded)

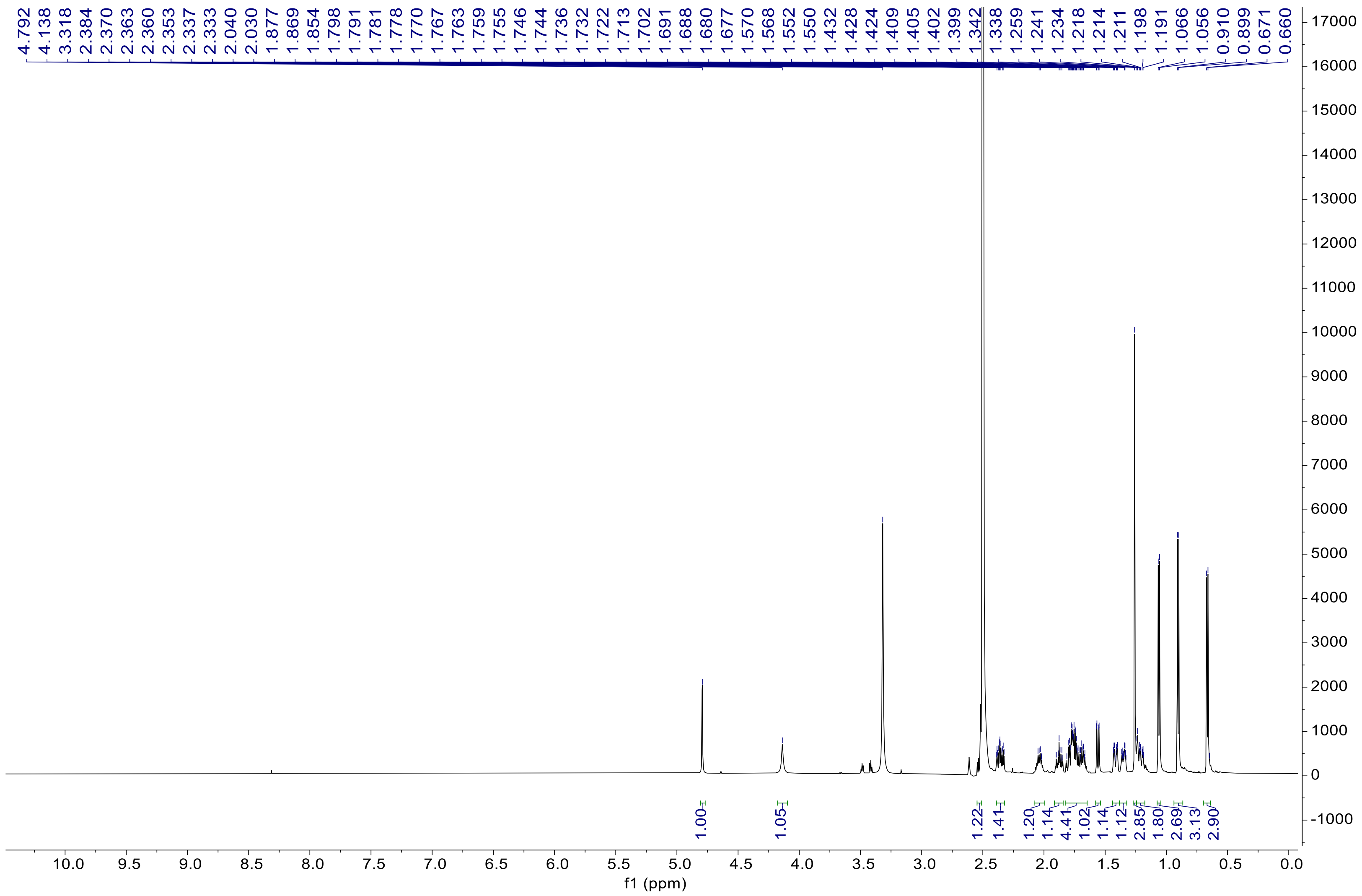

Figure S9. 1H NMR data of **1** in DMSO-*d*₆

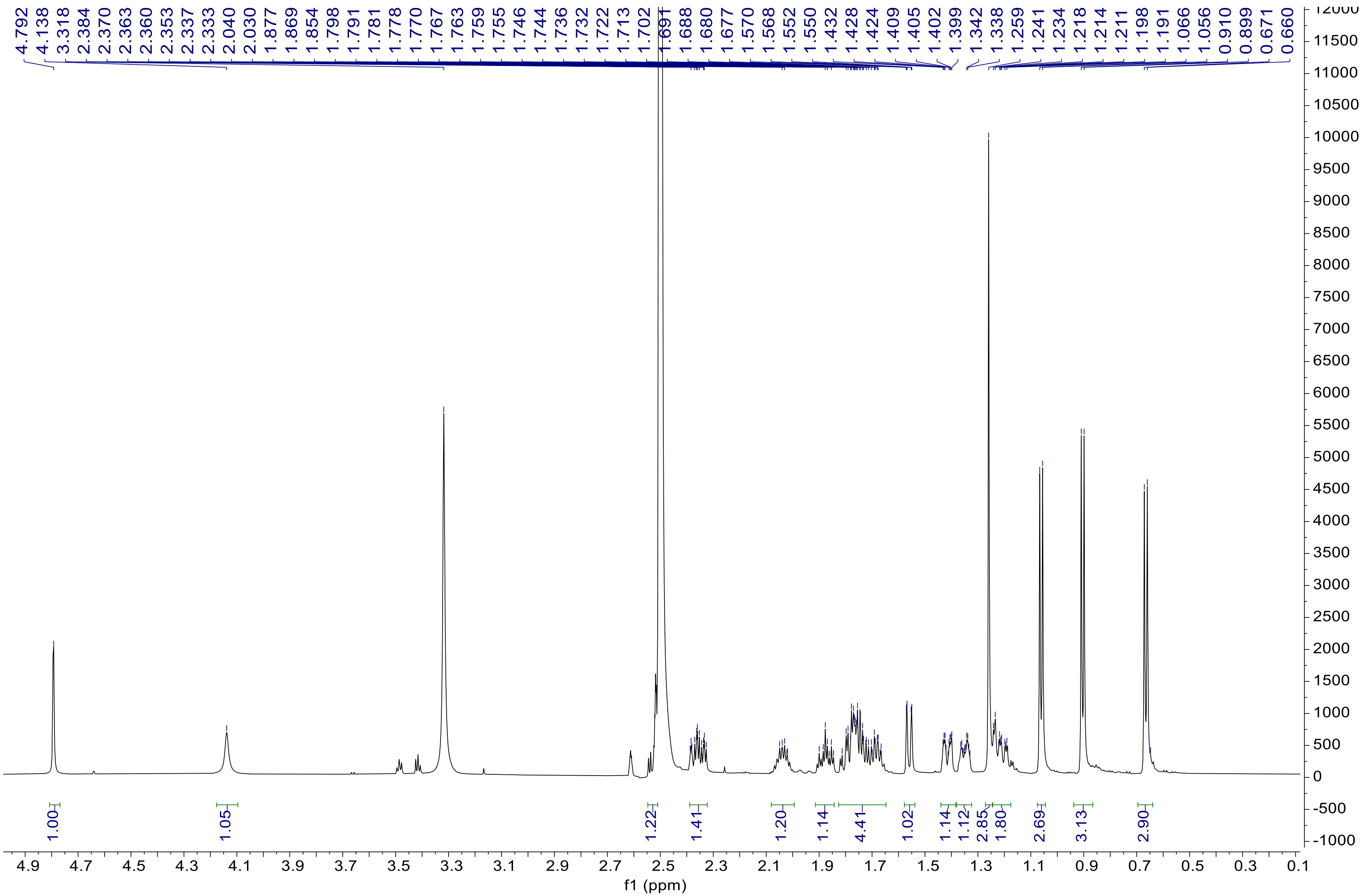

Figure S10. ^{1}H NMR data of **1** in DMSO-d_6 (expanded)

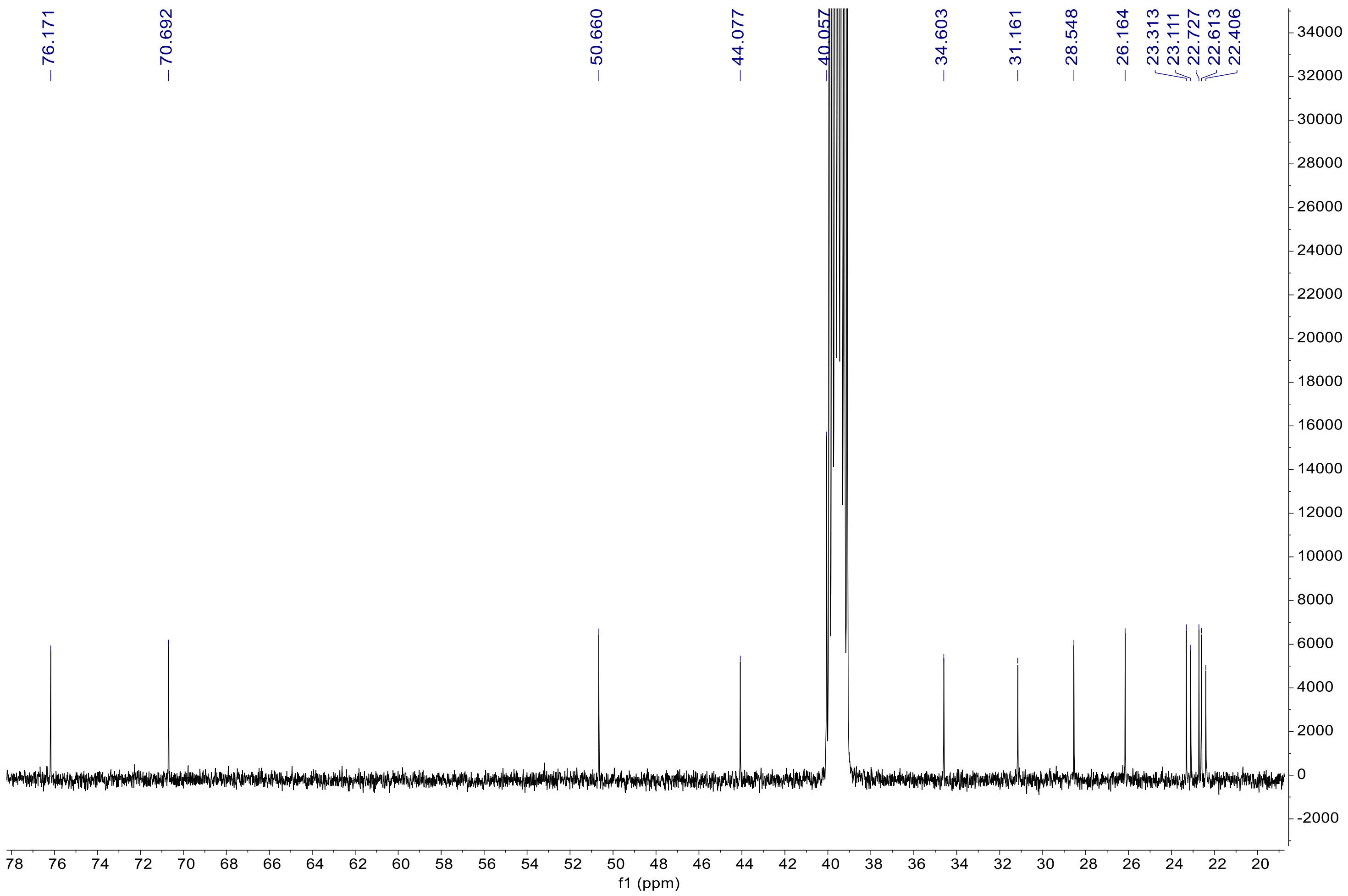

Figure S11. ^{13}C NMR data of **1** in DMSO-d_6

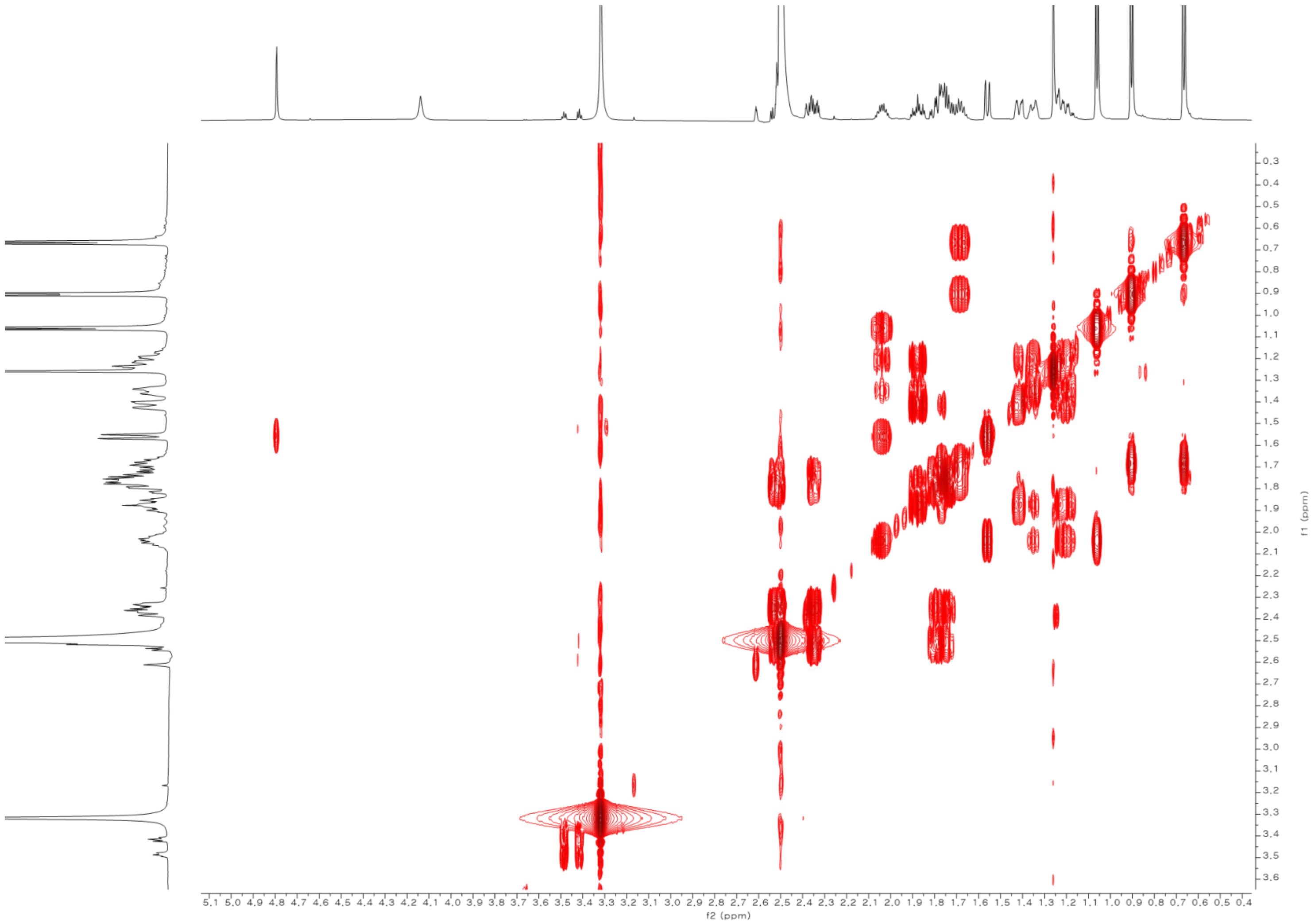

Figure S12. COSY spectrum of **1** in DMSO-d_6

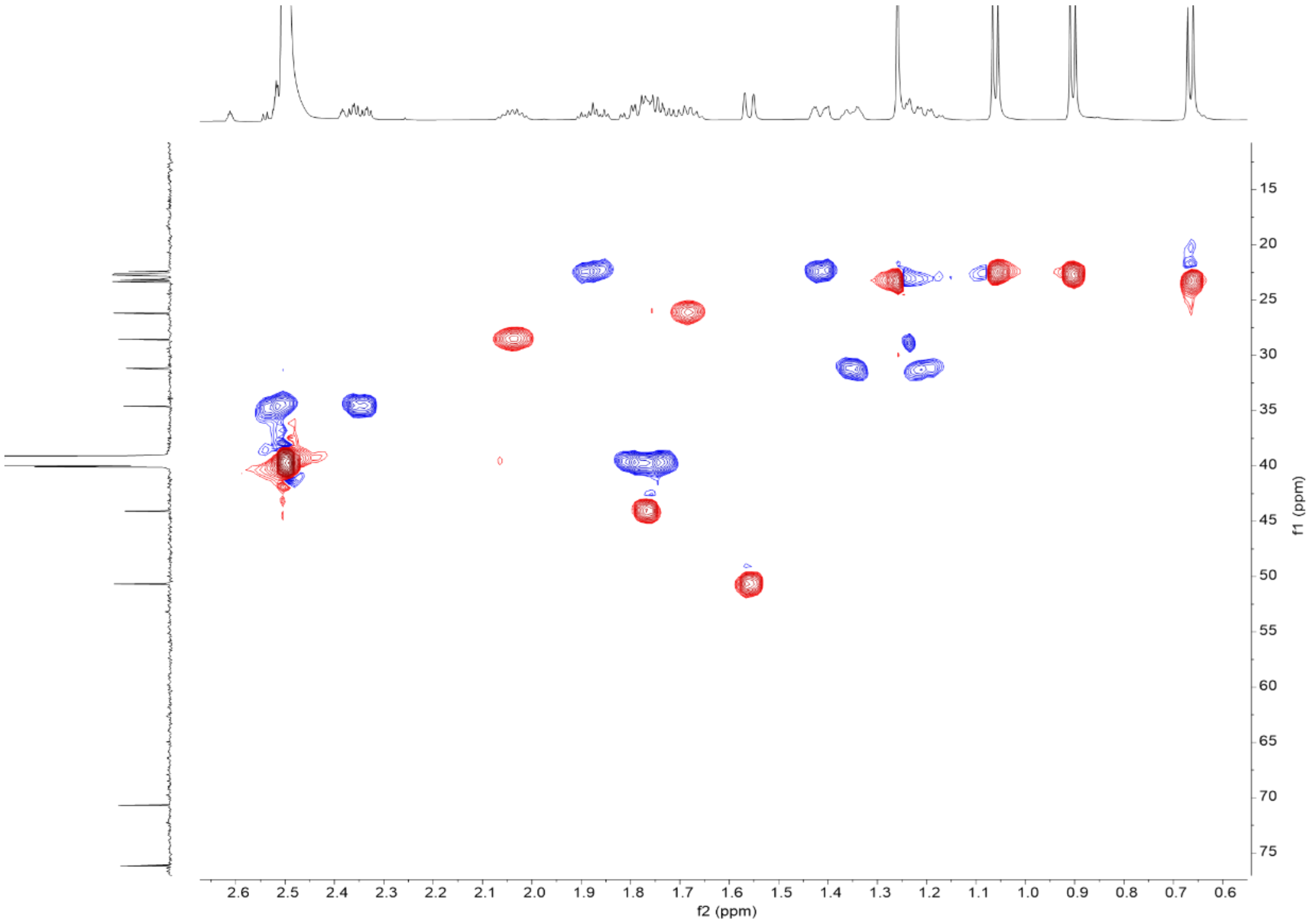

Figure S13. HSQC spectrum of **1** in DMSO-d_6

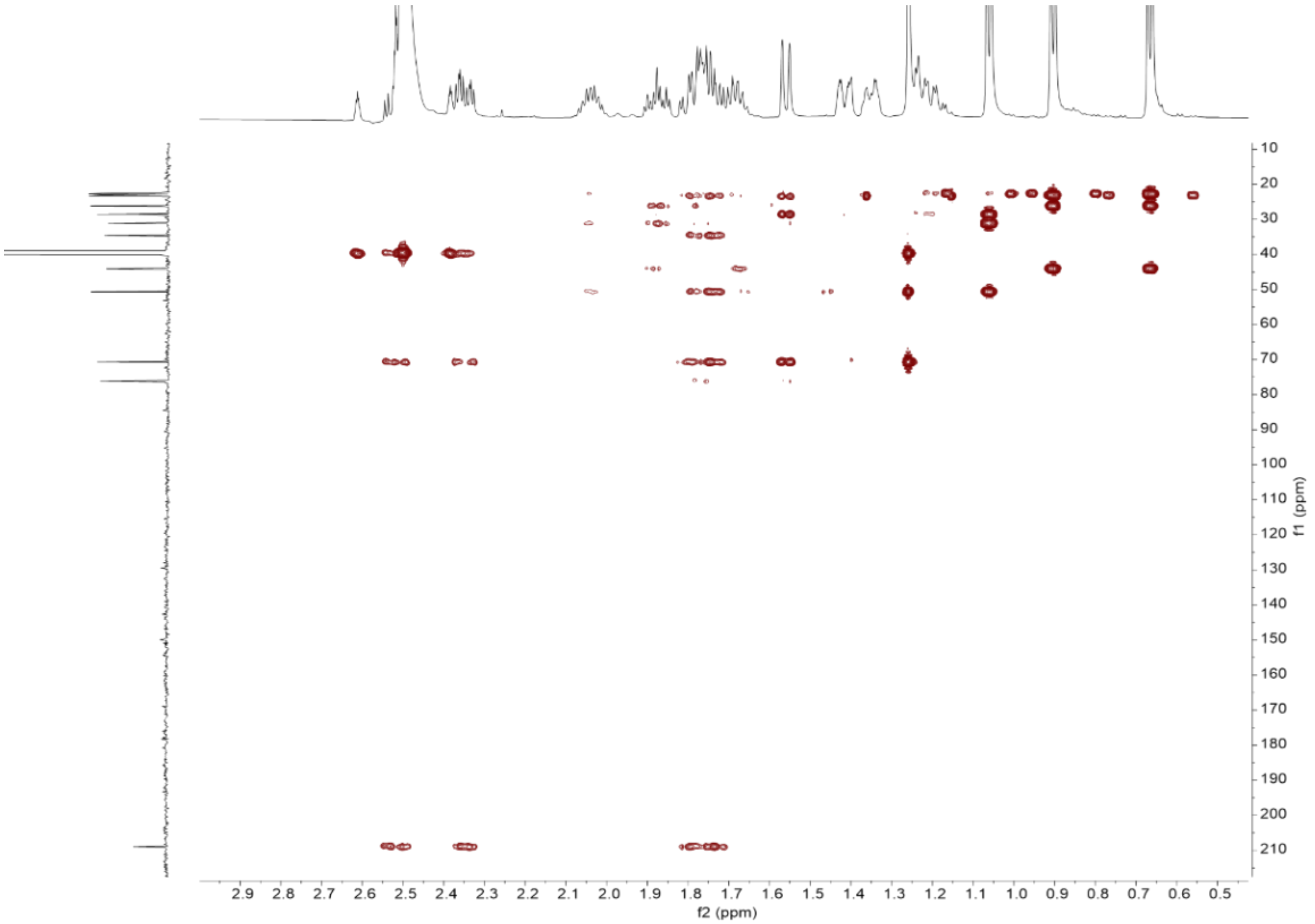

Figure S14. HMBC spectrum of **1** in DMSO-d_6

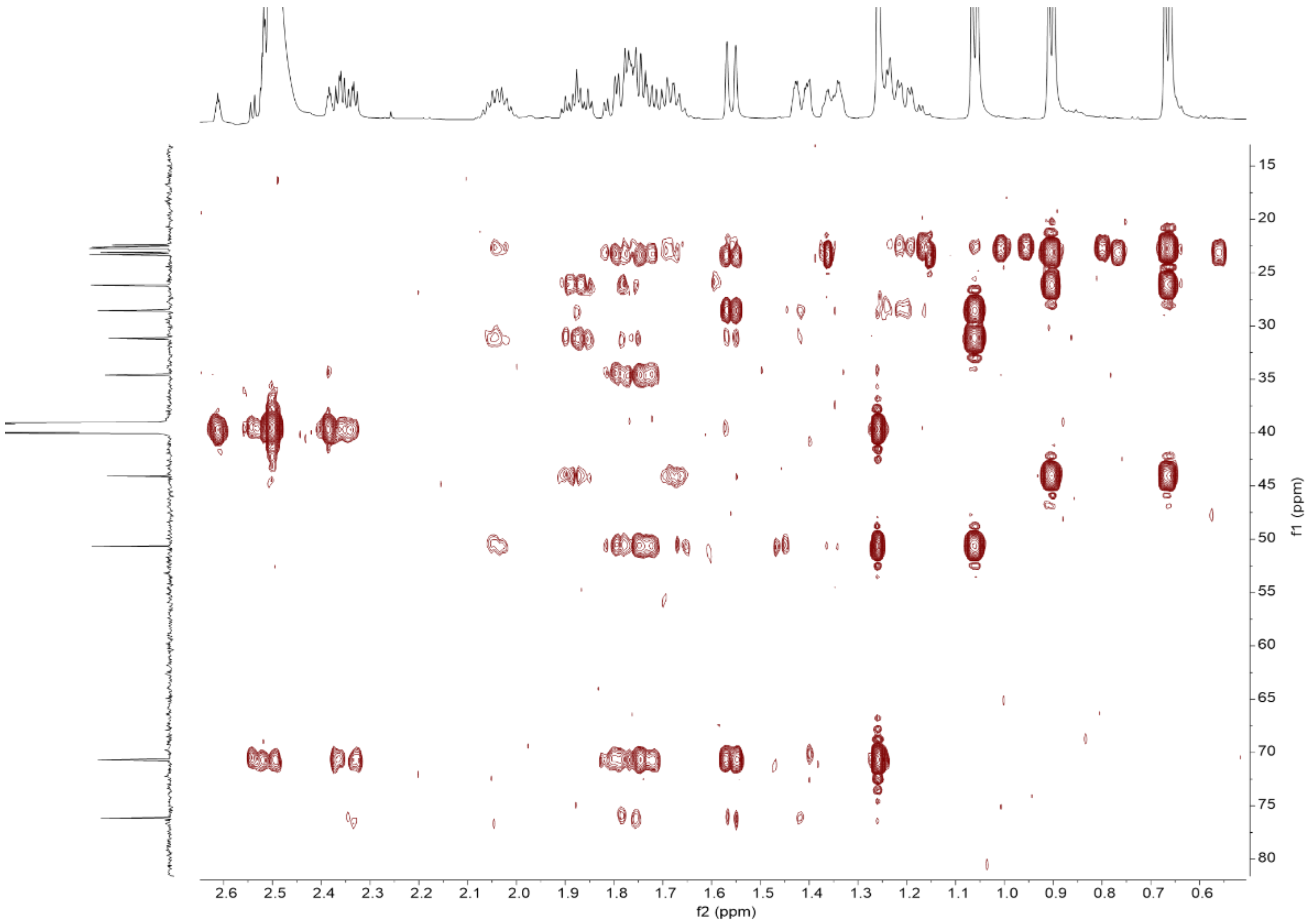

Figure S15. HMBC spectrum of **1** in DMSO-d_6 (expanded)

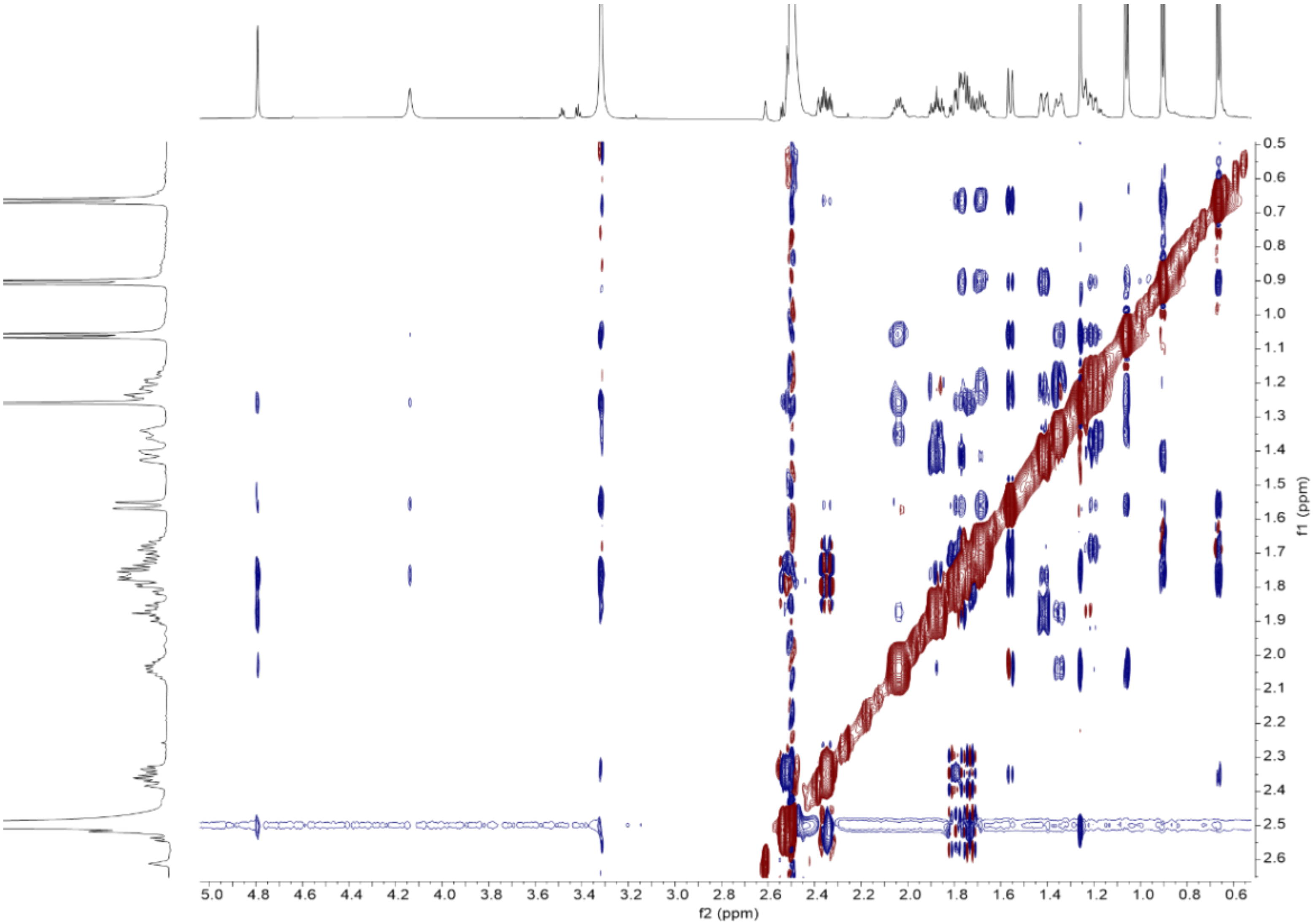

Figure S16. NOESY spectrum of **1** in DMSO-d_6

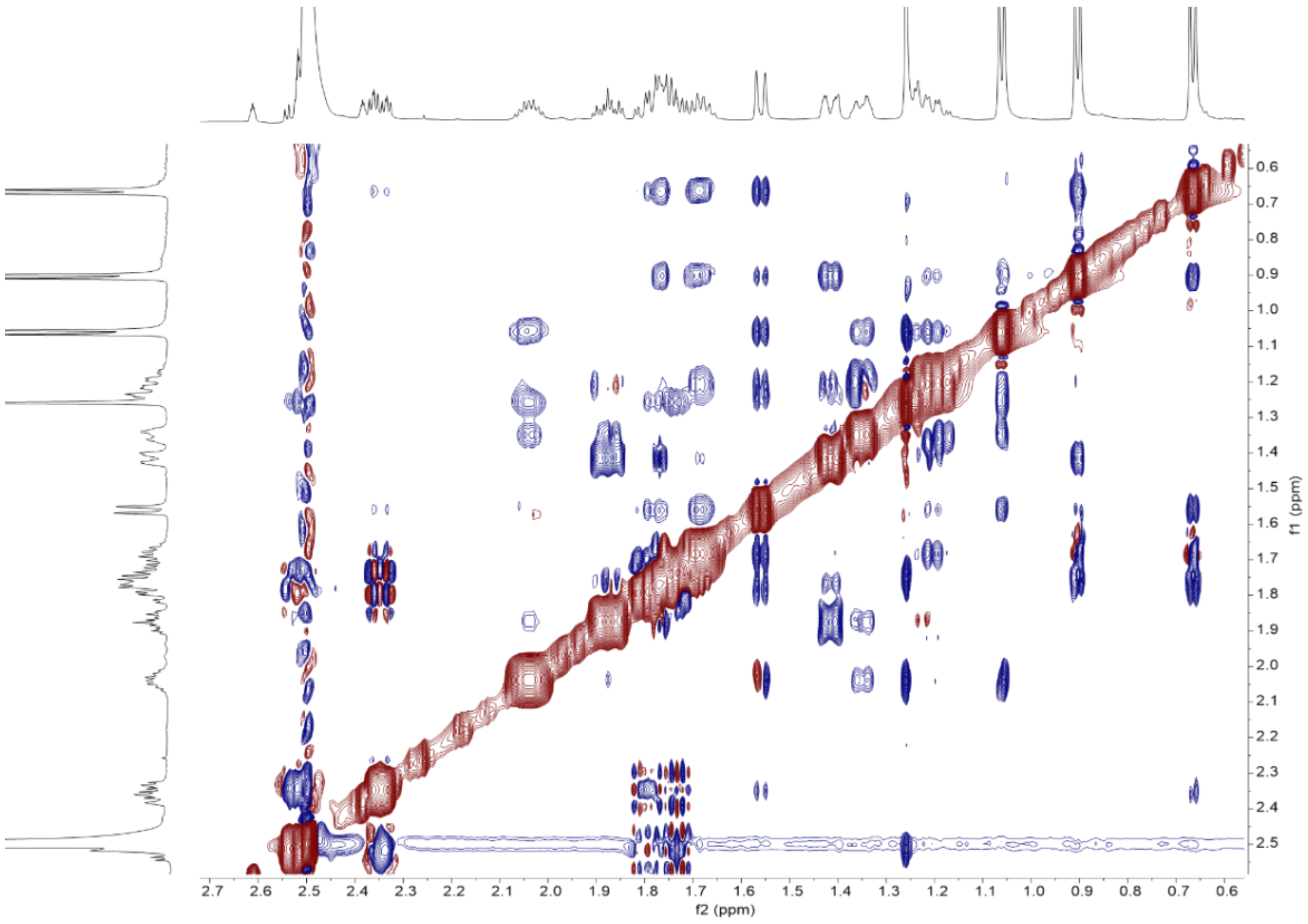

Figure S17. NOESY spectrum of **1** in DMSO-d_6 (expanded)

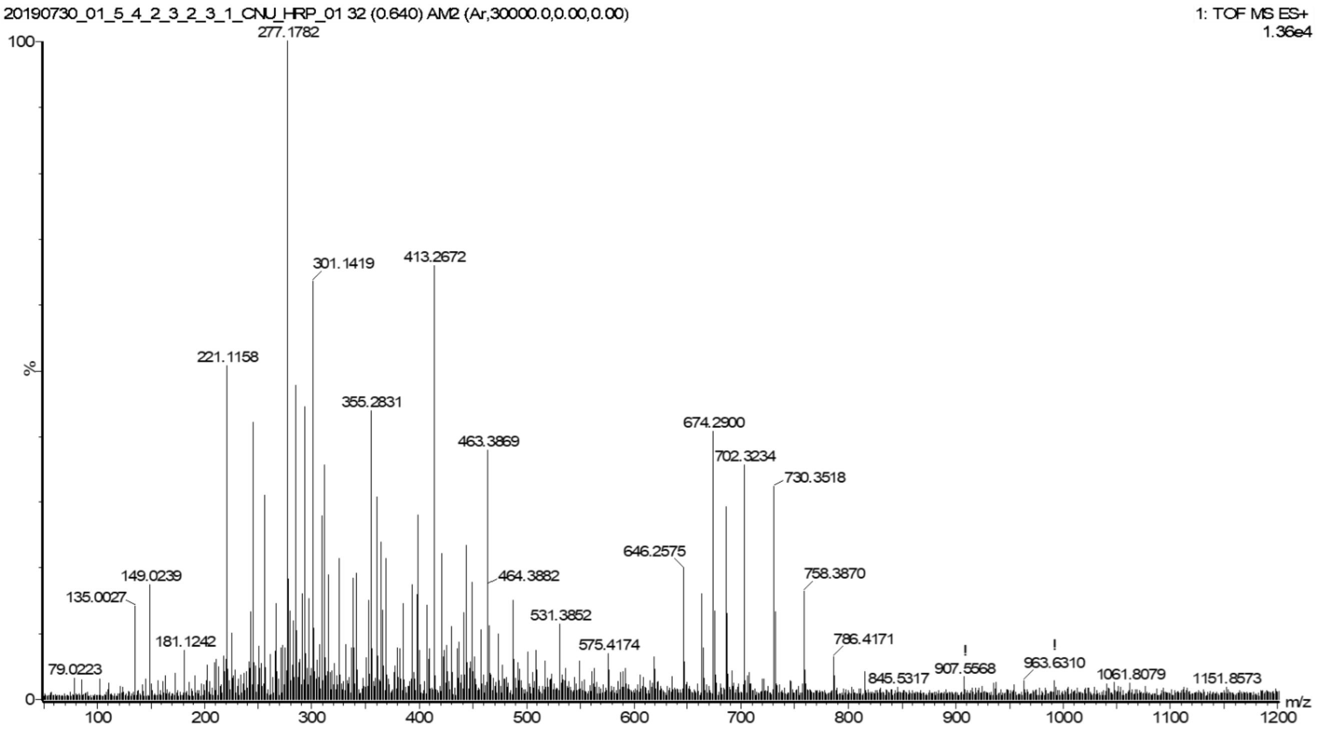

Figure S18. HRESIMS of **2**

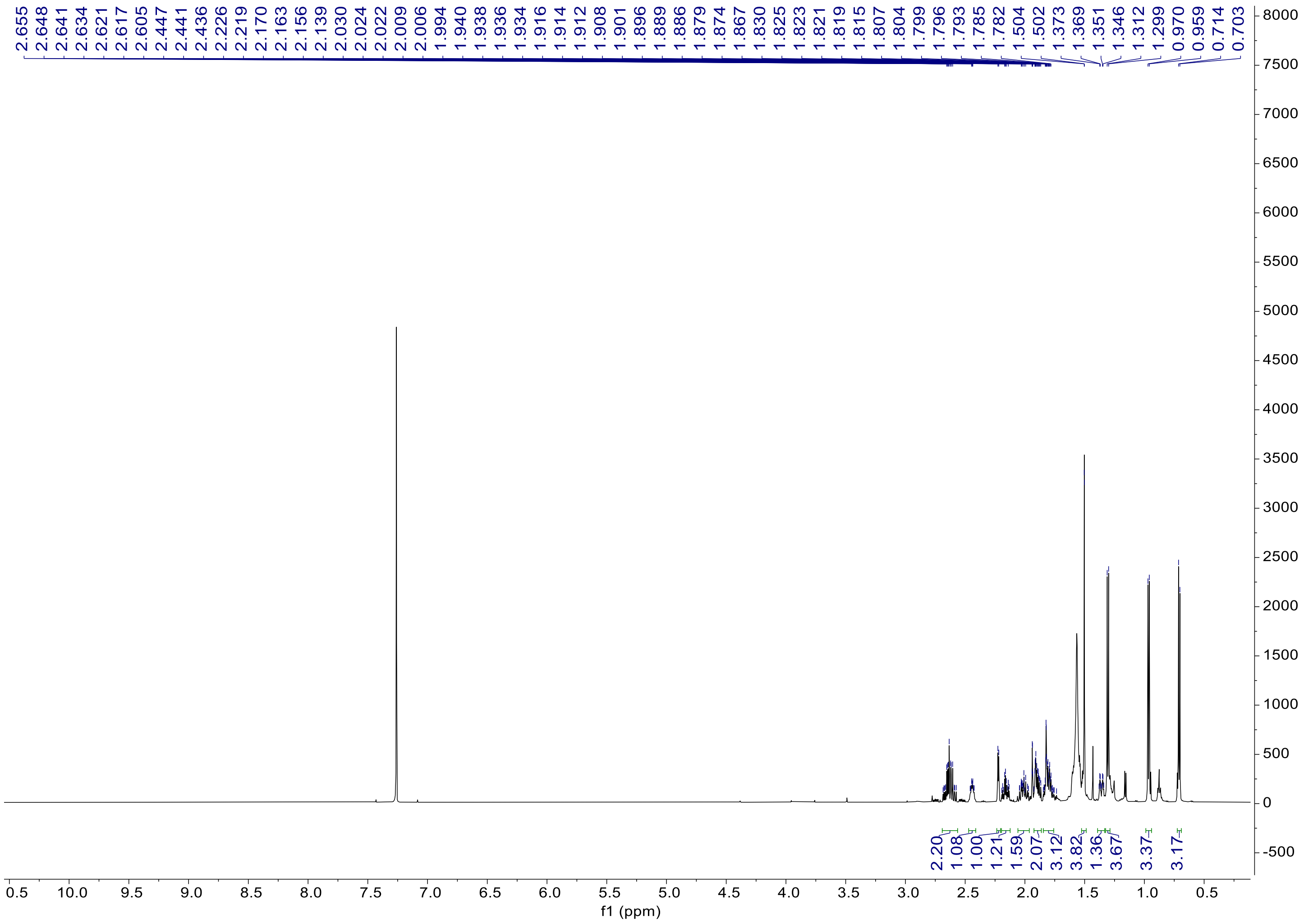

Figure S19. ^{1}H NMR data of **2** in $CDCl_3$

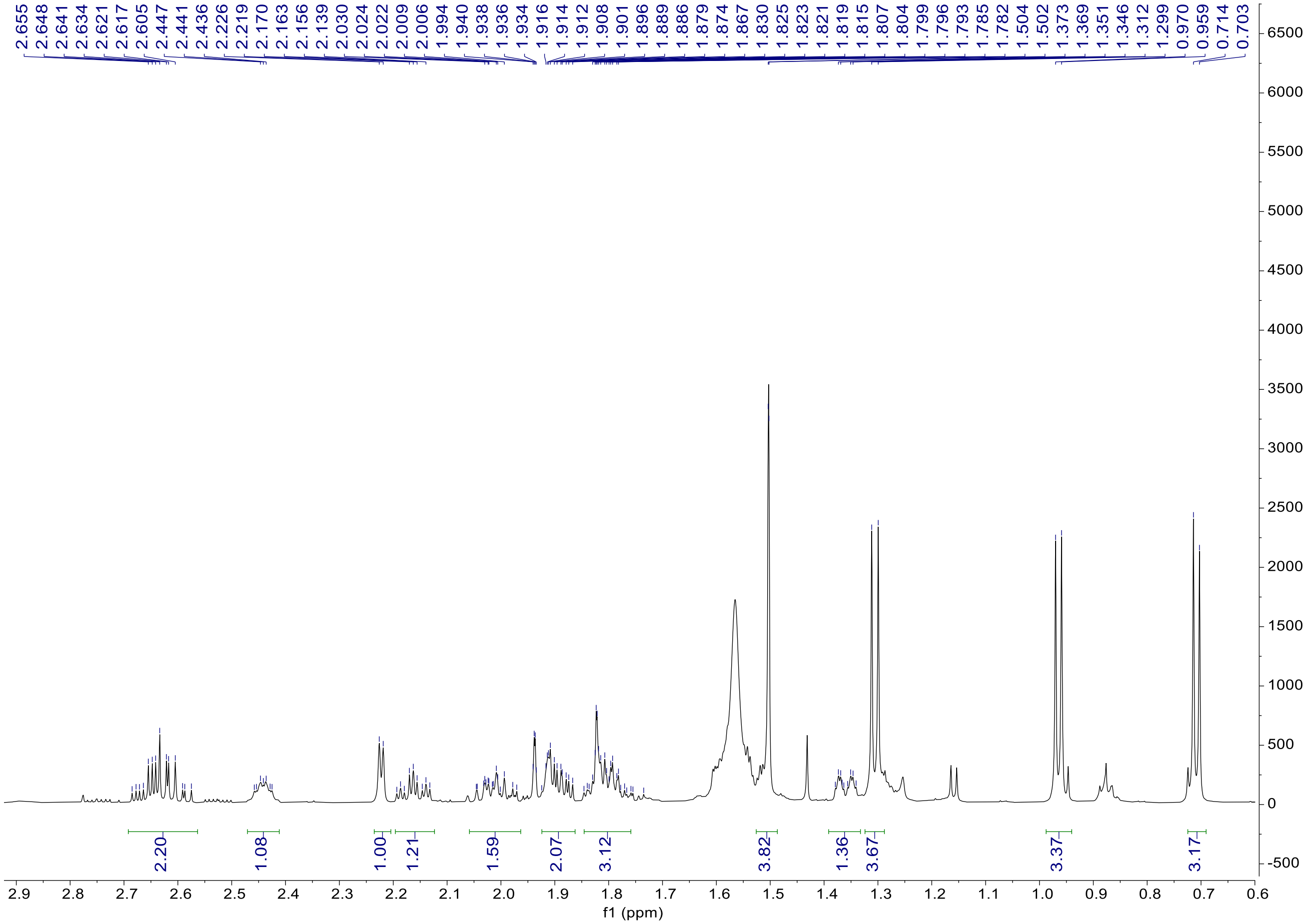

Figure S20. ^{1}H NMR data of **2** in $CDCl_3$ (expanded)

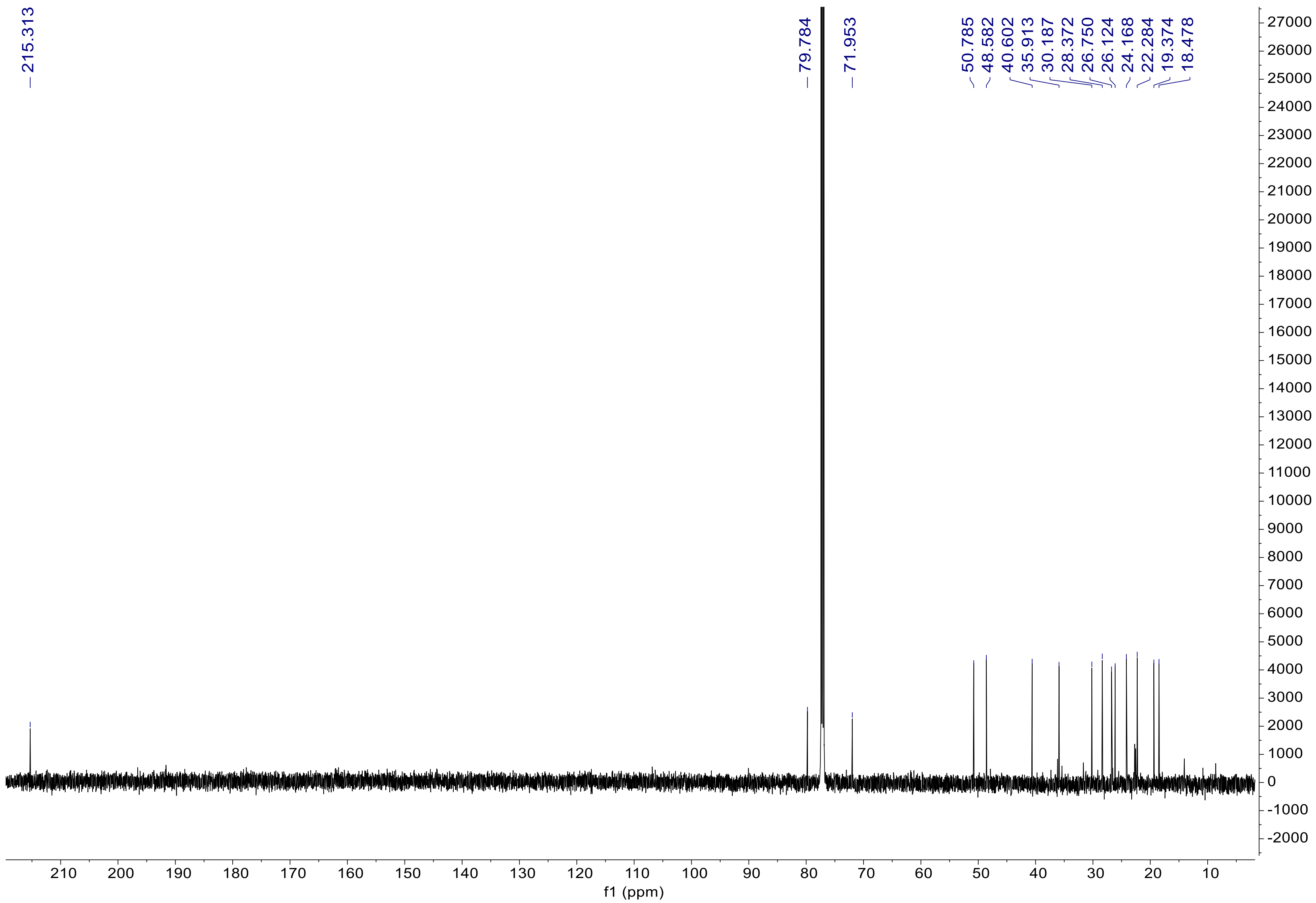

Figure S21. ^{13}C NMR data of **2** in $CDCl_3$

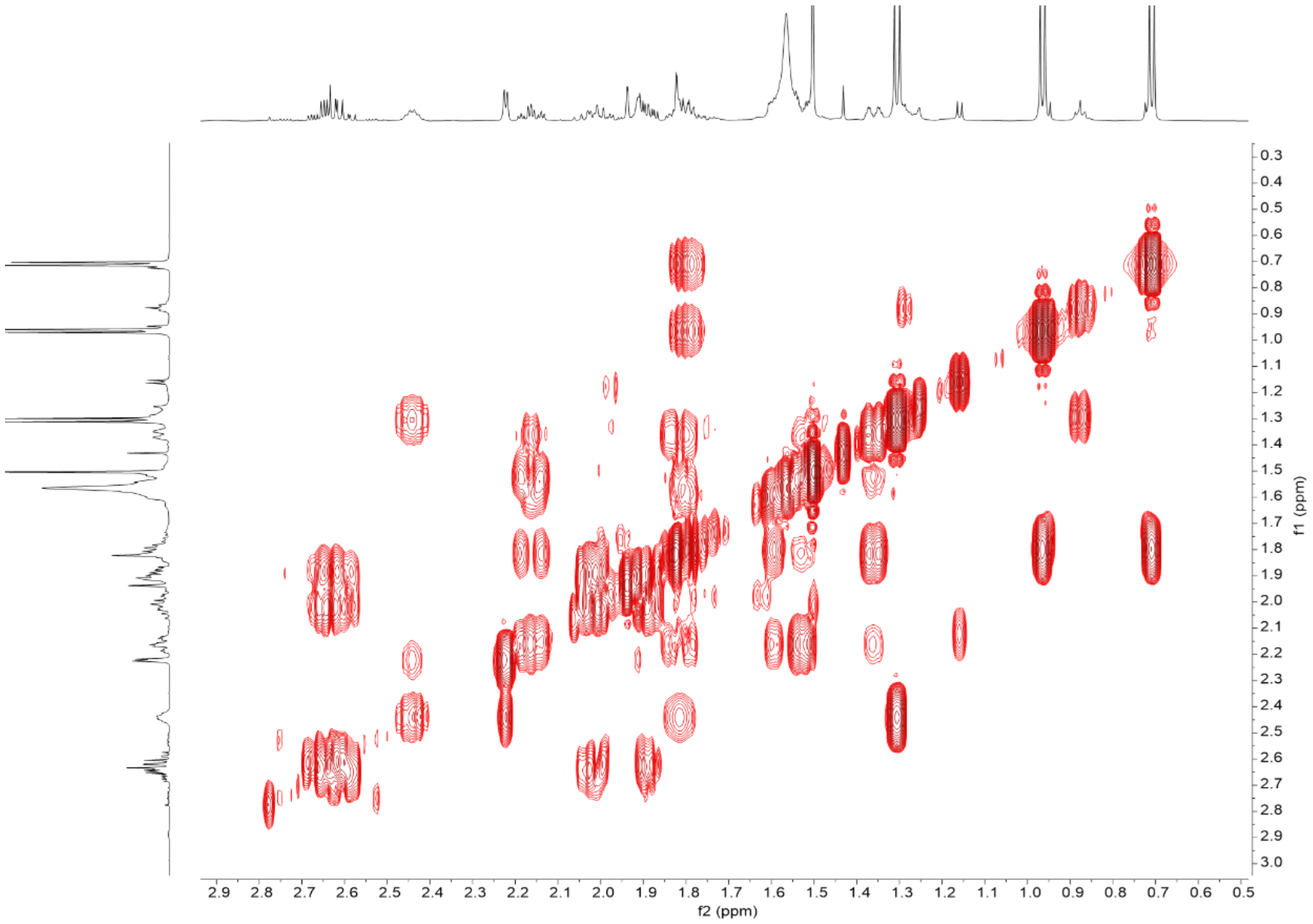

Figure S22. COSY spectrum of **2** in $CDCl_3$

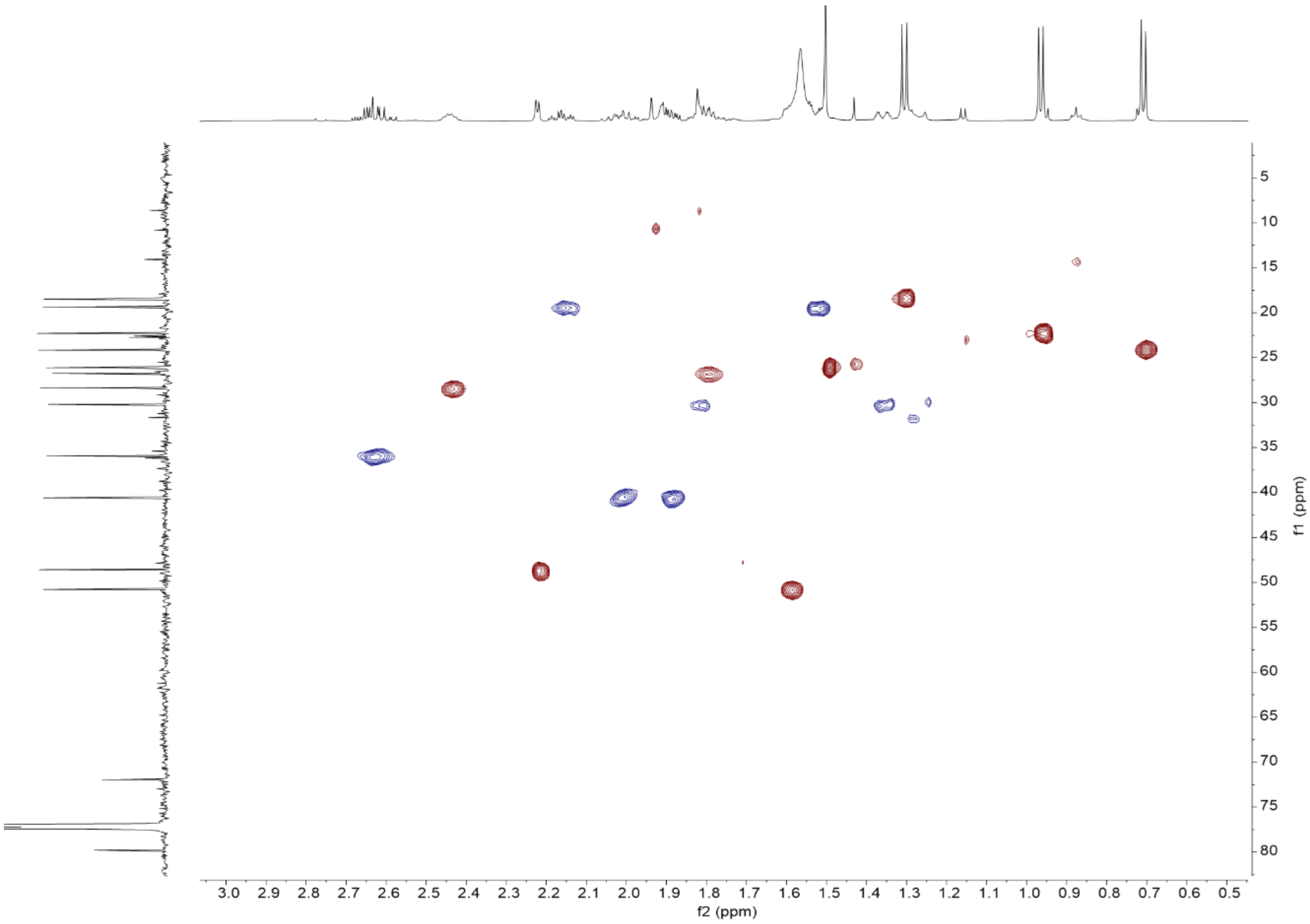

Figure S23. HSQC spectrm of **2** in $CDCl_3$

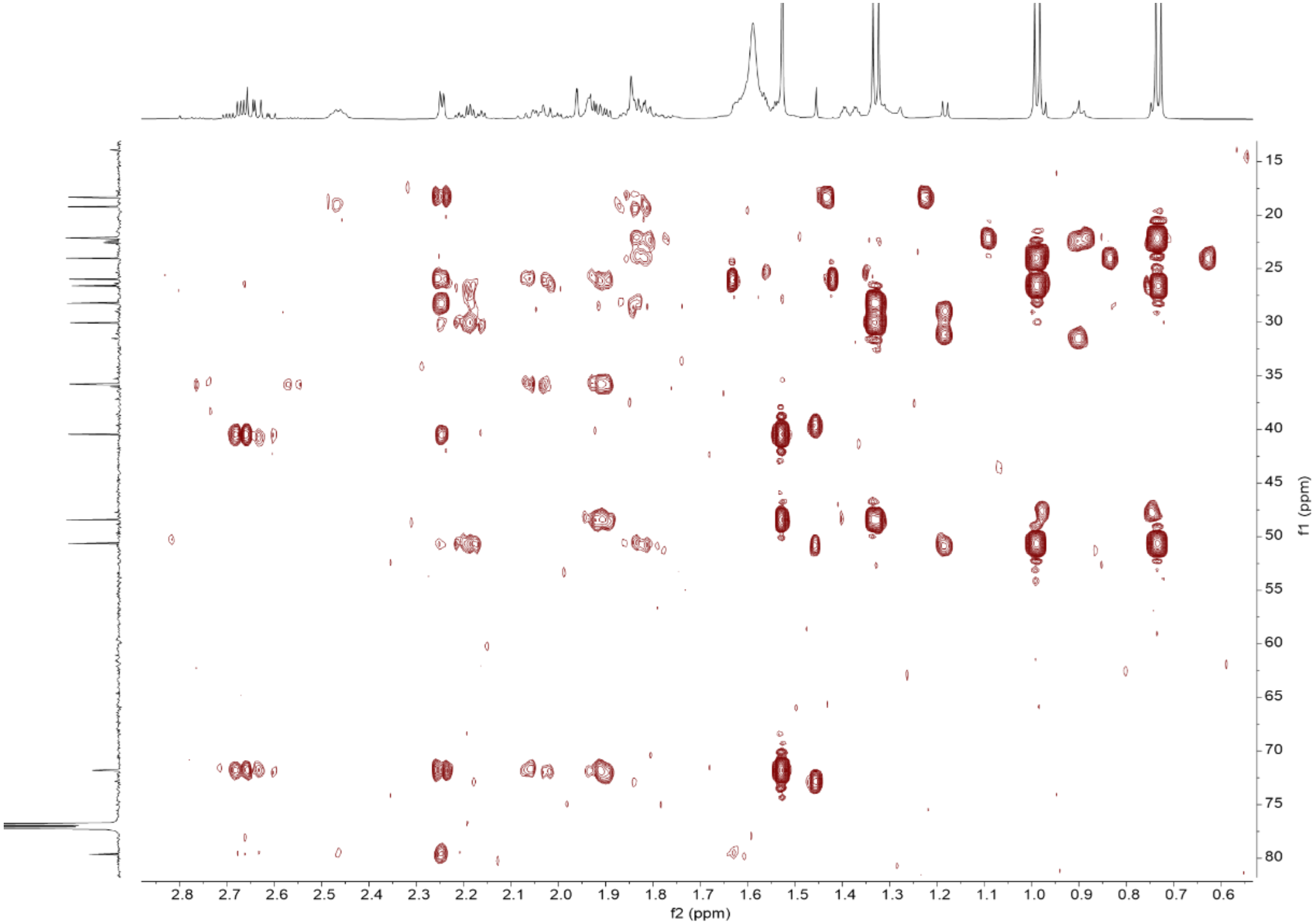

Figure S24. HMBC spectrum of **2** in $CDCl_3$ (expanded - 1)

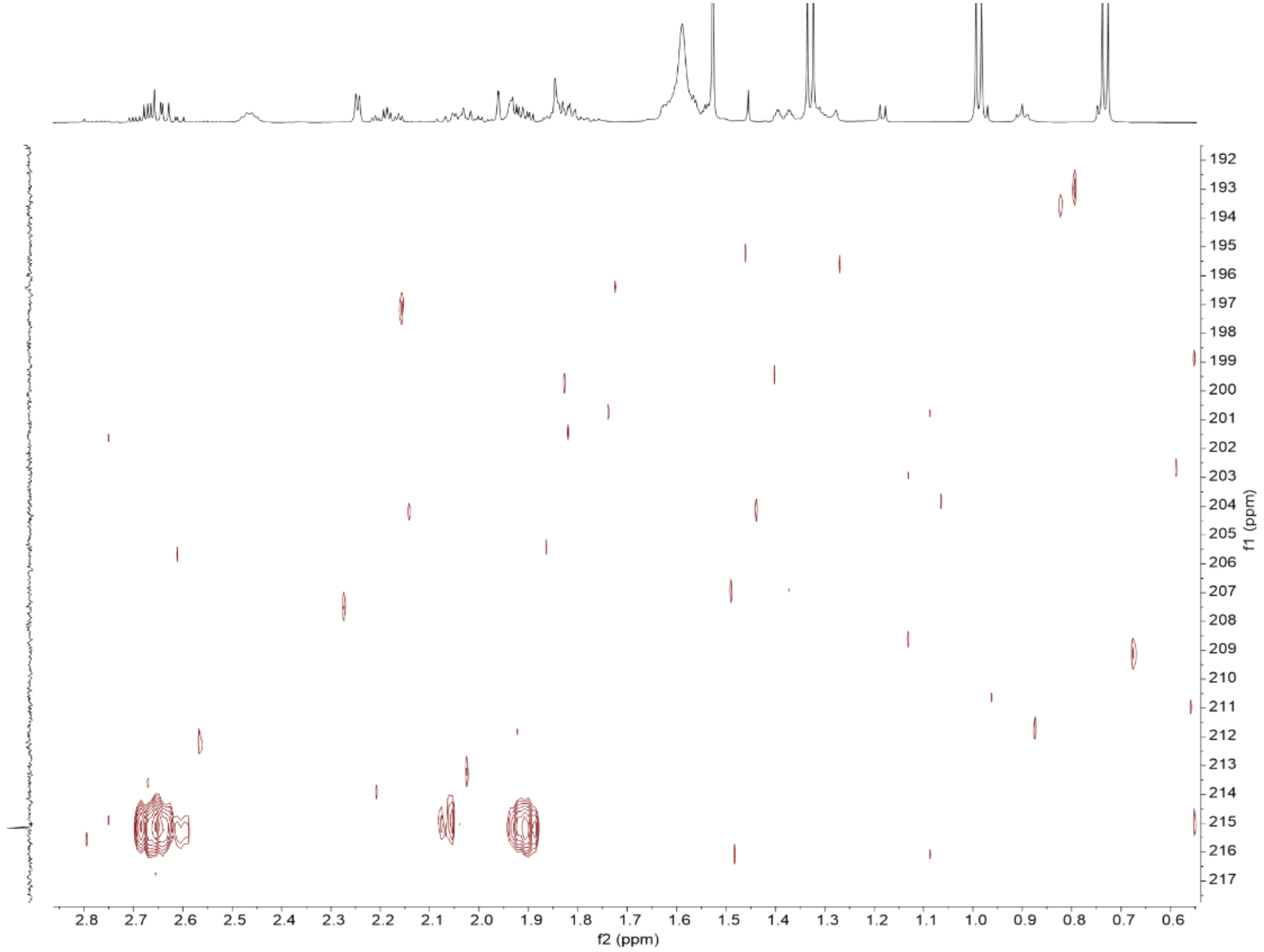

Figure S25. HMBC spectrum of **2** in $CDCl_3$ (expanded - 2)

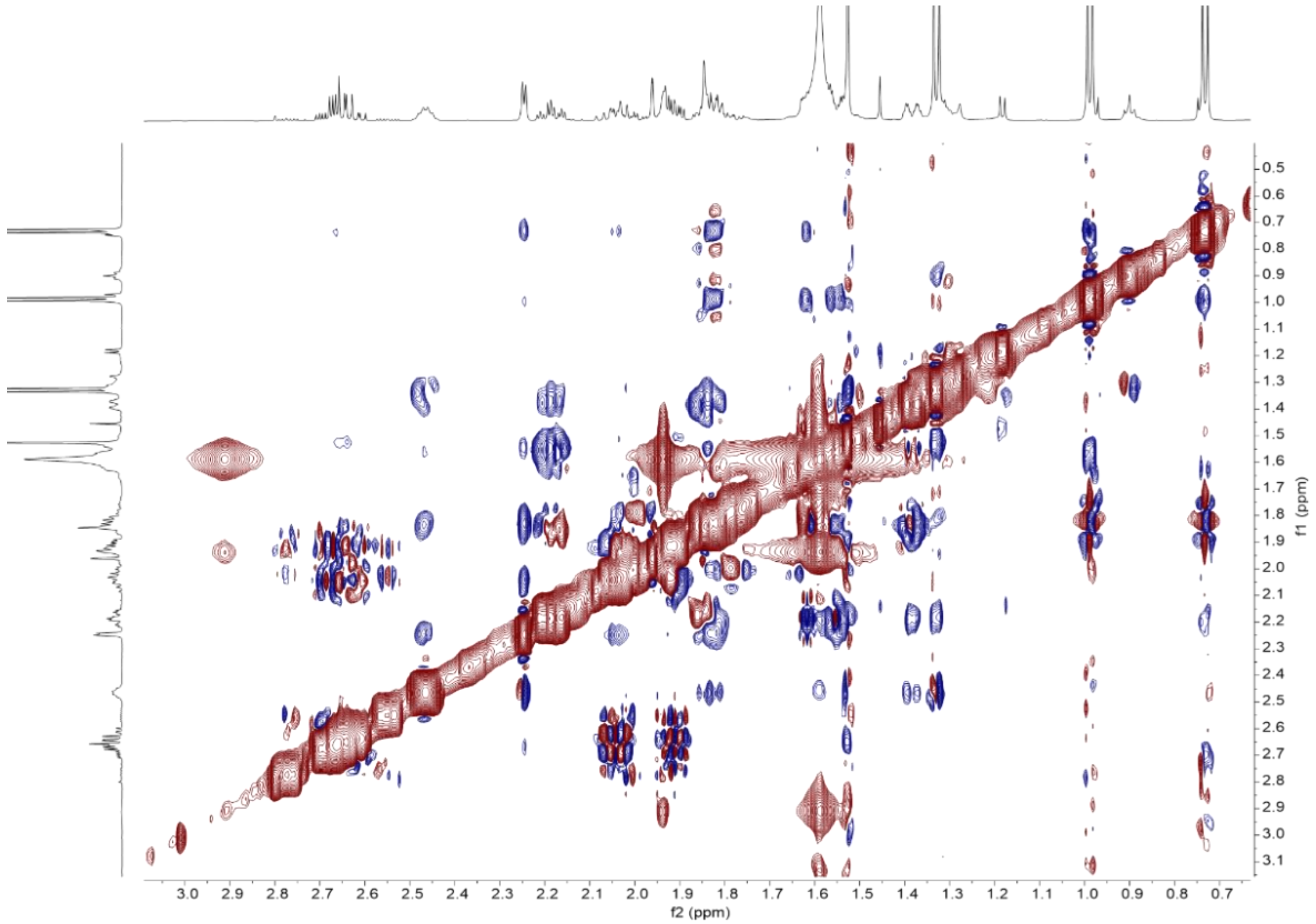

Figure S26. NOESY spectrum of **2** in $CDCl_3$

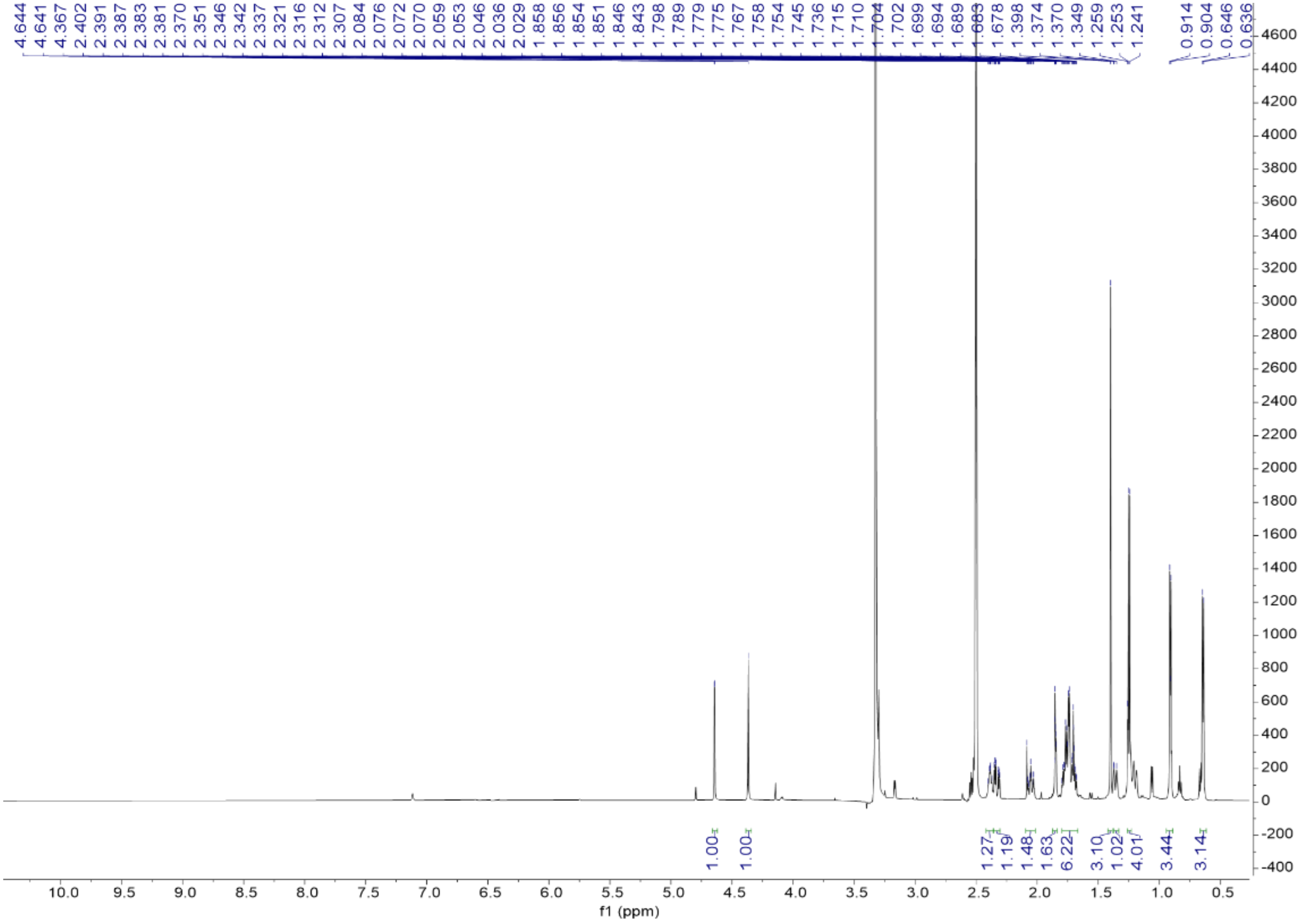

Figure S27. ^{1}H NMR spectrum of **2** in DMSO-d_6

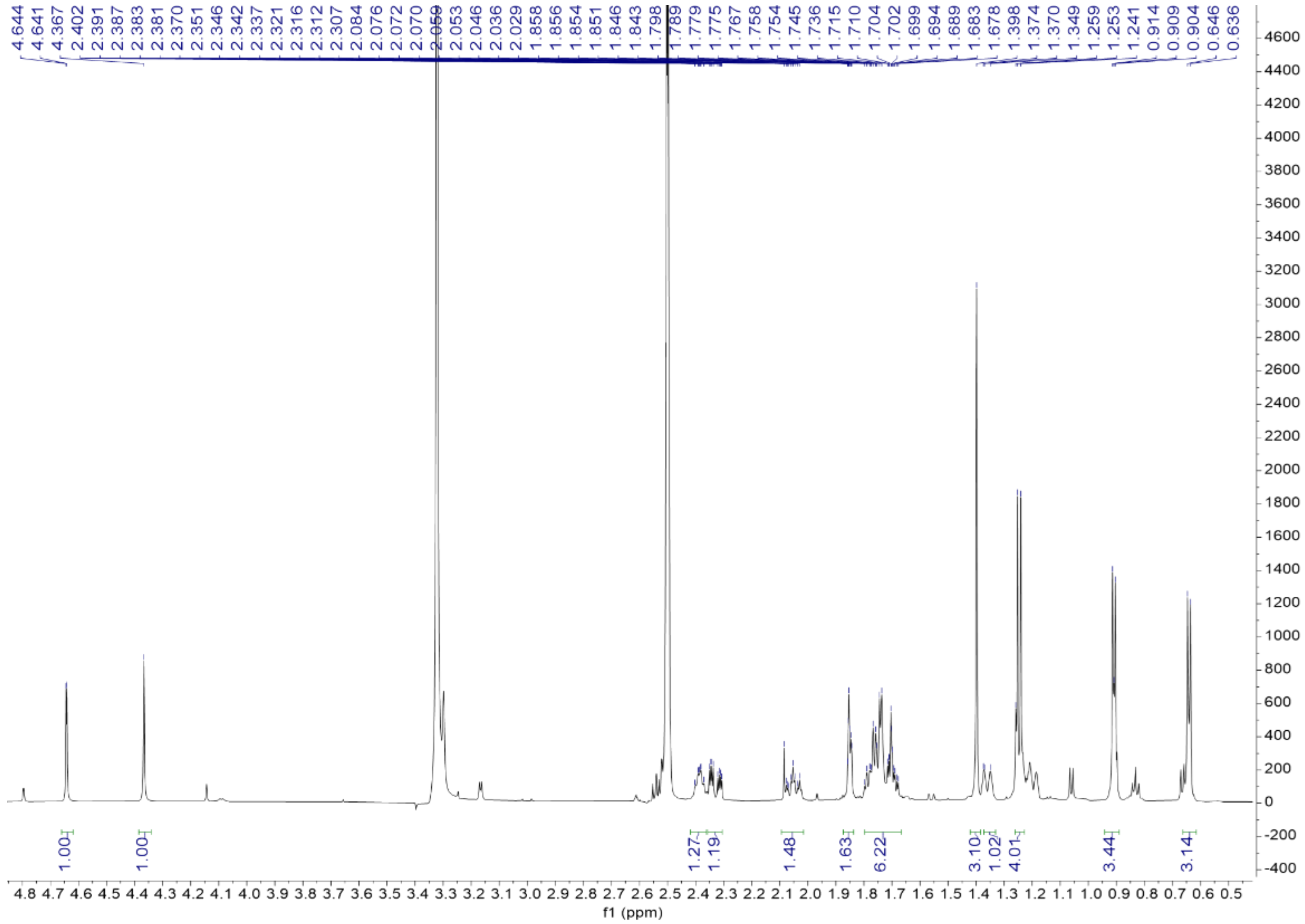

Figure S28. ^{1}H NMR spectrum of **2** in DMSO-*d*$_6$ (expanded)

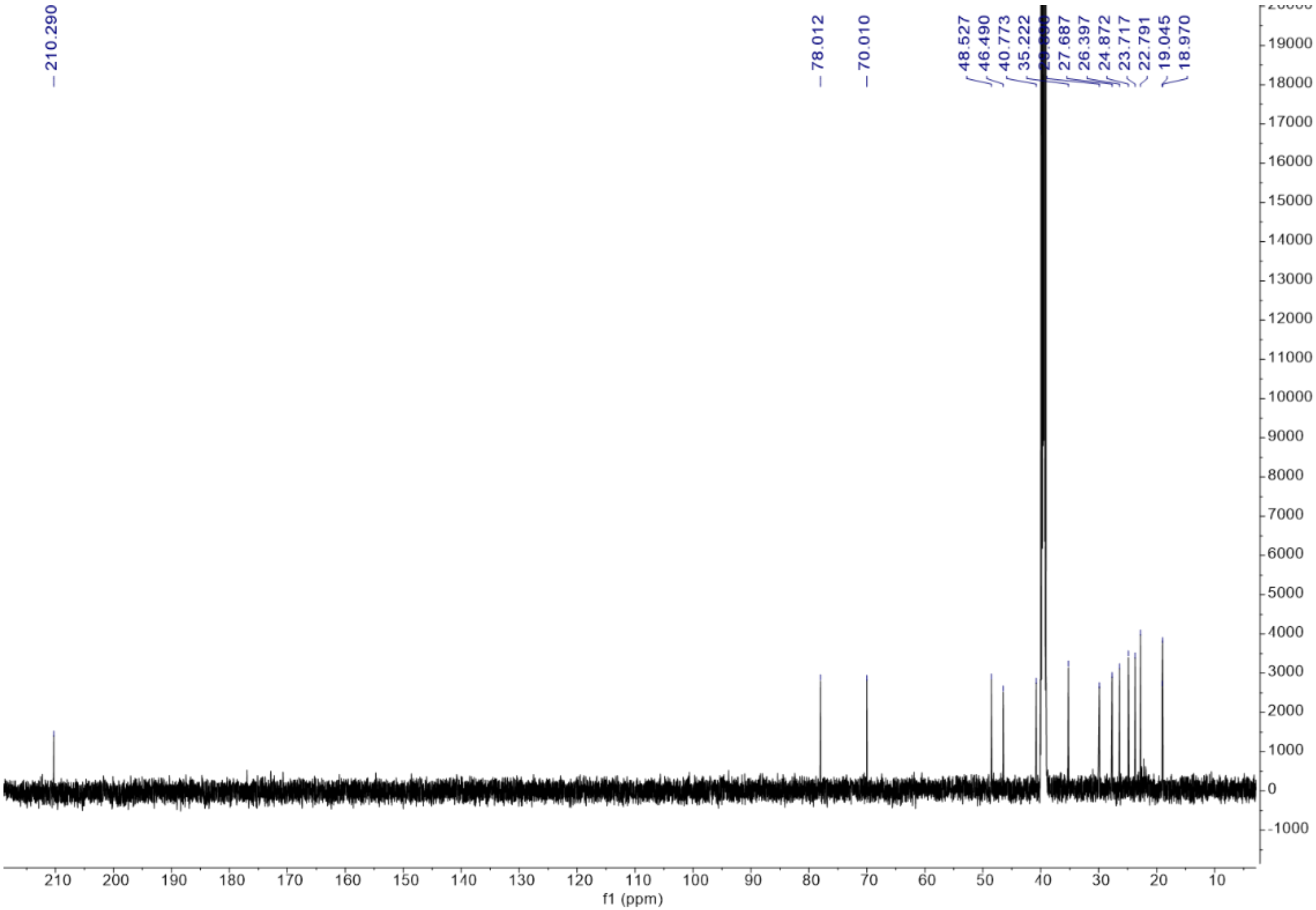

Figure S29. ^{13}C NMR spectrum of **2** in DMSO-d_6

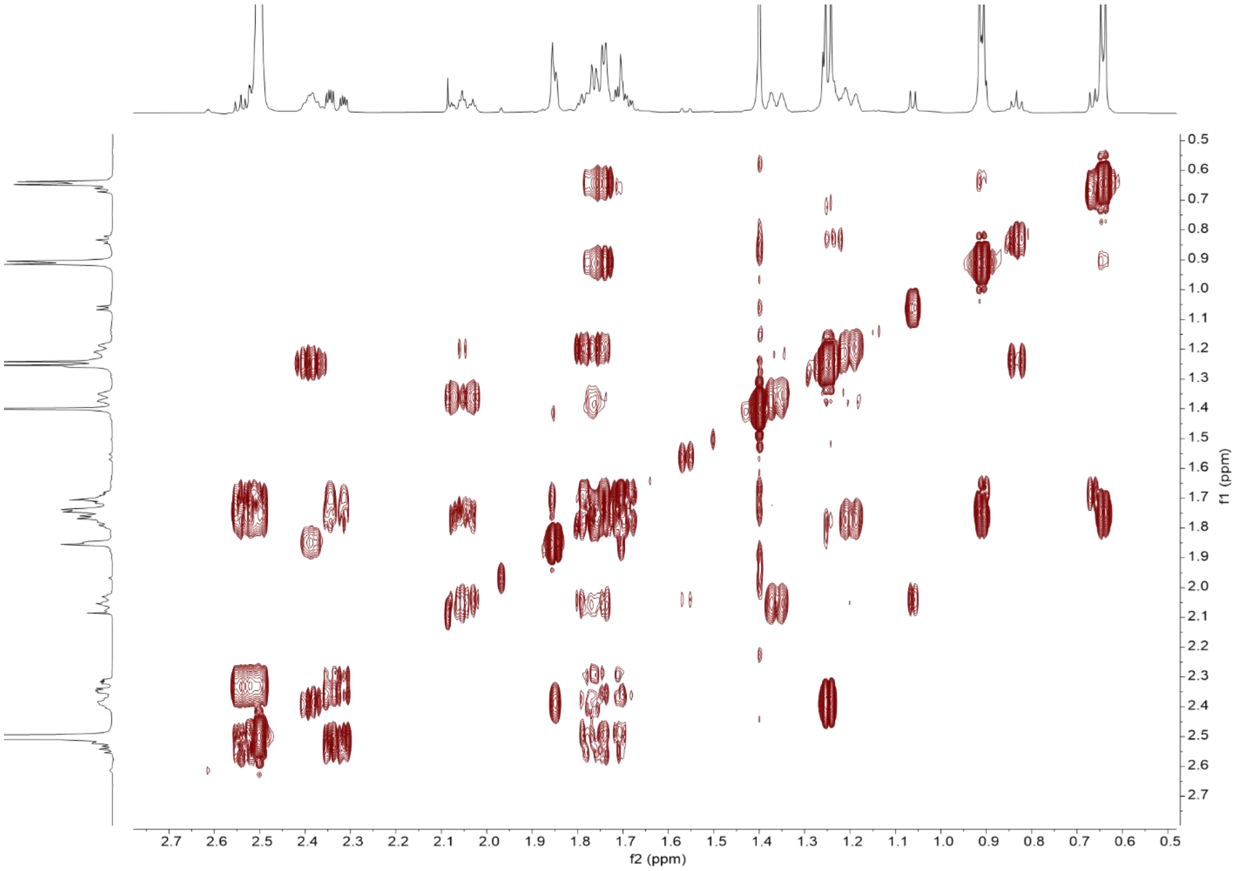

Figure S30. COSY spectrum of **2** in DMSO-d_6

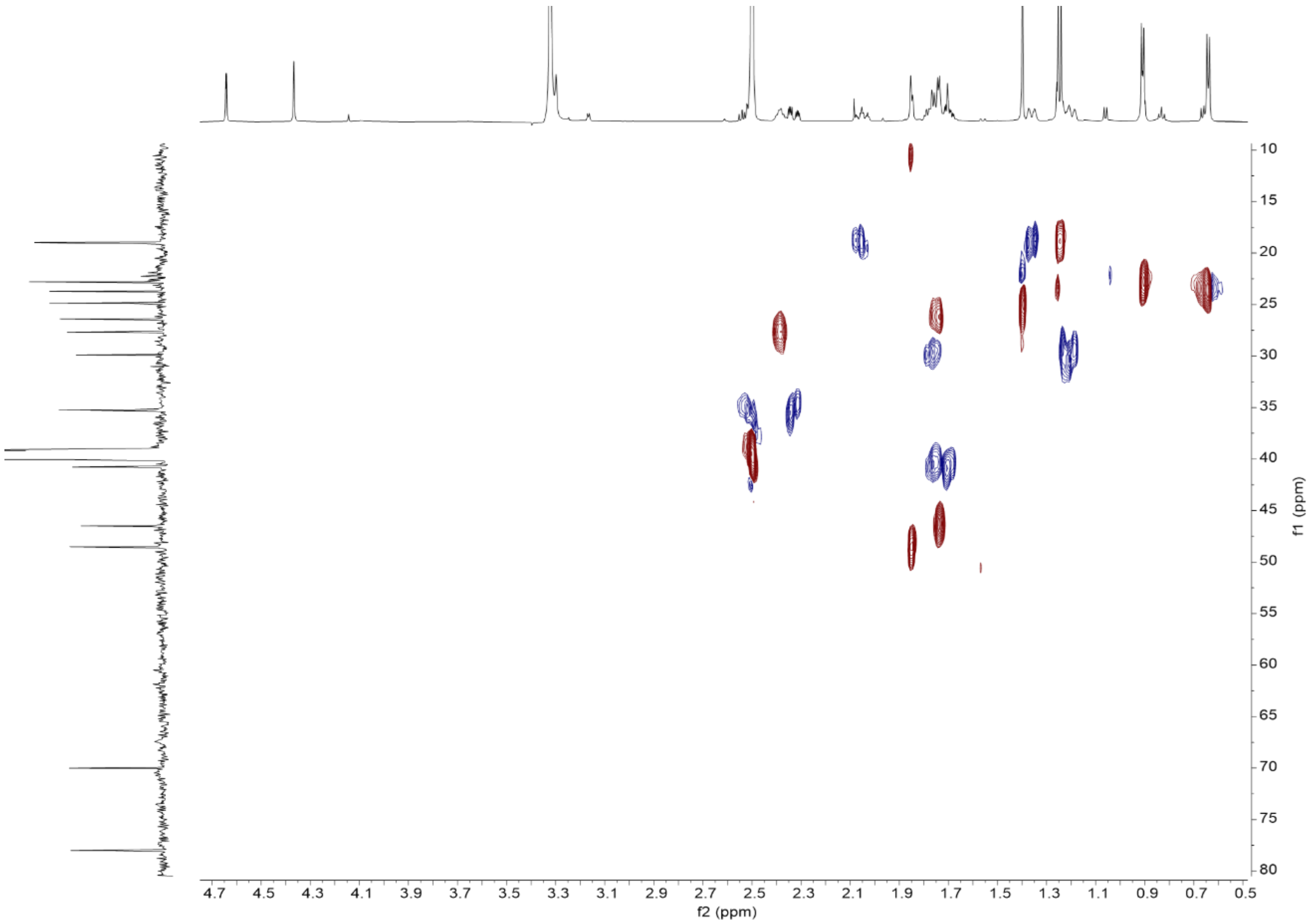

Figure S31. HSQC spectrum of **2** in DMSO-*d*6

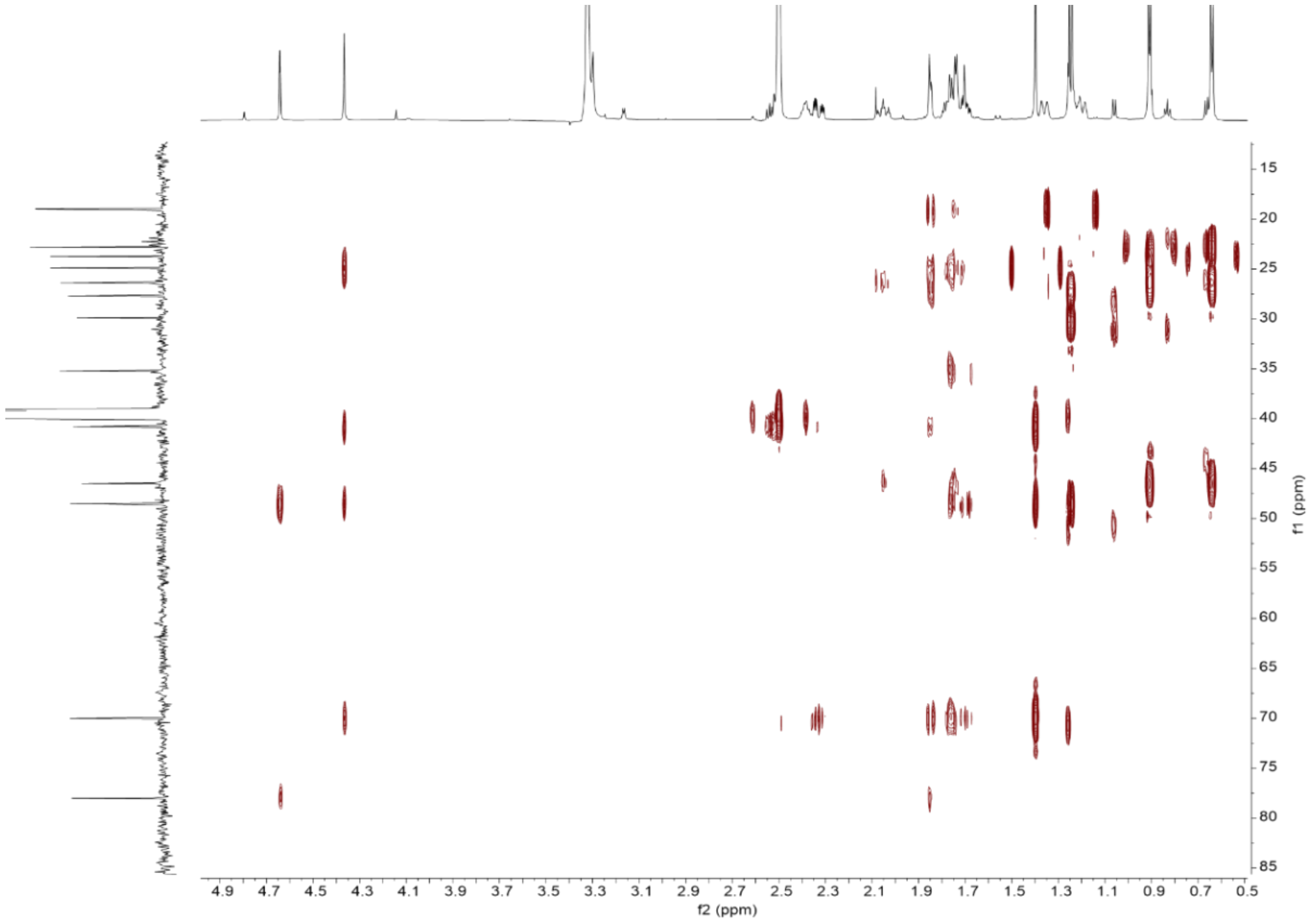

Figure S32. HMBC spectrum of **2** in DMSO-d_6 (expanded – 1)

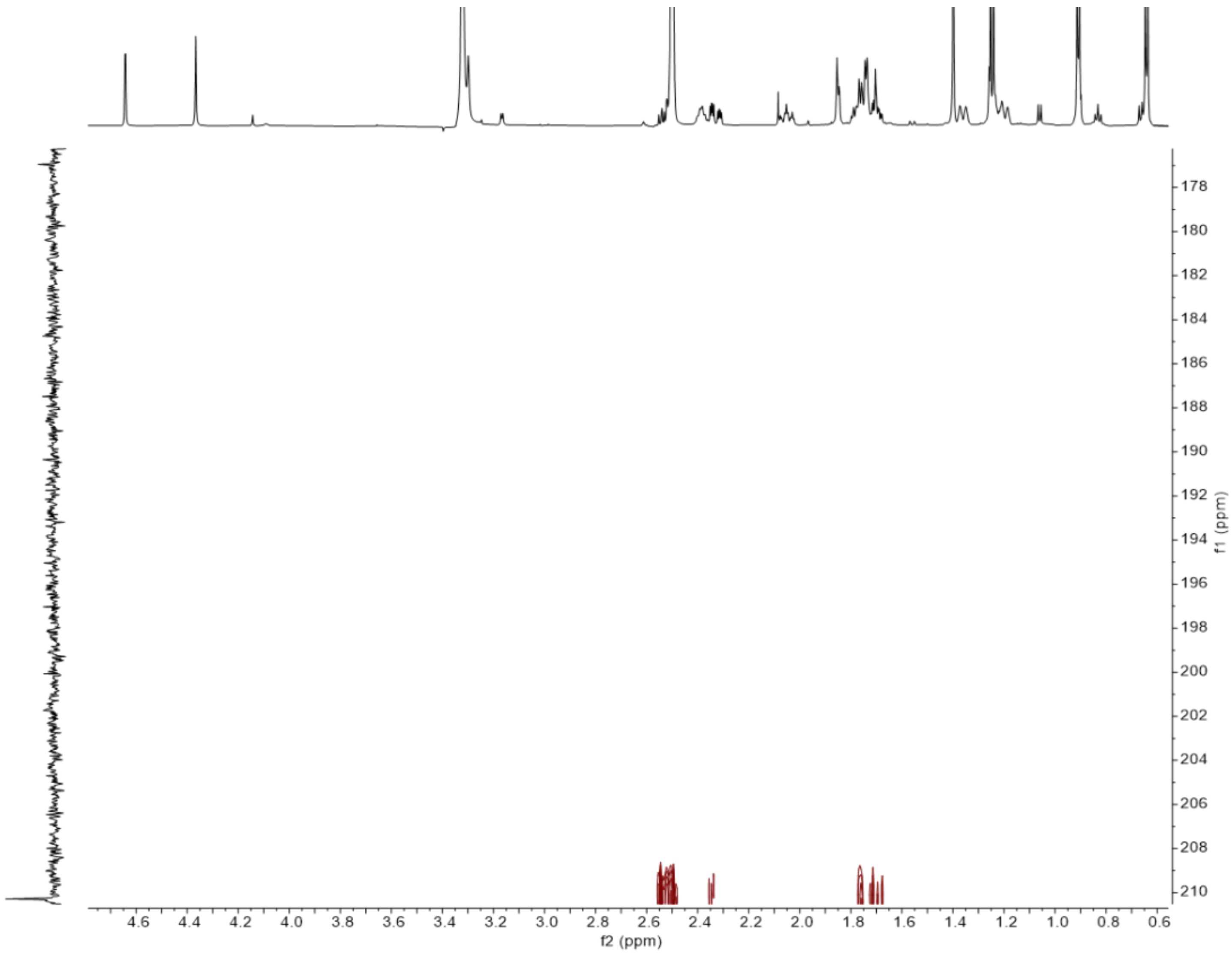

Figure S33. HMBC spectrum of **2** in DMSO-d_6 (expanded - 2)

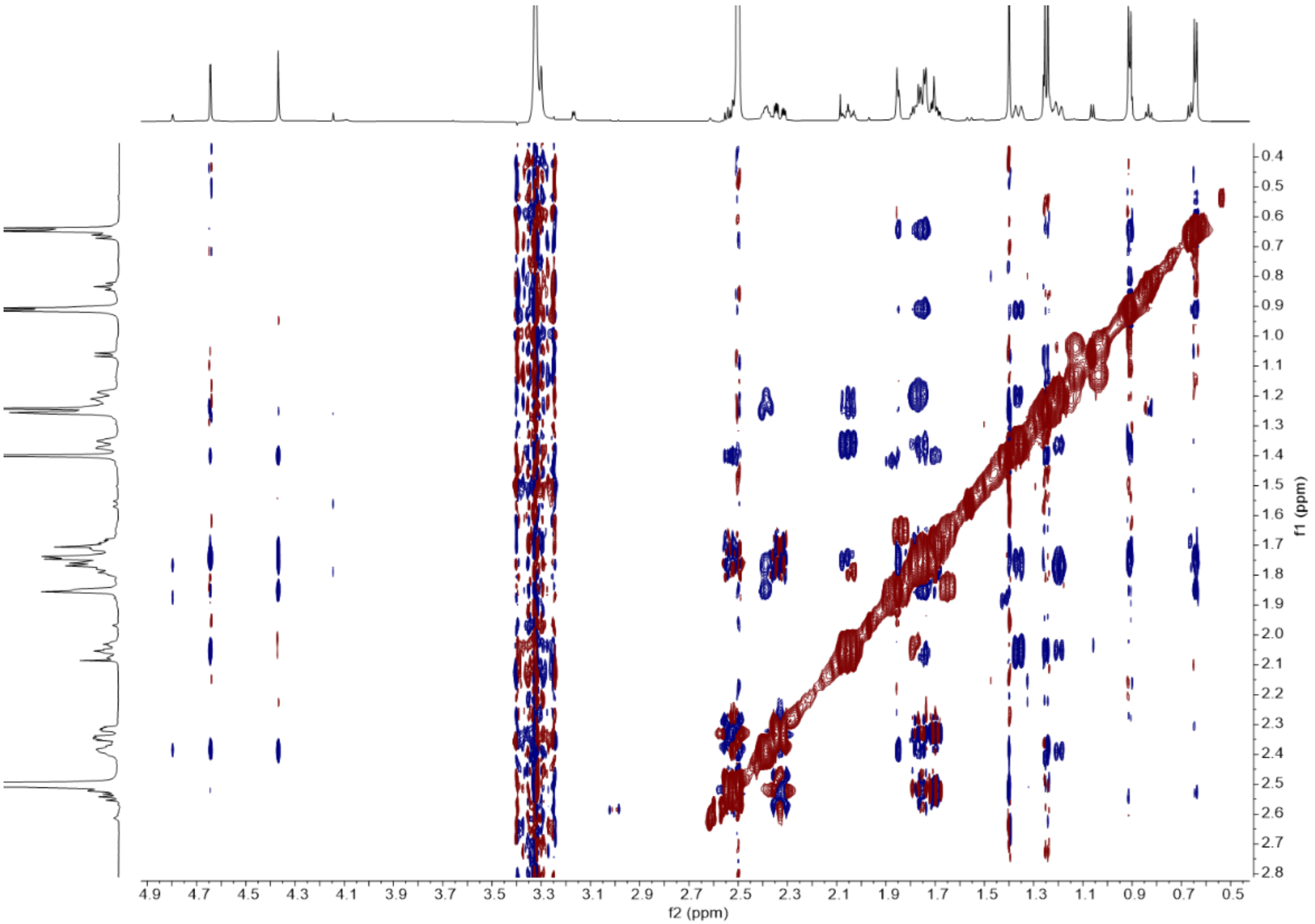

Figure S34. NOESY spectrum of **2** in DMSO-*d*6

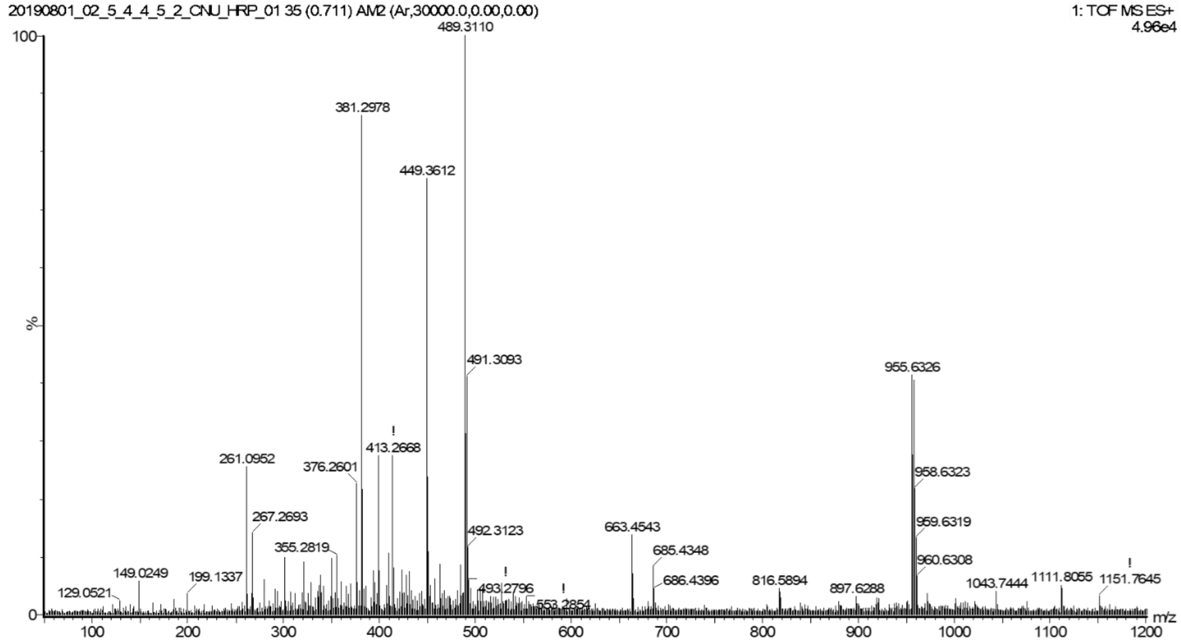

Figure S35. HRESIMS of **3**

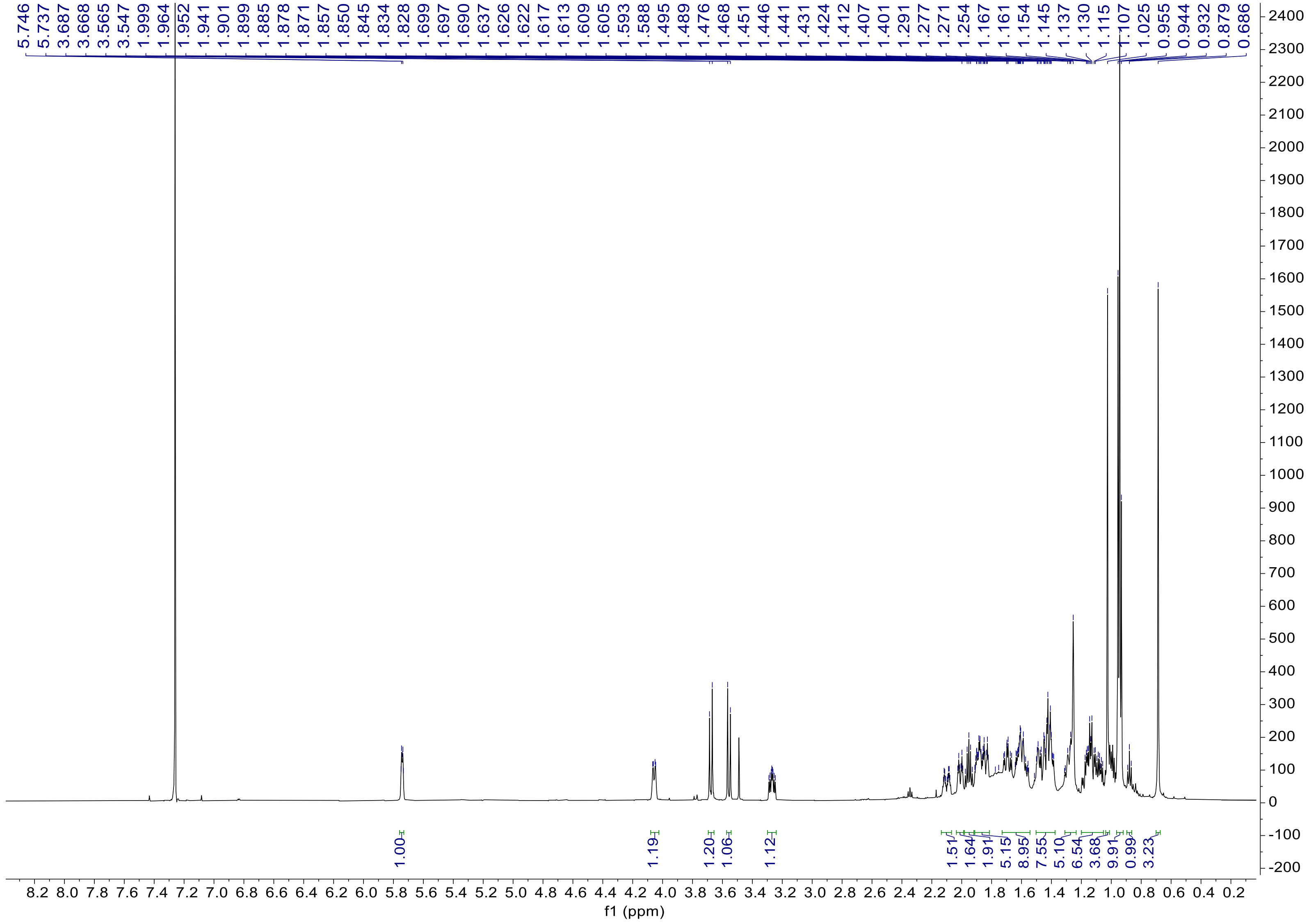

Figure S36. ^{1}H NMR data of **3** in $CDCl_3$

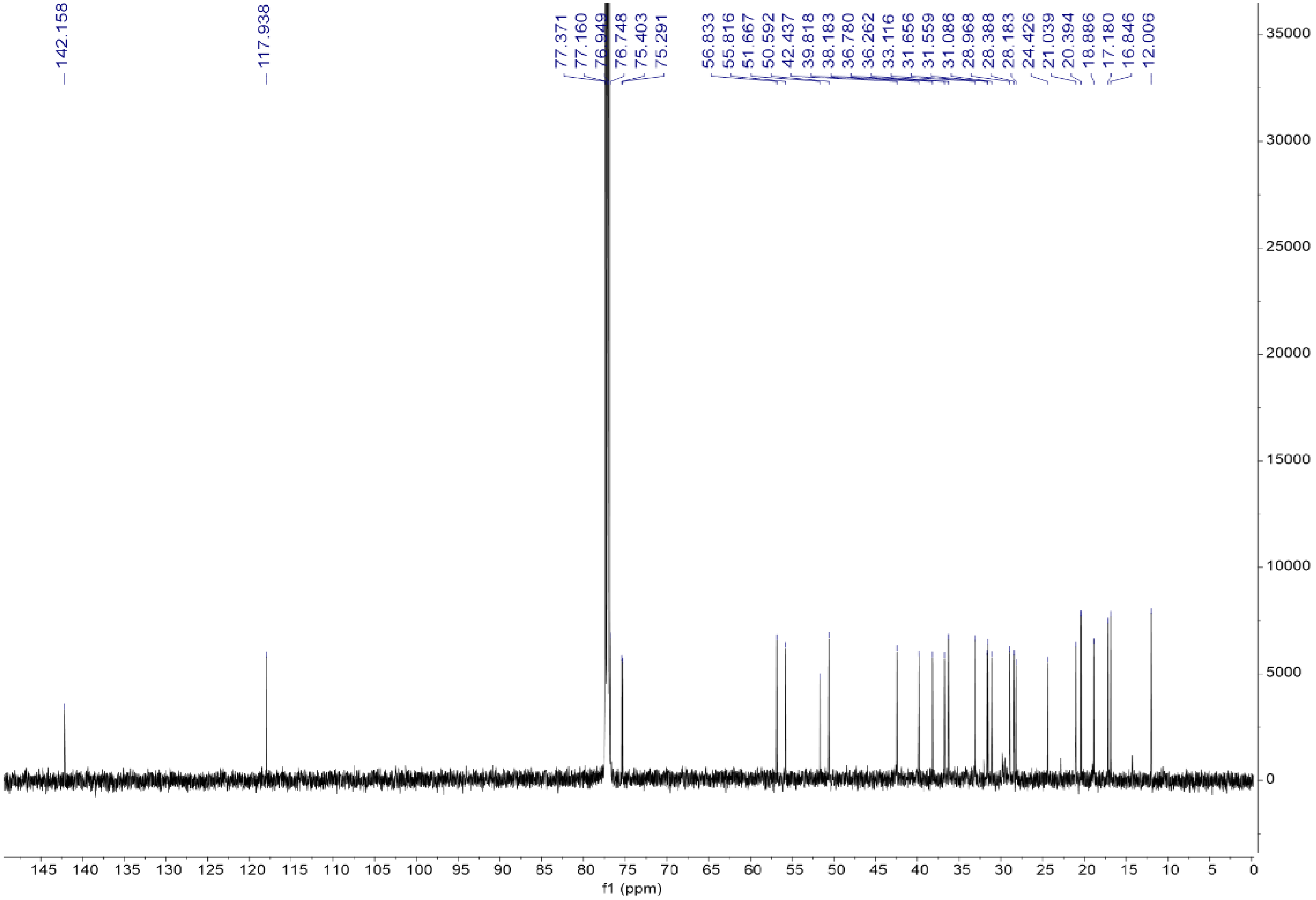

Figure S37. ^{13}C NMR data of **3** in $CDCl_3$

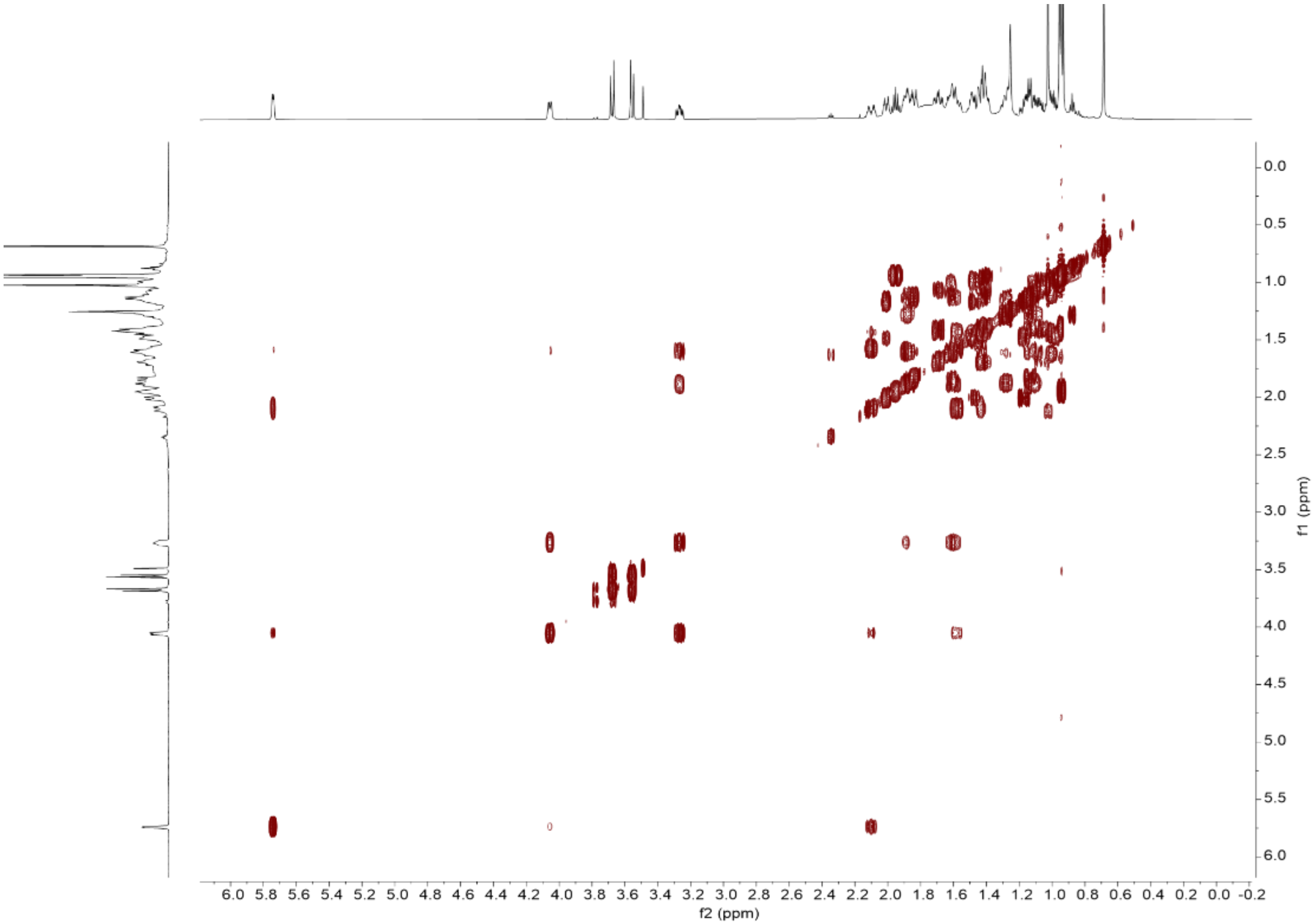

Figure S38. COSY spectrum of **3** in $CDCl_3$

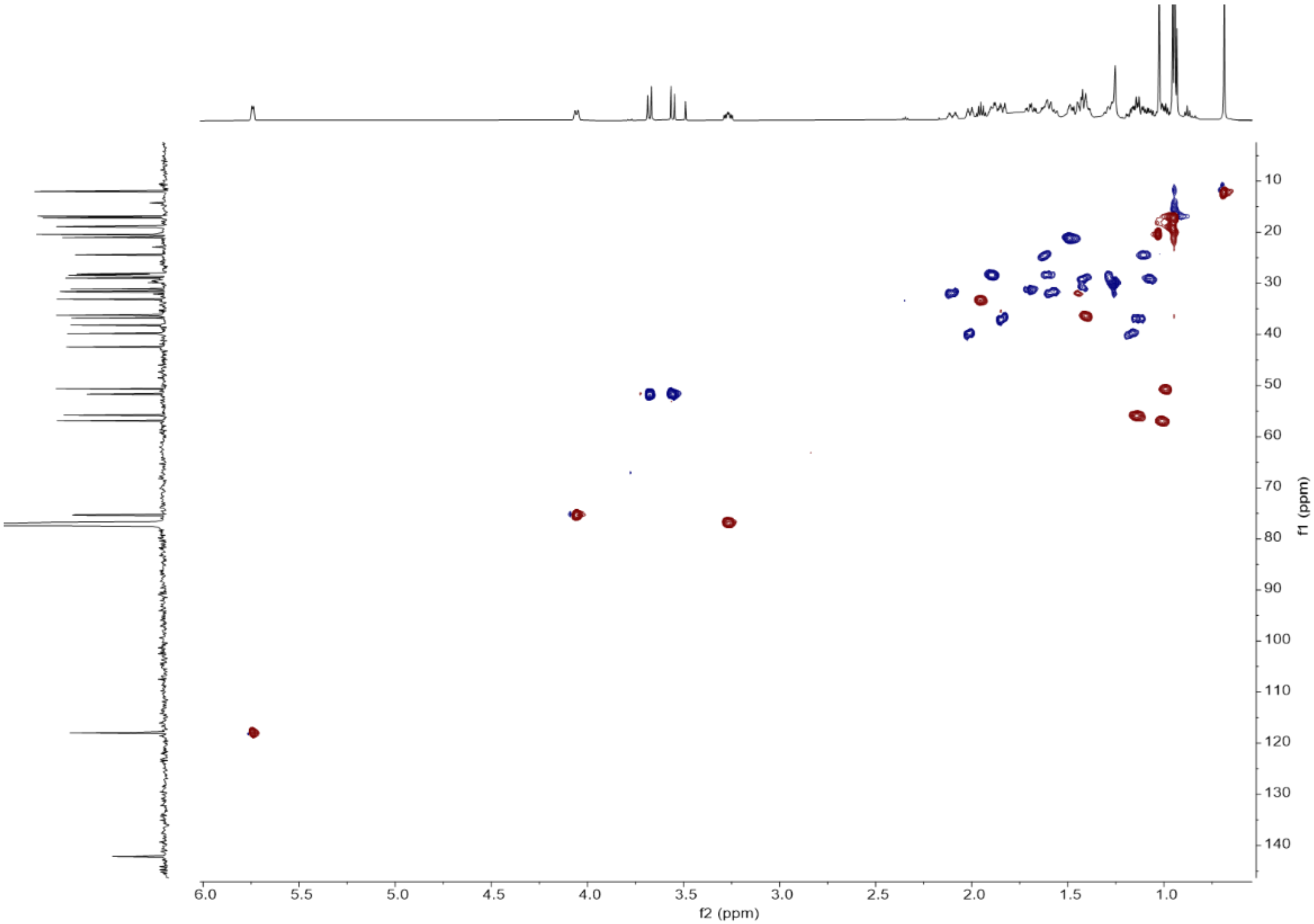

Figure S39. HSQC spectrum of **3** in $CDCl_3$

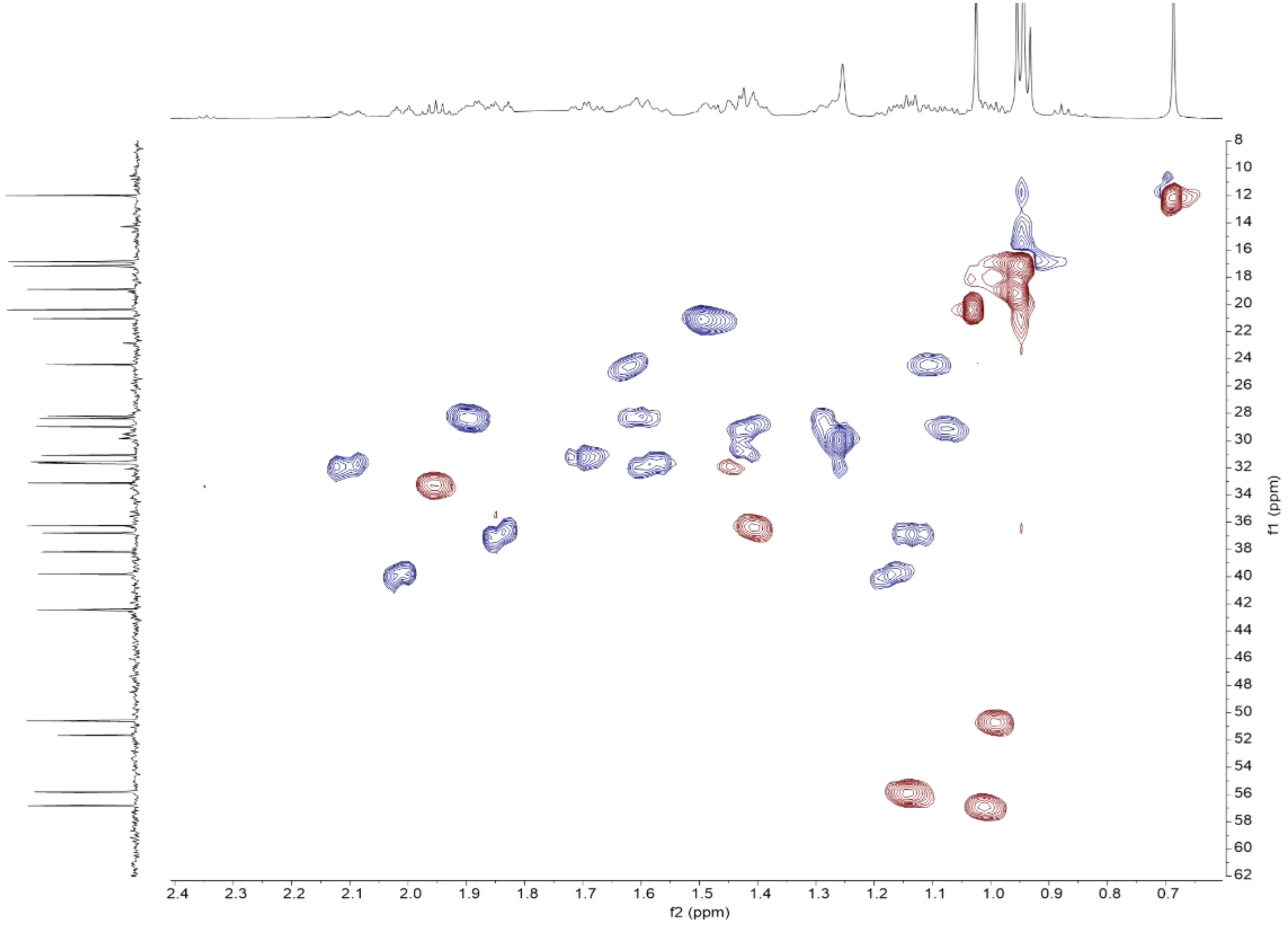

Figure S40. HSQC spectrum of **3** in $CDCl_3$ (expanded)

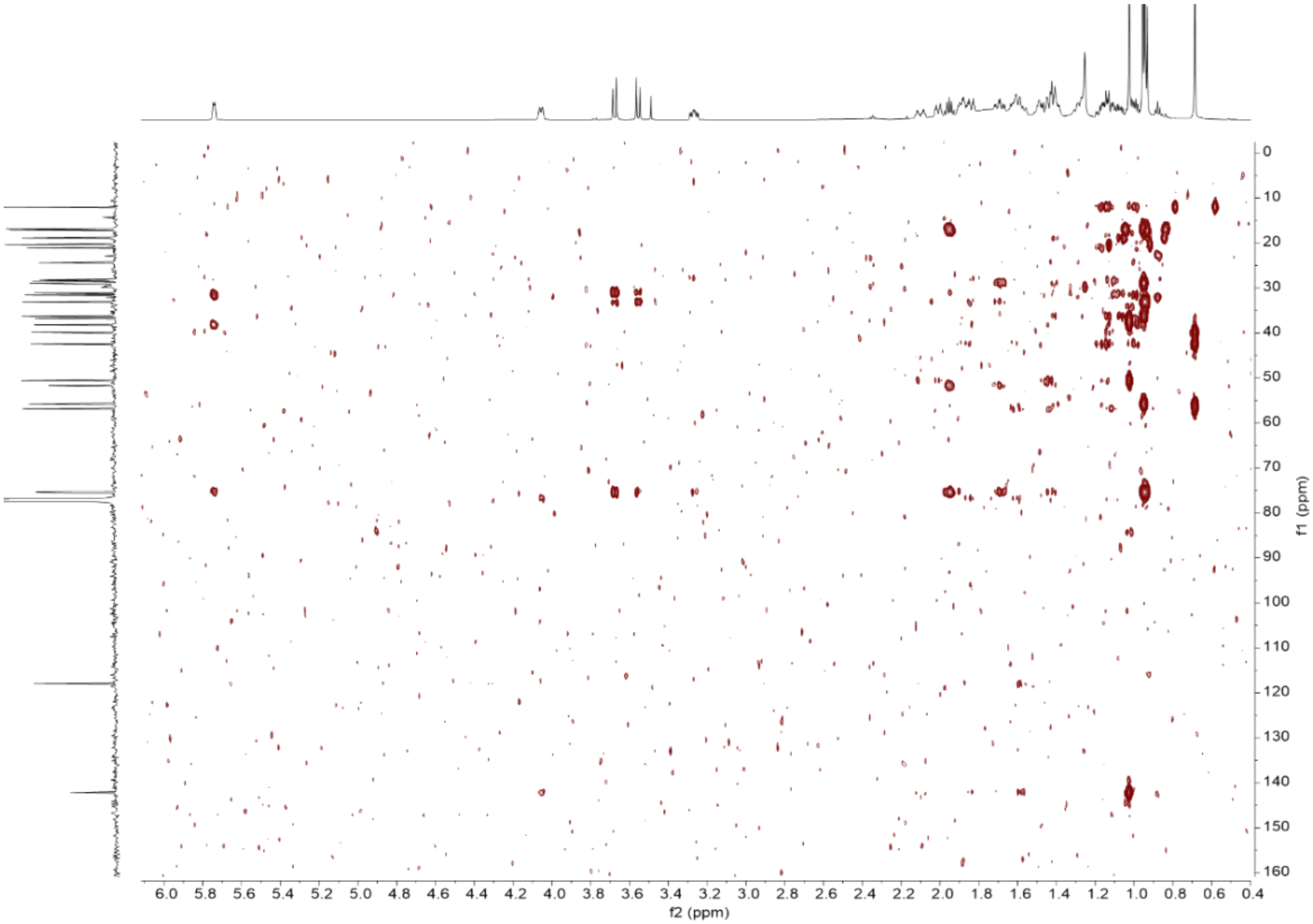

Figure S41. HMBC spectrum of **3** in $CDCl_3$

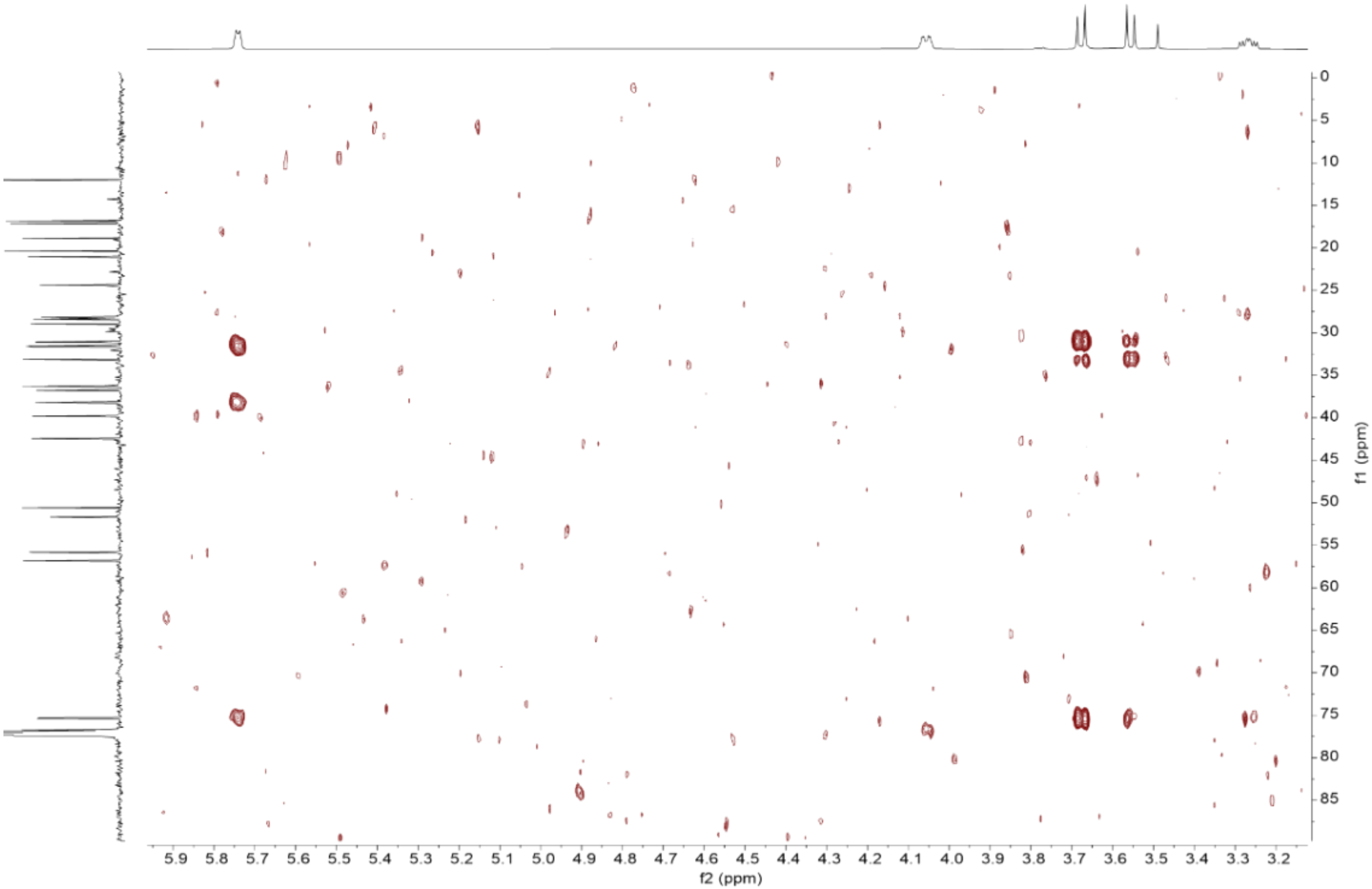

Figure S42. HMBC spectrum of **3** in $CDCl_3$ (expanded - 1)

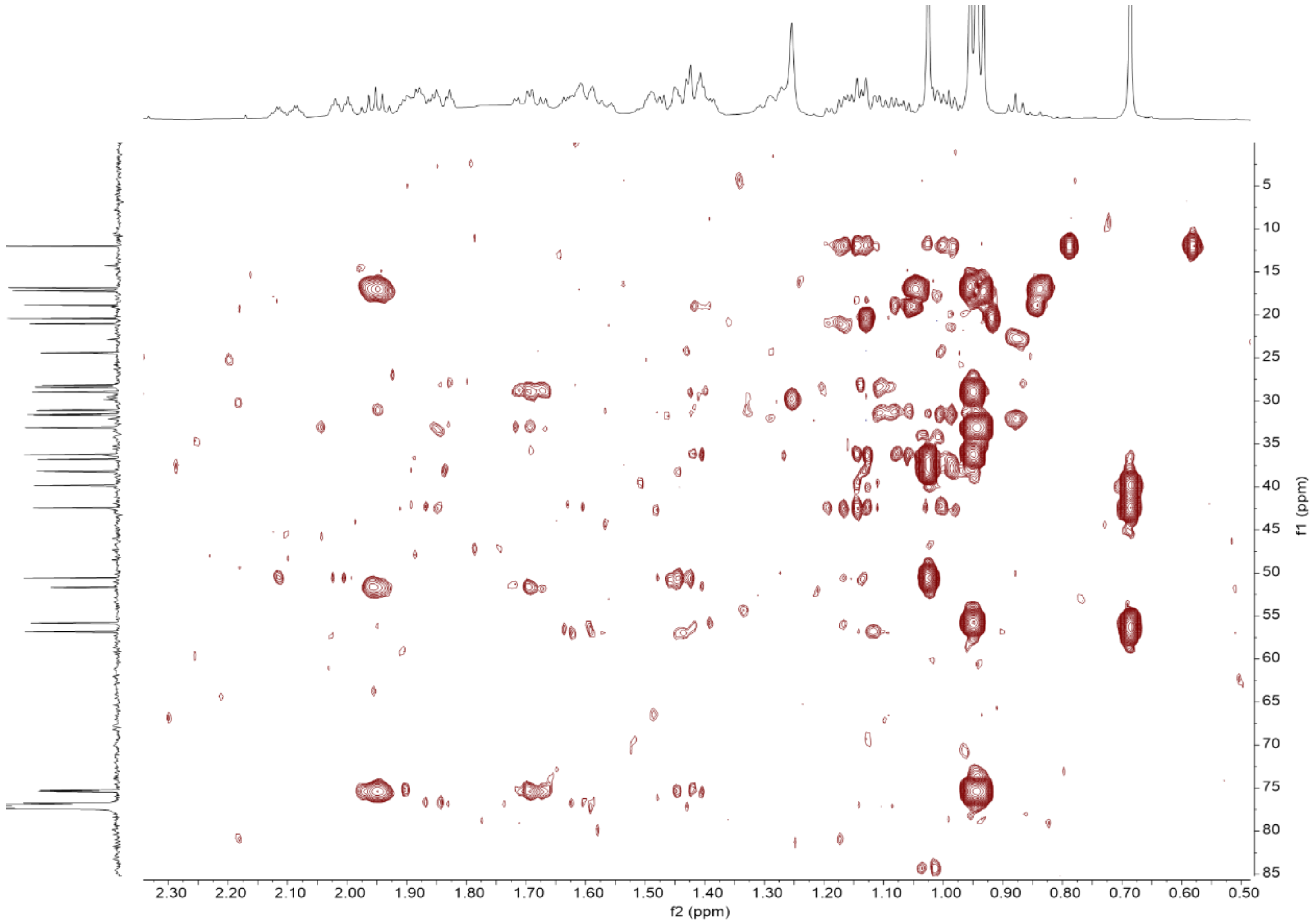

Figure S43. HMBC spectrum of **3** in $CDCl_3$ (expanded- 2)

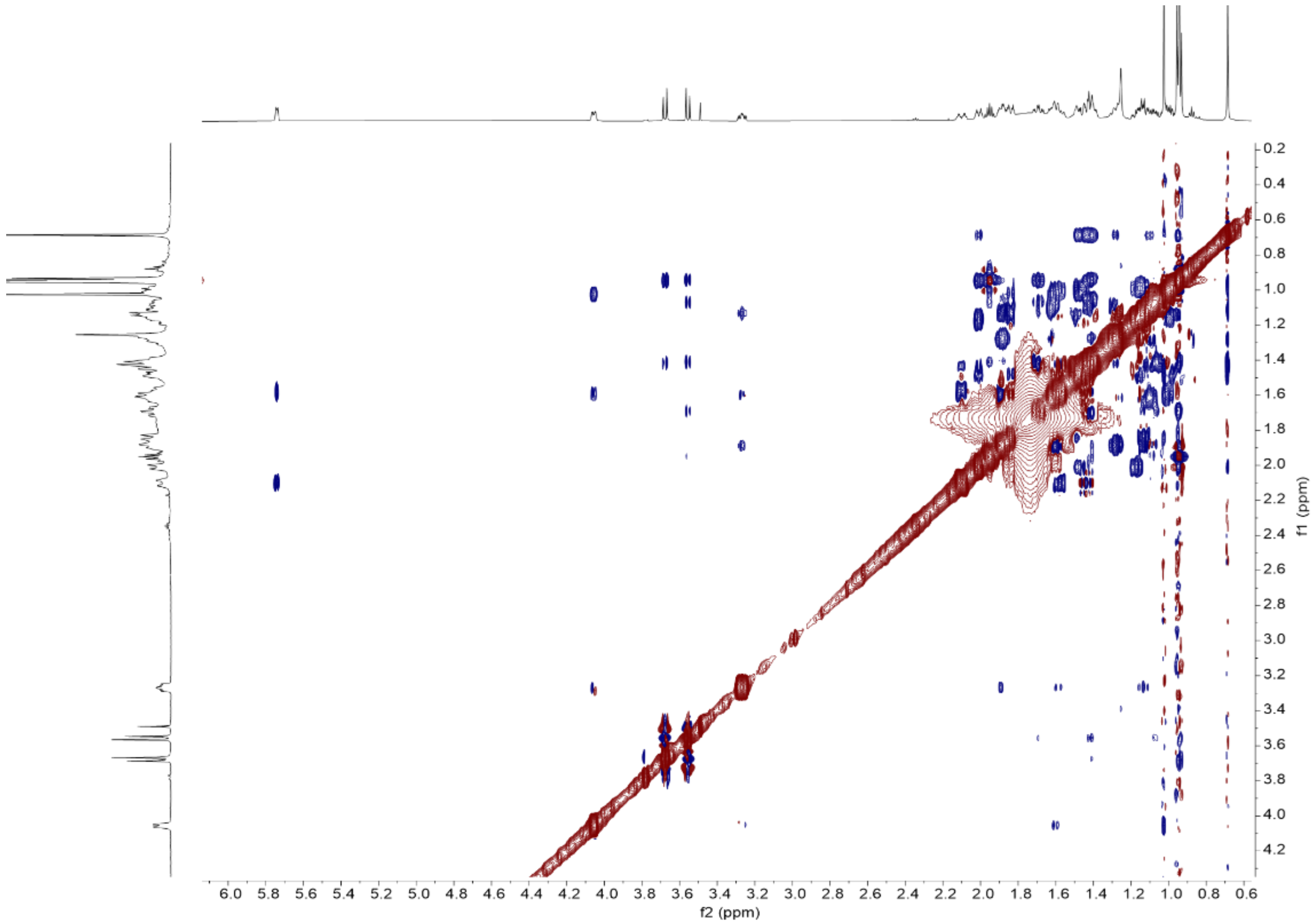

Figure S44. NOESY spectrum of **3** in $CDCl_3$

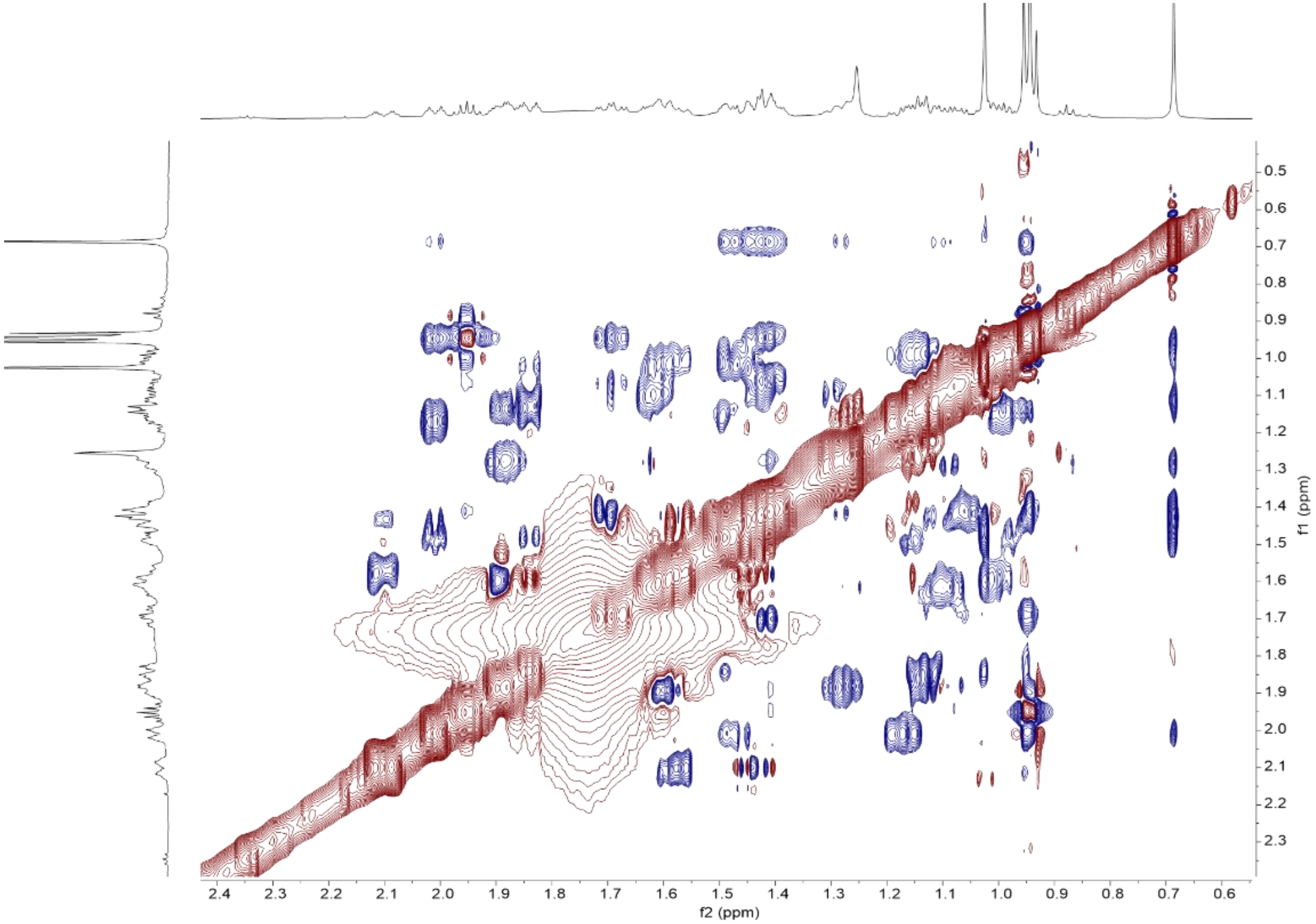

Figure S45. NOESY spectrum of **3** in $CDCl_3$ (expanded)

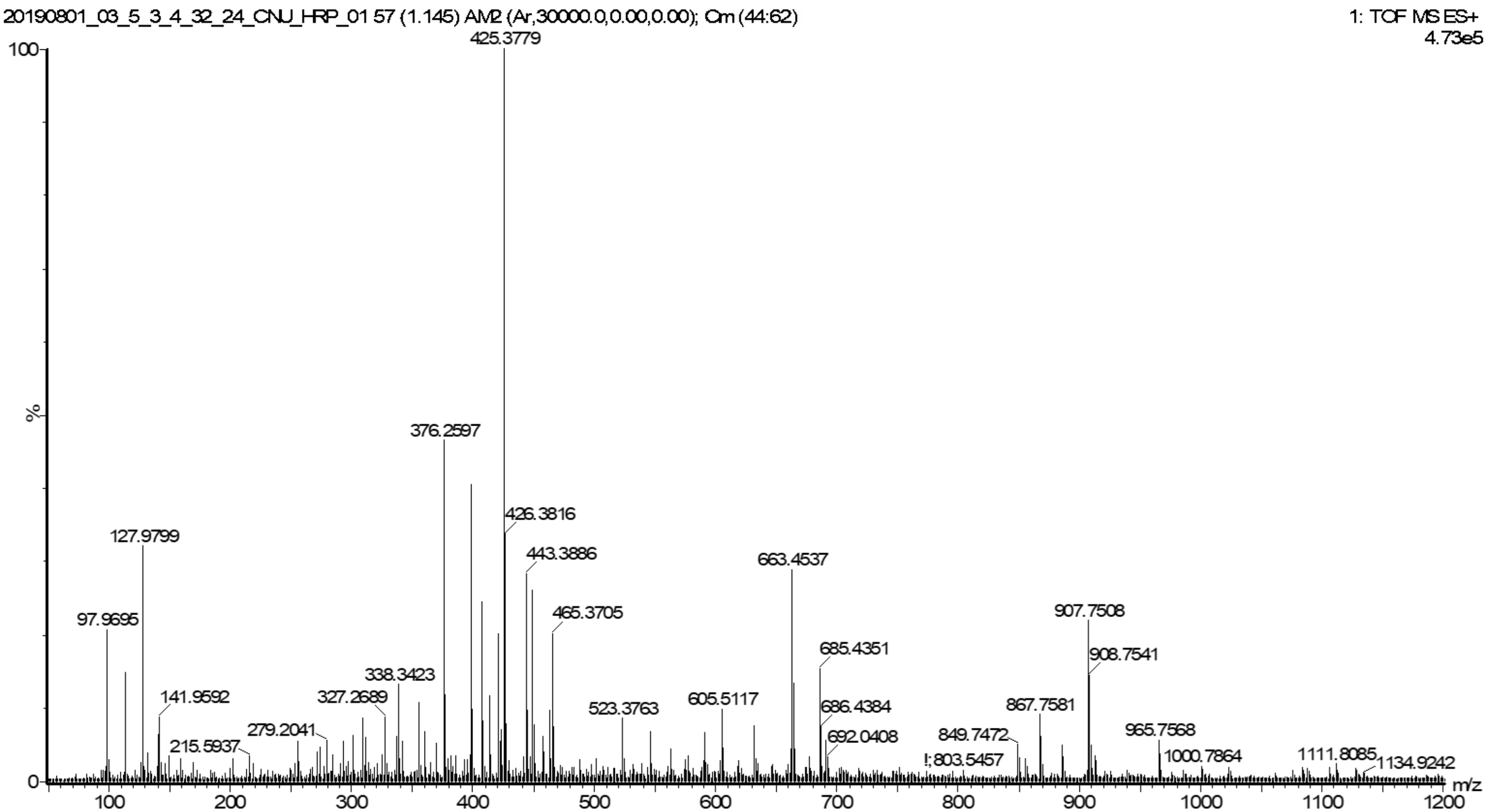

Figure S46. HRESIMS of **4**

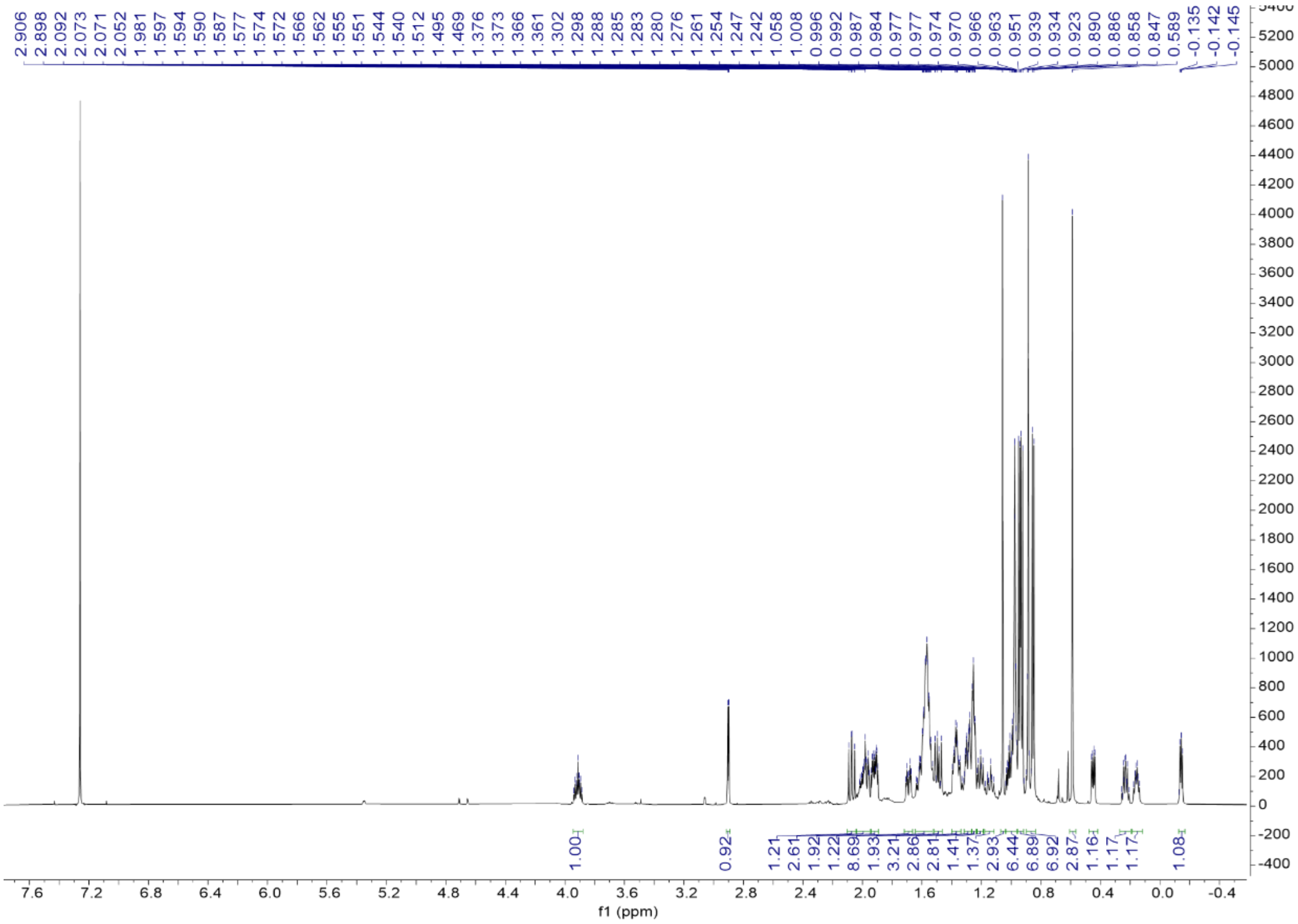

Figure S47. ^{1}H NMR spectrum of **4** in $CDCl_3$

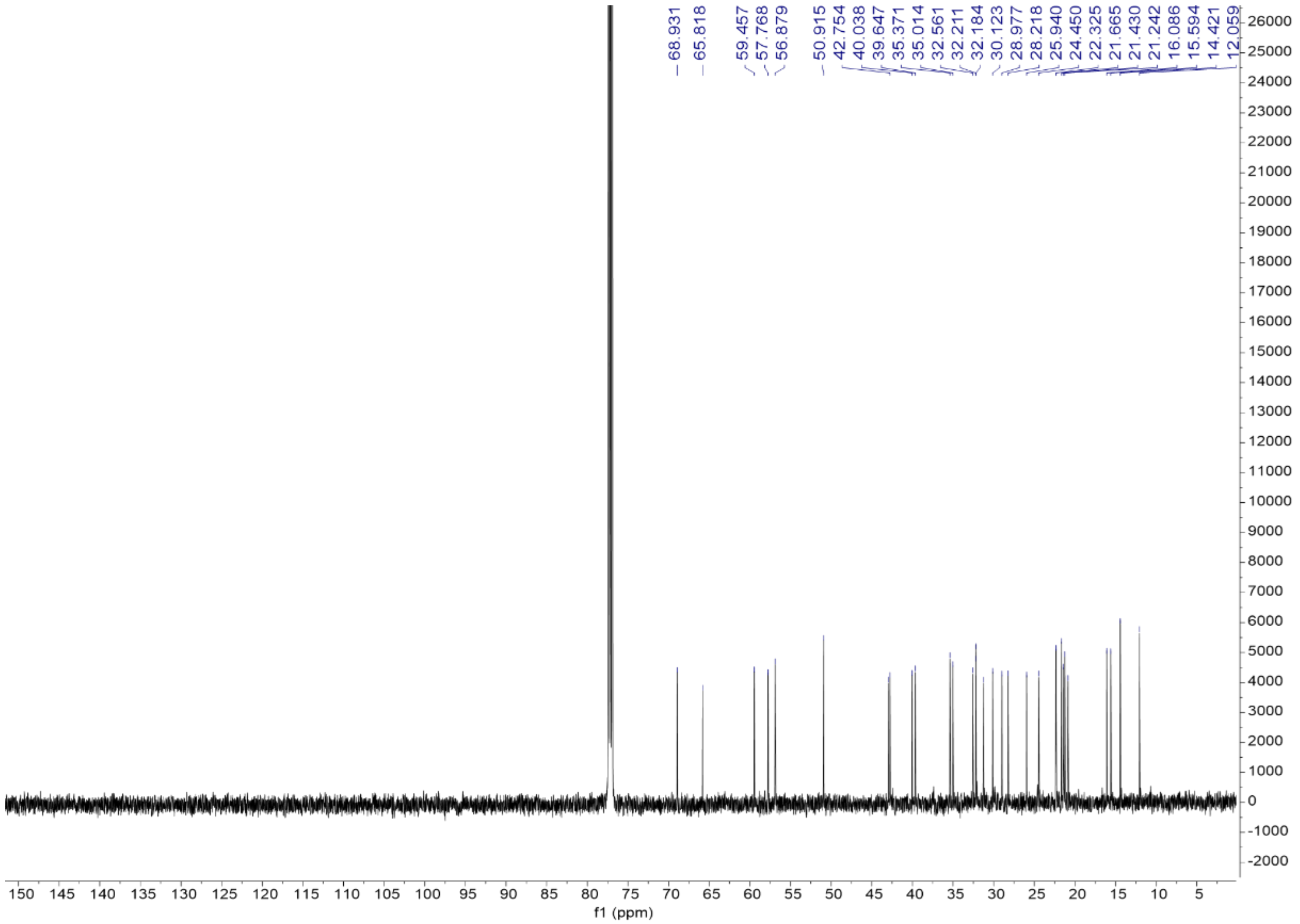

Figure S48. ¹³C NMR spectrum of **4** in $CDCl_3$

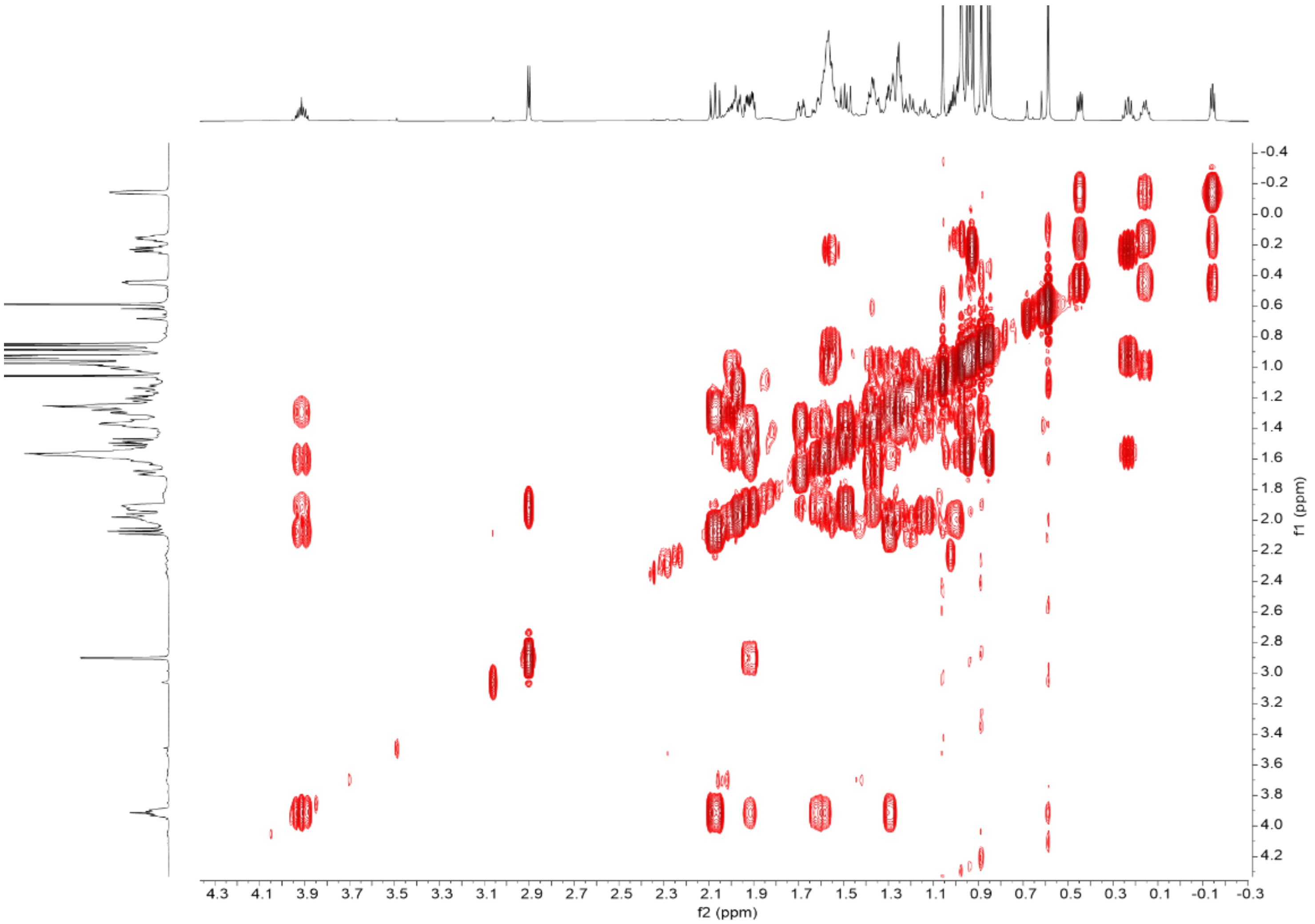

Figure S49. COSY spectrum of **4** in $CDCl_3$

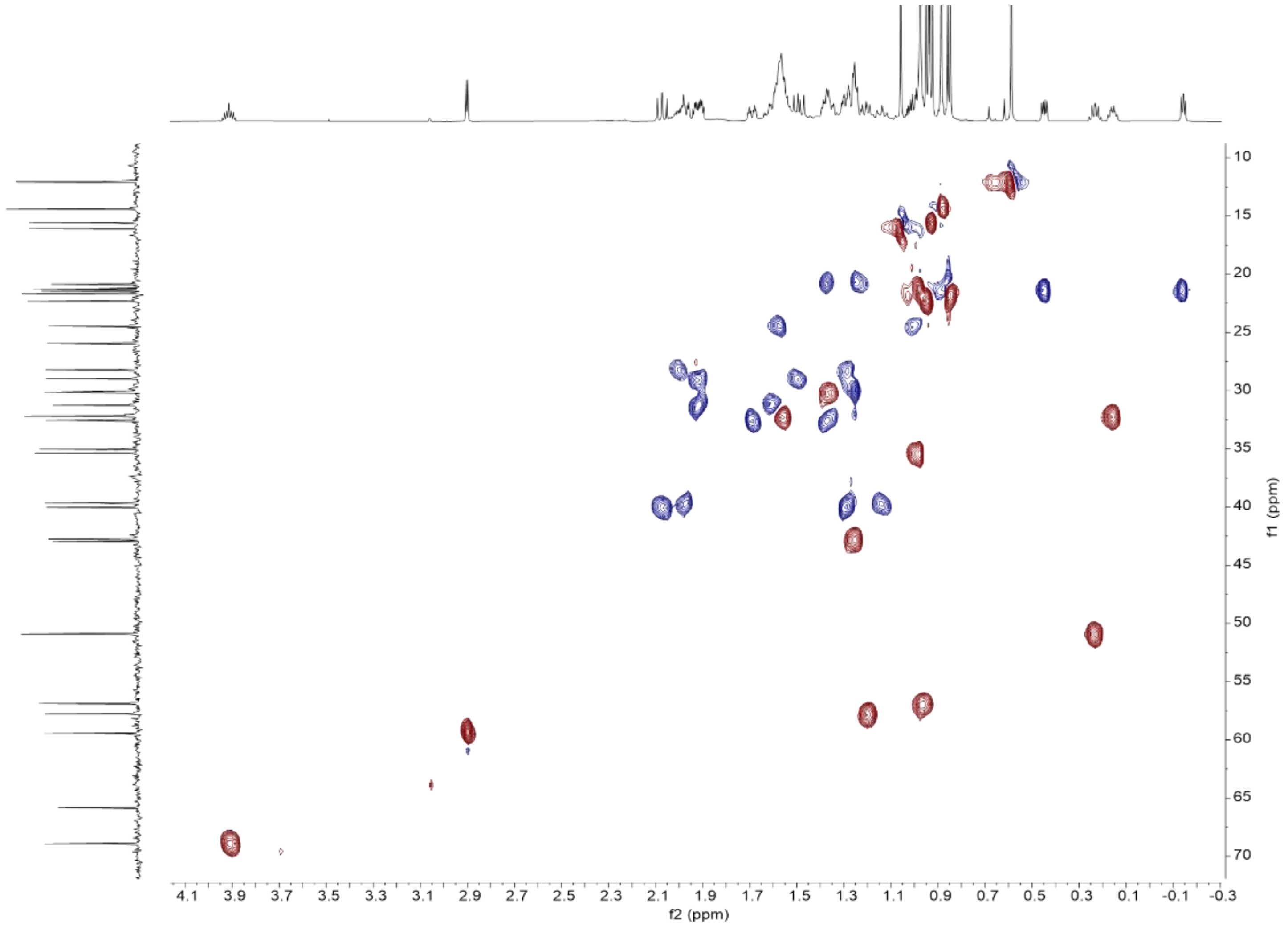

Figure S50. HSQC spectrum of **4** in $CDCl_3$

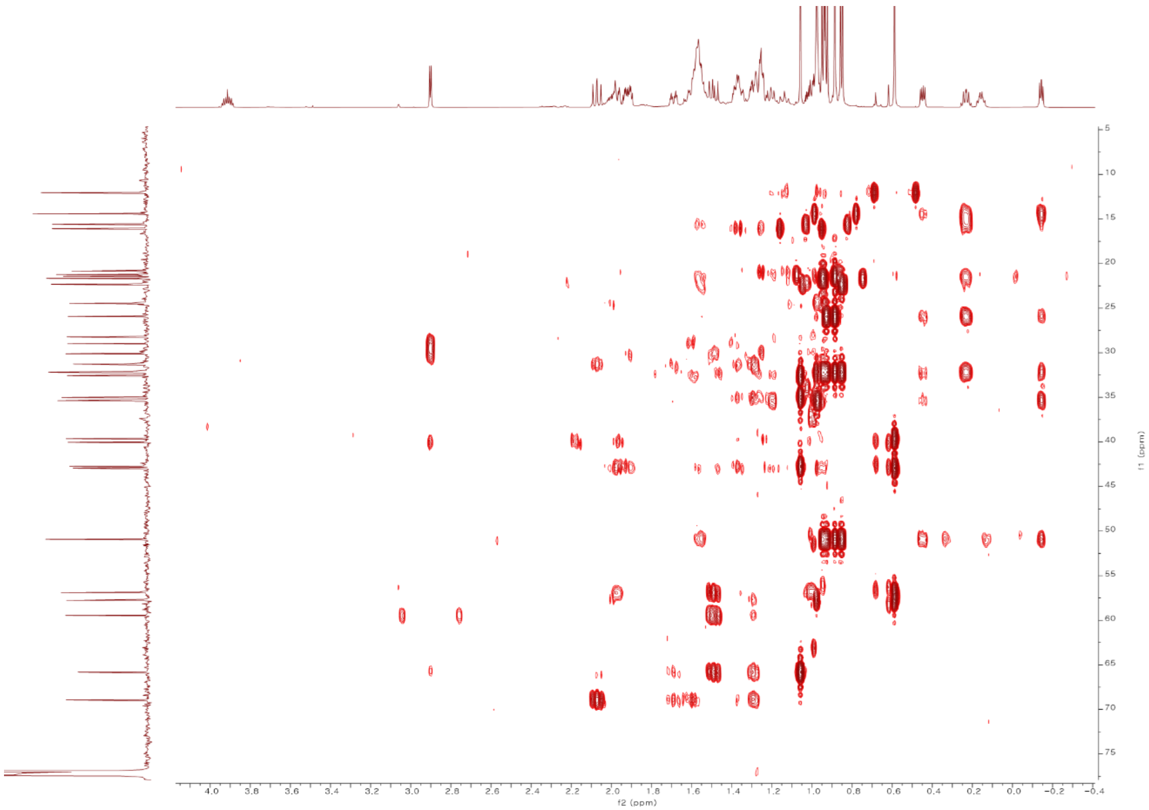

Figure S51. HMBC spectrum of **4** in $CDCl_3$

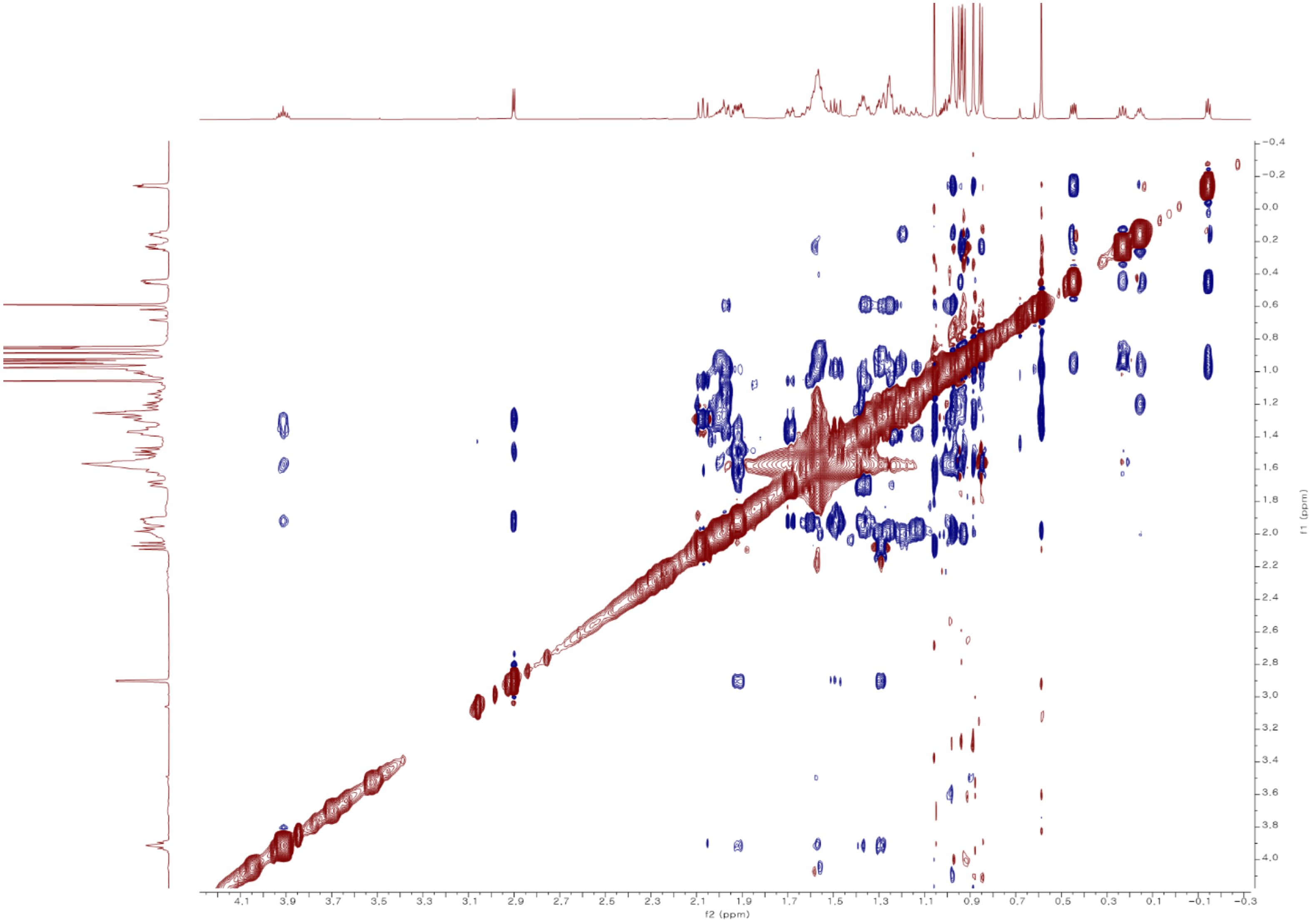

Figure S52. NOESY spectrum of **4** in $CDCl_3$

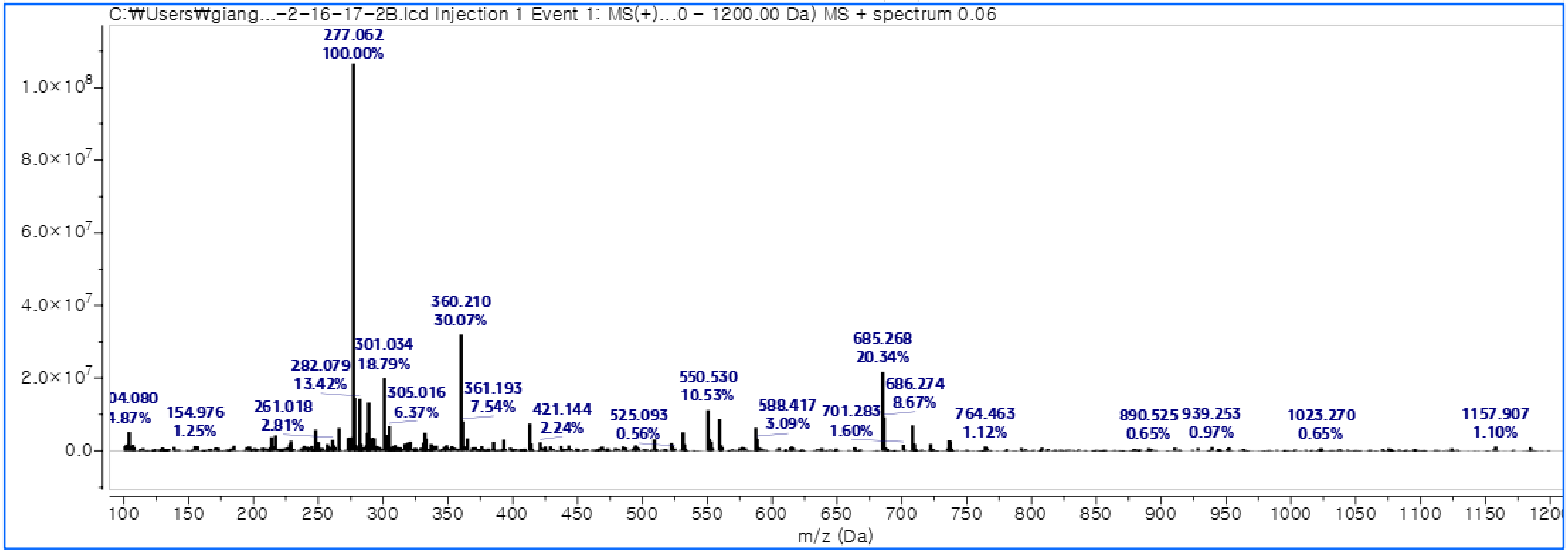

Figure S53. ESI-MS of **5**

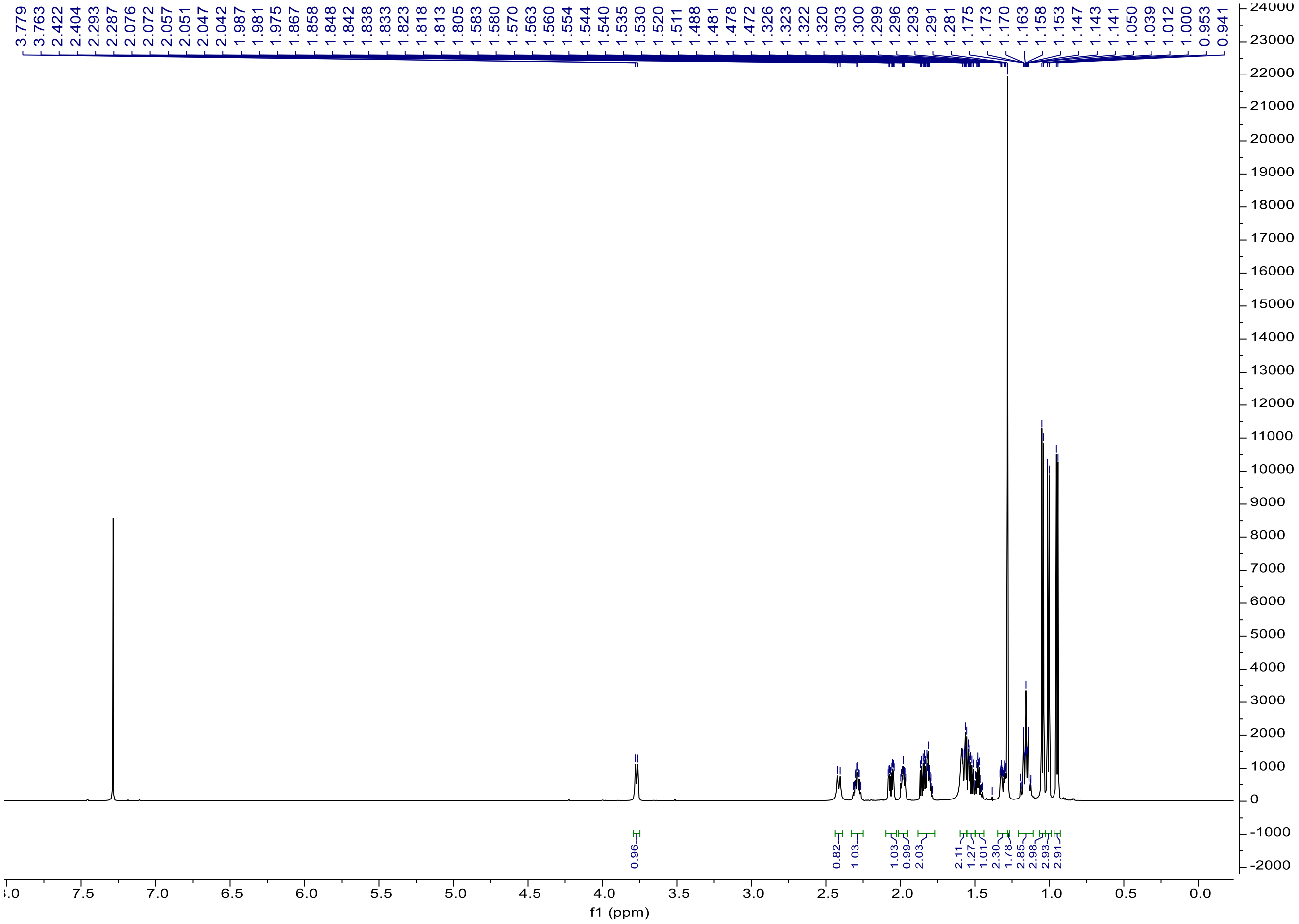

Figure S54. ^{1}H NMR spectrum of **5** in $CDCl_3$

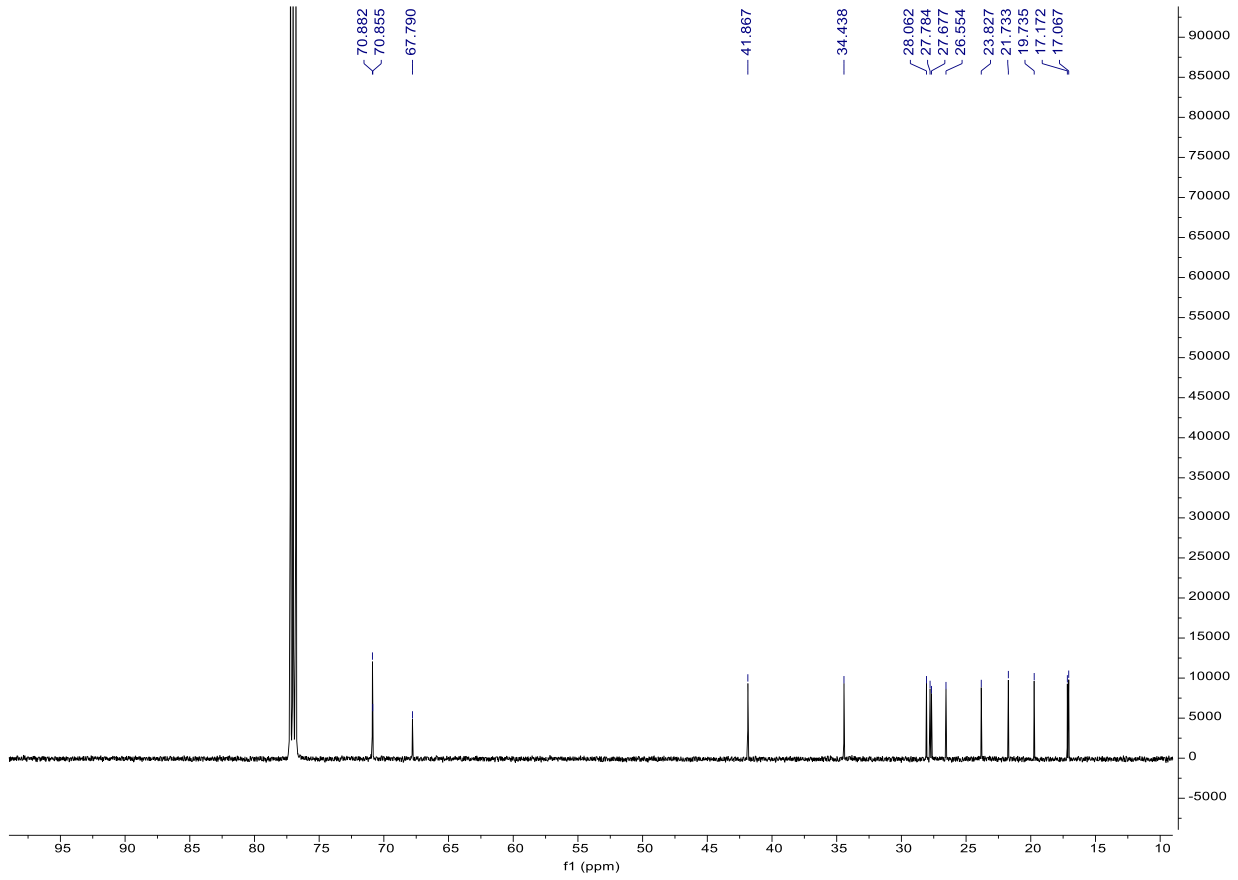

Figure S55. ^{13}C NMR spectrum of **5** in $CDCl_3$

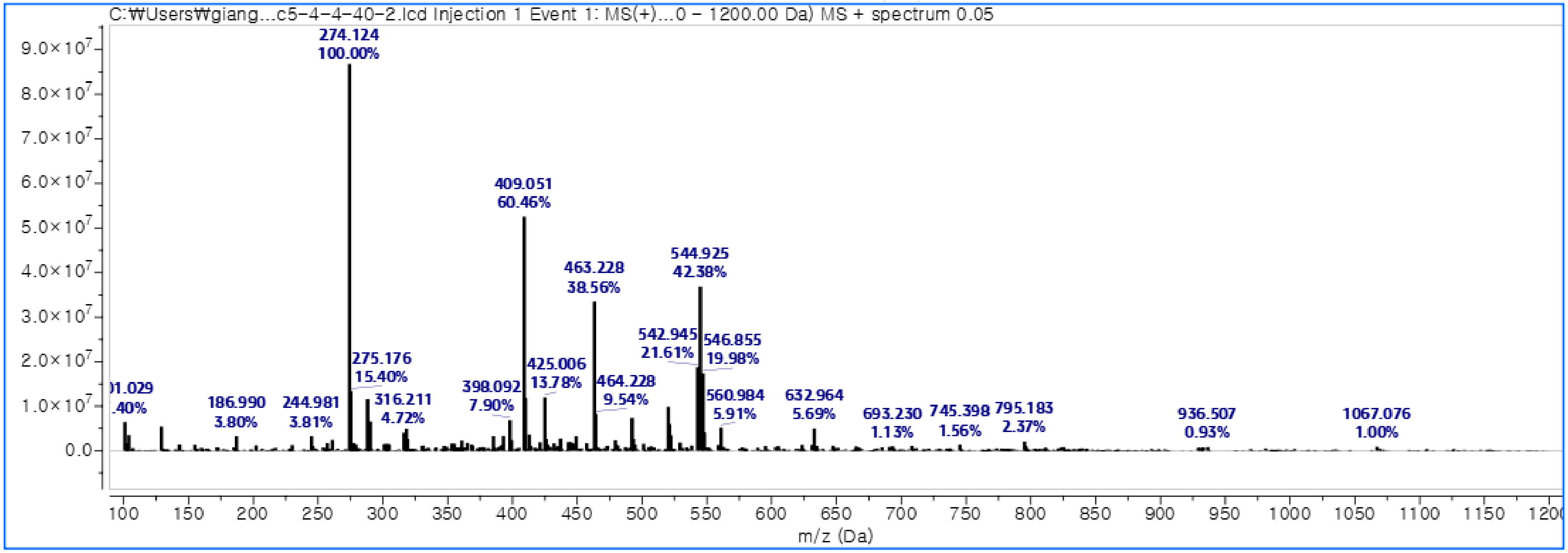

Figure S56. ESI-MS of **6**

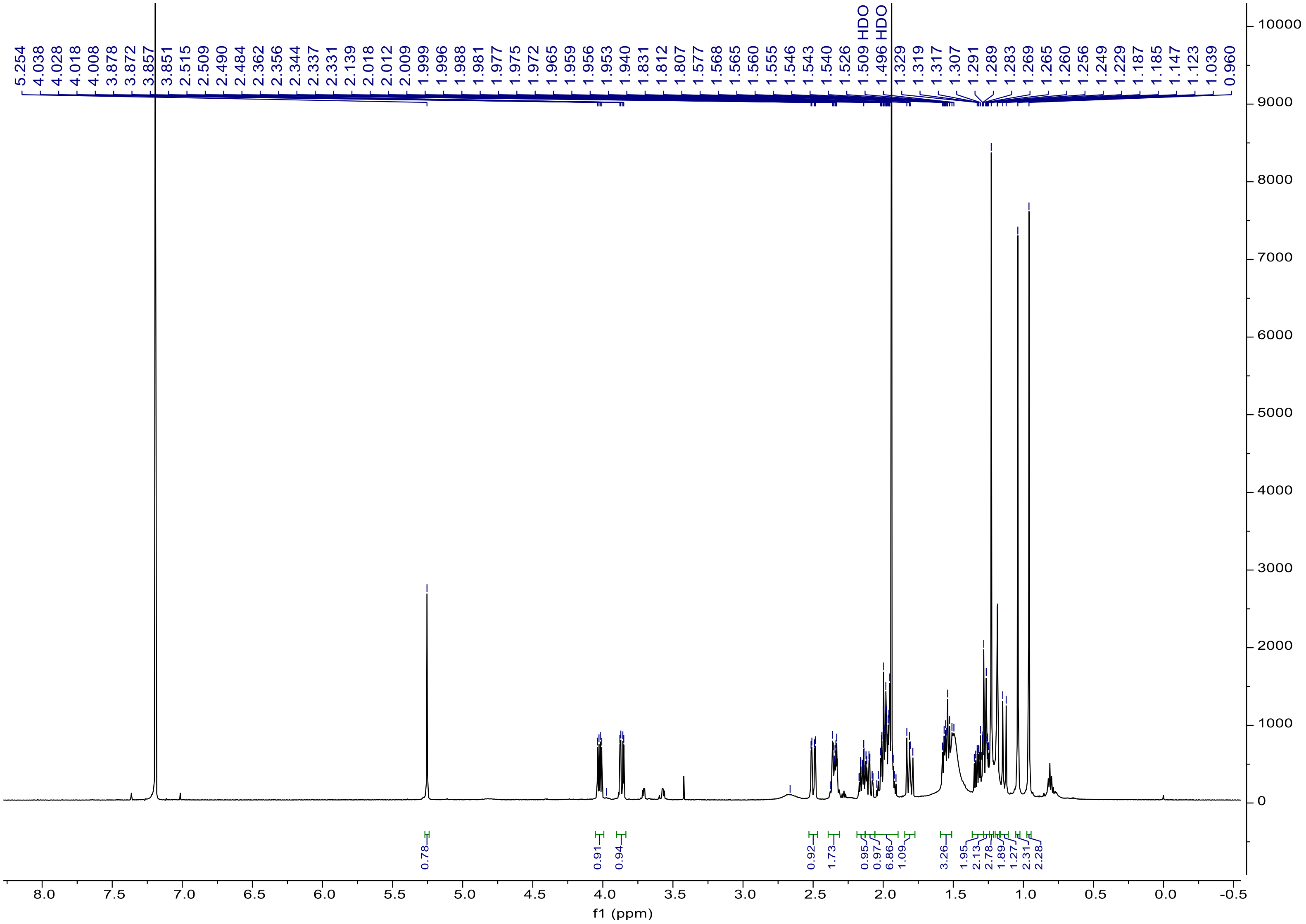

Figure S57. ^1H NMR spectrum of **6** in $CDCl_3$

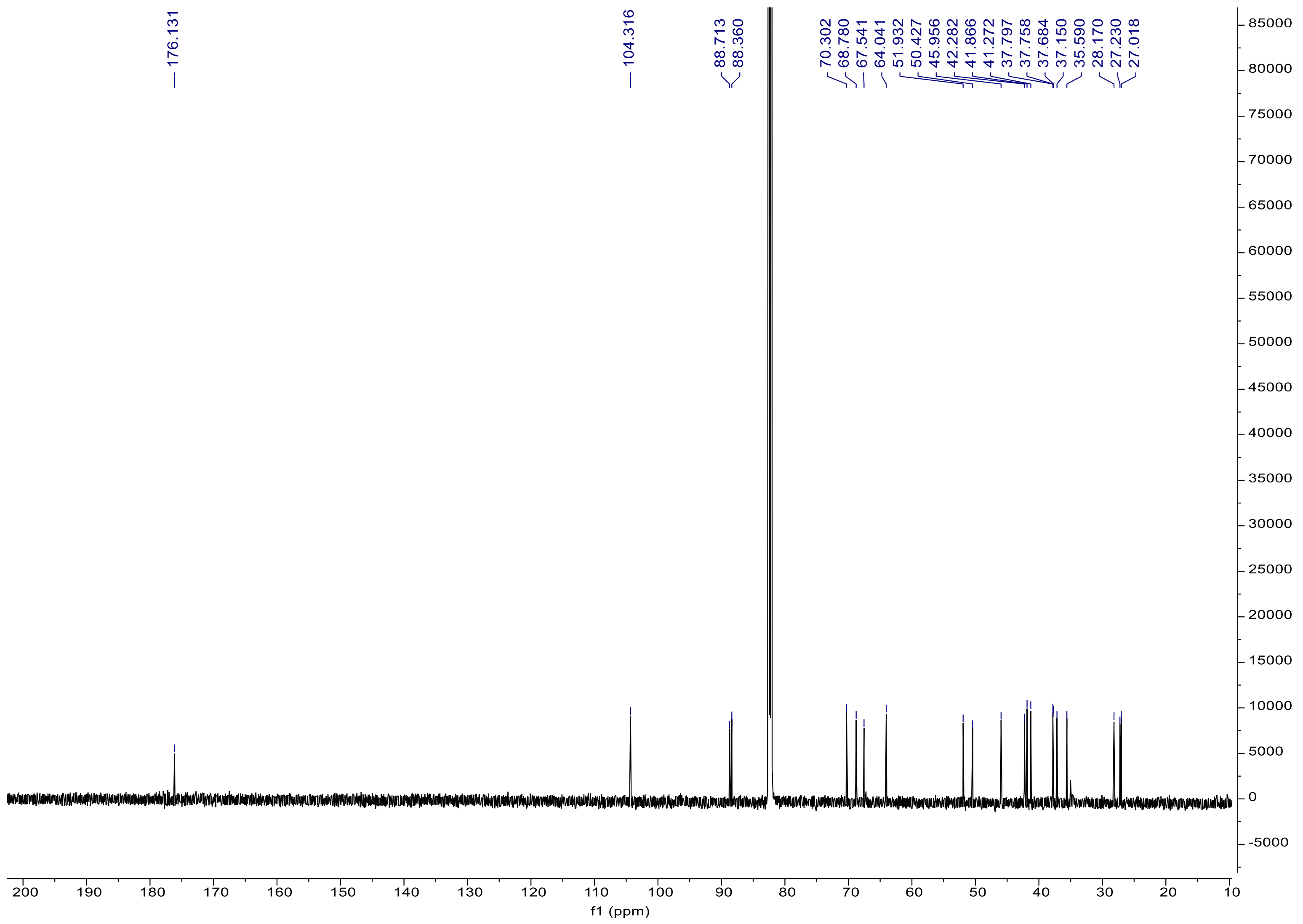

Figure S58. ^{13}C NMR spectrum of **6** in $CDCl_3$

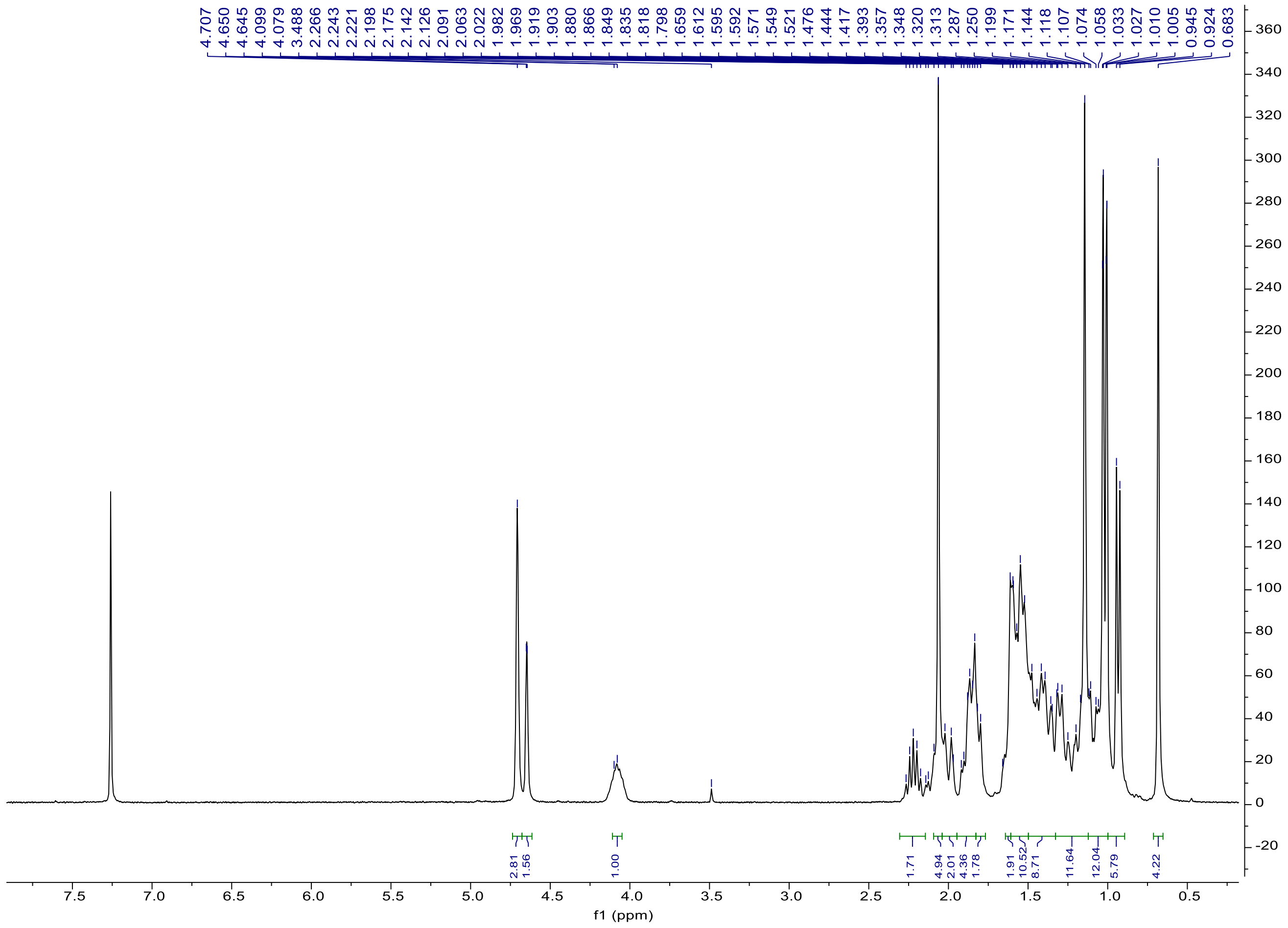

Figure S59. ^{1}H NMR spectrum of **7** in $CDCl_3$

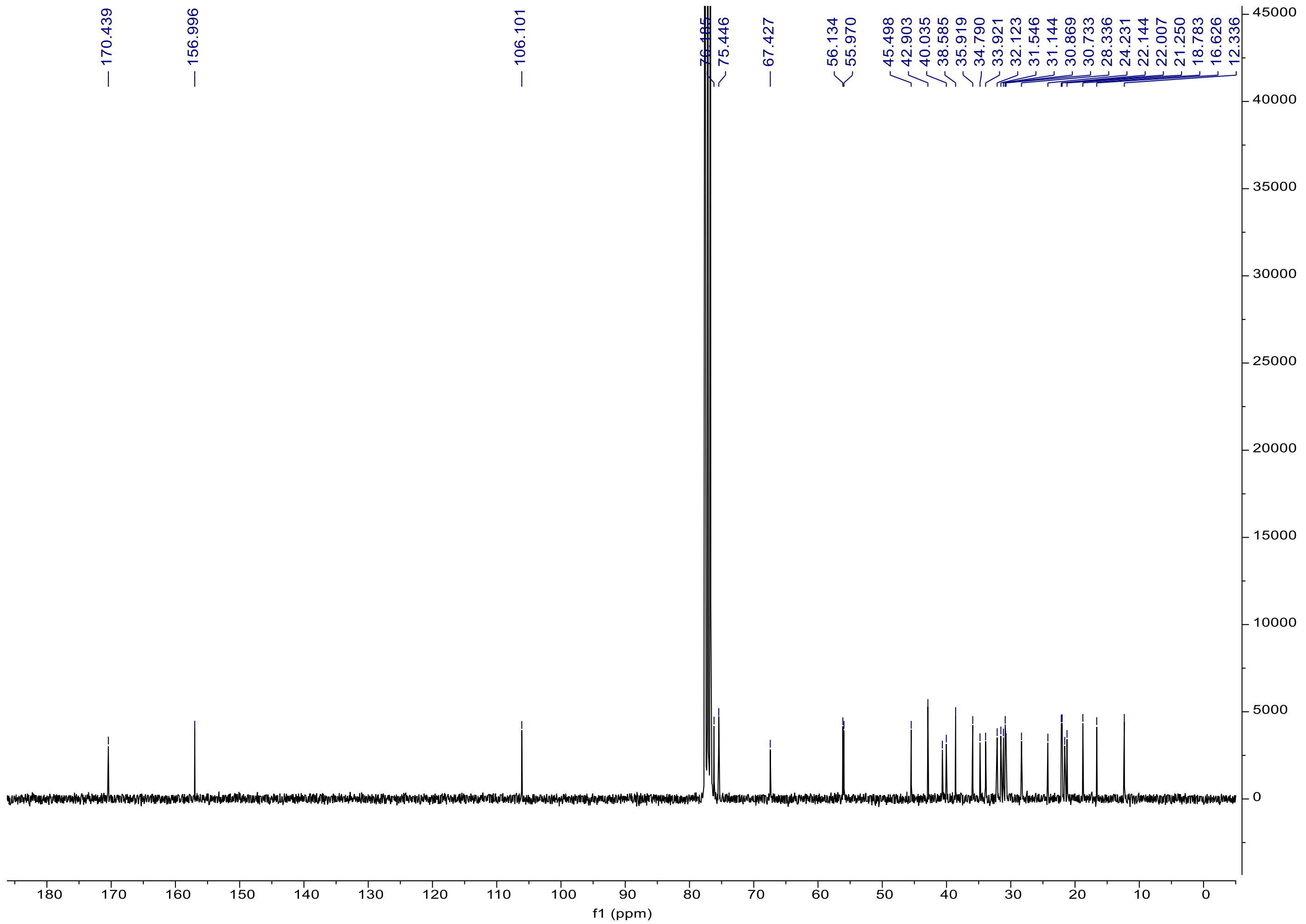

Figure S60. ^{13}C NMR spectrum of **7** in $CDCl_3$

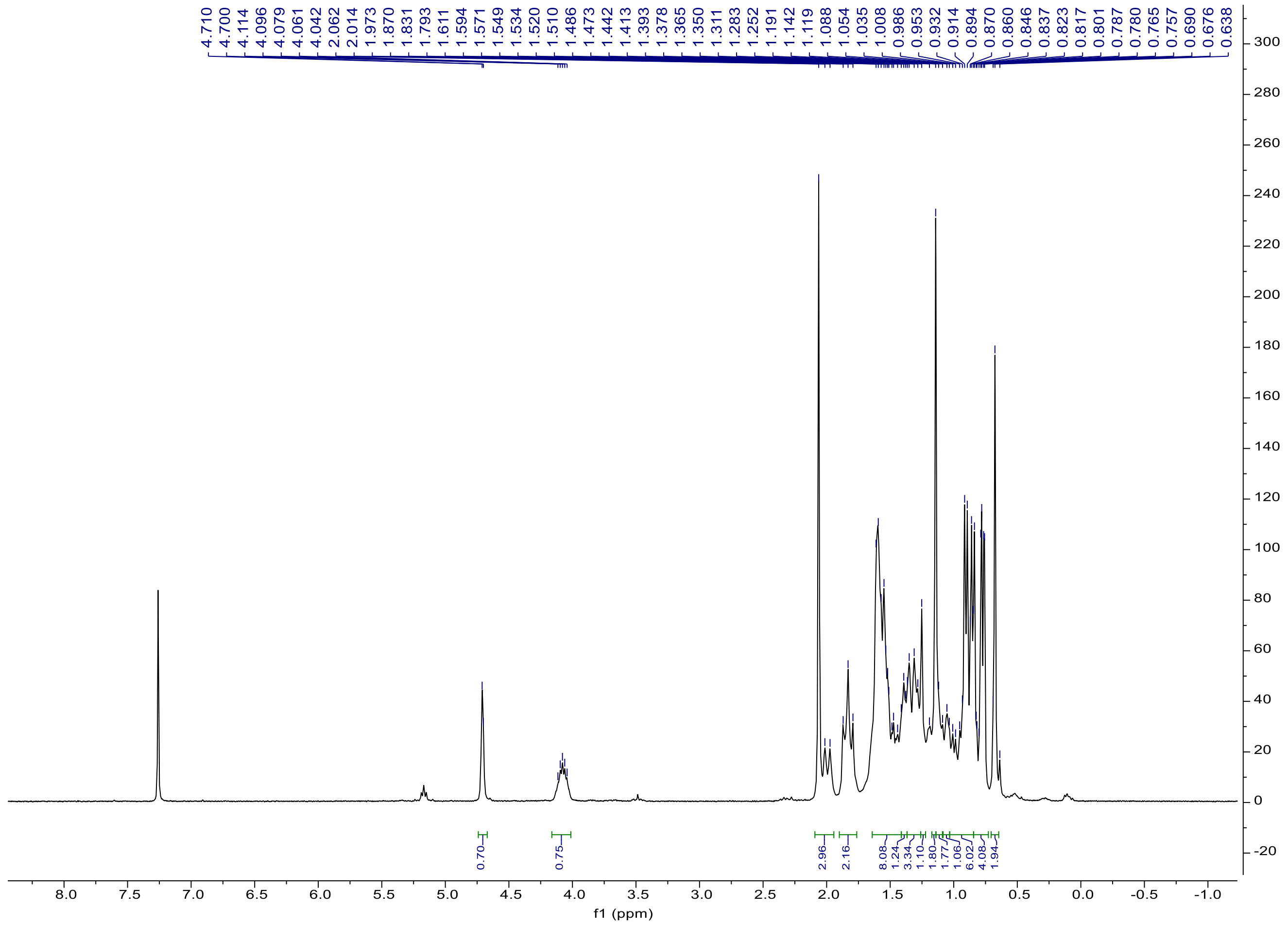

Figure S61. ^{1}H NMR spectrum of **8** in $CDCl_3$

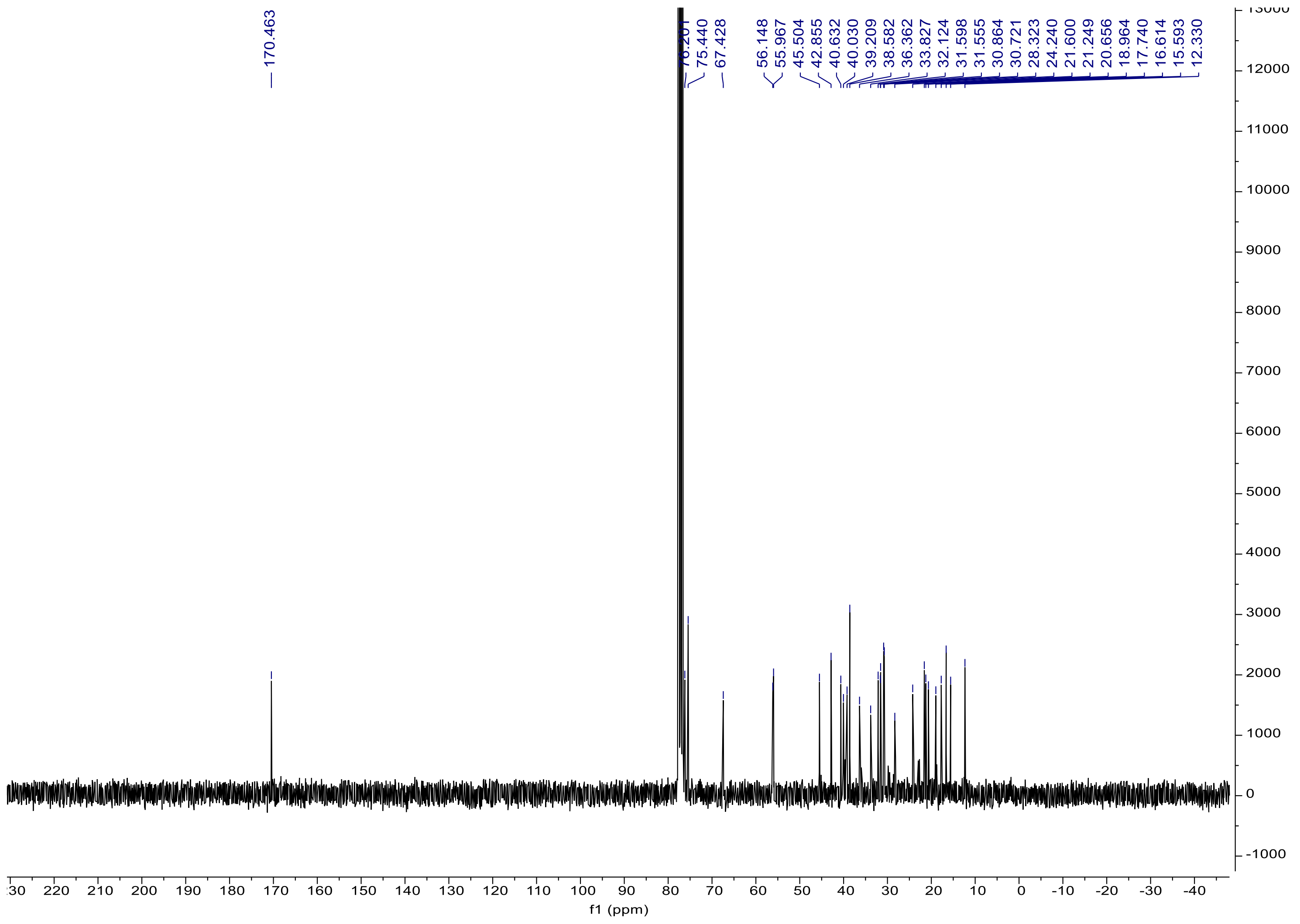

Figure S62. ^{13}C NMR spectrum of **8** in $CDCl_3$

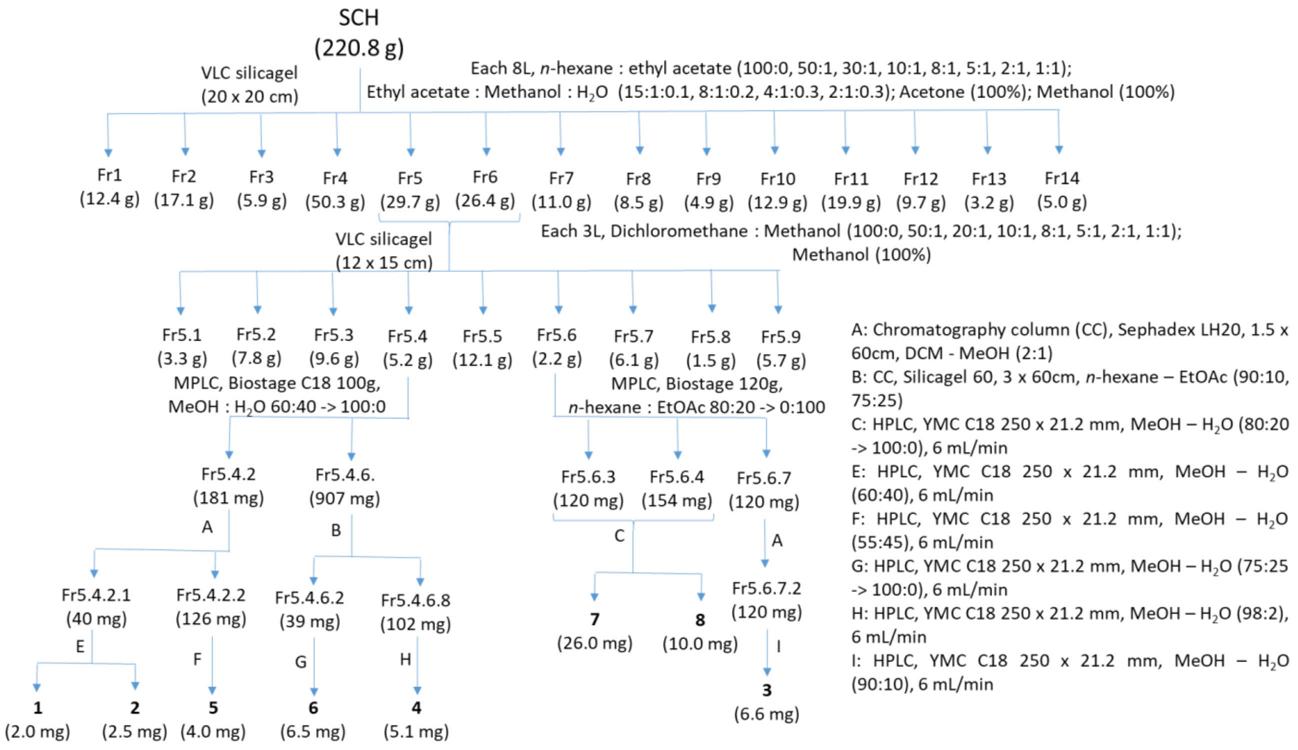

Figure S63. Isolation scheme of **1 – 8**

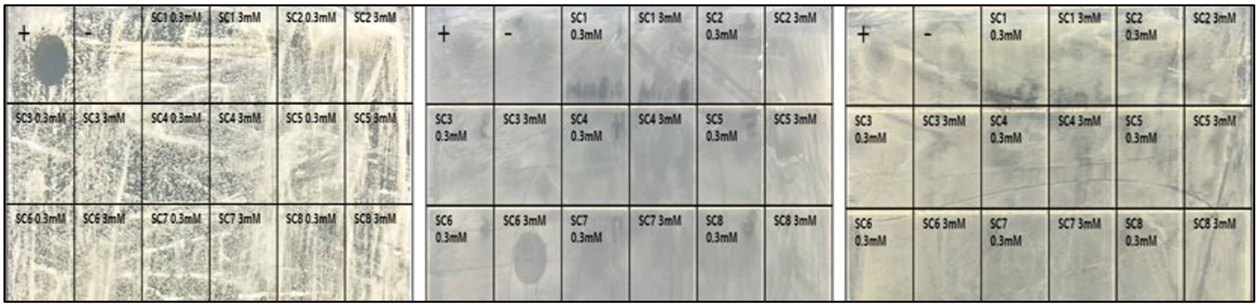

C. albicans
+: Nystatin 0.05 mM, 0.5 μg
-: 50% DMSO

S. aureus
+: Kanamycin 0.51 mM, 3 μg
-: 50% DMSO

E. coli
+: Kanamycin 0.51 mM, 3 μg
-: 50% DMSO

Figure S64. Antimicrobial activity of **1 – 8**

Table S1. Gibbs Free Energy and Boltzmann Population of **1a** (1*R*, 2*S*, 6*R*, 7*R*, 10*R*) and **1b** (1*S*, 2*R*, 6*S*, 7*S*, 10*S*) for ECD computation

Conformer	Gibbs Free Energy (Hartree)	Boltzmann Population (%)
1ac3	-812.608773	38.83
1ac1	-812.608670	34.82
1ac2	-812.607879	15.06
1ac5	-812.607050	6.26
1ac4	-812.606843	5.03
1bc3	-812.608773	38.83
1bc1	-812.608670	34.82
1bc2	-812.607879	15.06
1bc5	-812.607050	6.26
1bc4	-812.606843	5.03

Table S2. Gibbs Free Energy and Boltzmann Population of **2a** (1*R*, 2*S*, 6*S*, 7*R*, 10*S*) and **2b** (1*S*, 2*R*, 6*R*, 7*S*, 10*R*) for ECD computation

Conformer	Gibbs Free Energy (Hartree)	Boltzmann Population (%)
2ac1	-812.613471	46.41
2ac3	-812.613091	31.03
2ac2	-812.612714	20.82
2ac4	-812.610039	1.22
2ac5	-812.609219	0.51
2bc1	-812.613471	46.41
2bc3	-812.613091	31.03
2bc2	-812.612714	20.82
2bc4	-812.610039	1.22
2bc5	-812.609219	0.51

Table S3. Gibbs Free Energy and Boltzmann Population of 24*S* isomer of **3** for NMR computation

Conformer	Gibbs Free Energy (Hartree)	Boltzmann Population (%)
3ac6	-1780.569451	39.64
3ac3	-1780.568842	20.80
3ac5	-1780.568122	9.70
3ac15	-1780.568011	8.63
3ac4	-1780.567883	7.53
3ac1	-1780.567422	4.62
3ac2	-1780.566631	2.00
3ac12	-1780.566557	1.85
3ac9	-1780.566516	1.77
3ac17	-1780.565881	0.90
3ac10	-1780.56577	0.80
3ac11	-1780.565443	0.57
3ac7	-1780.565428	0.56
3ac16	-1780.564737	0.27
3ac8	-1780.564565	0.22
3ac13	-1780.563256	0.06
3ac14	-1780.563225	0.05
3ac18	-1780.562573	0.03

Table S4. Gibbs Free Energy and Boltzmann Population of 24*R* isomer of **3** for NMR computation

Conformer	Gibbs Free Energy (Hartree)	Boltzmann Population (%)
3bc10	-1780.569204	27.46
3bc6	-1780.568771	17.36
3bc12	-1780.568587	14.29
3bc8	-1780.568109	8.61
3bc9	-1780.567985	7.55
3bc16	-1780.567874	6.71
3bc7	-1780.567576	4.90
3bc11	-1780.567352	3.86
3bc2	-1780.567044	2.79
3bc5	-1780.566549	1.65
3bc4	-1780.566374	1.37
3bc13	-1780.56625	1.20
3bc1	-1780.566182	1.12
3bc3	-1780.565383	0.48
3bc14	-1780.565293	0.44
3bc15	-1780.564574	0.20

Table S5. Experimental and calculated NMR chemical shift values (ppm) of compound **3** with diastereomers 24*S* and 24*R*

(a) Original excel file for the experimental and calculated NMR chemical shift values (ppm) of compound **3**

DP4+ analysis.xlsx - Excel

Functional	Solvent?	Basis Set	Type of Data
mPW1PW91	PCM	6-311+G(d,p)	Unscaled Shifts

Nuclei	sp2?	Experimental	Isomer 1	Isomer 2	Isomer 3	Isomer 4	Isomer 5	Isomer 6	Isomer 7	Isomer 8	Isomer 9	Isomer 10	Isomer 11
		DP4+	0.00%	100.00%	-	-	-	-	-	-	-	-	-
C		36.78	40.12	40.00									
C		28.18	31.21	31.22									
C		76.75	81.05	80.96									
C		75.29	80.35	80.37									
C	x	142.16	153.02	153.13									
C	x	117.94	127.02	126.95									
C		31.56	35.28	35.30									
C		31.66	35.48	35.44									
C		50.59	54.38	54.40									
C		38.18	44.54	44.52									
C		21.04	24.60	24.61									
C		39.82	43.30	43.28									
C		42.44	48.35	48.38									
C		56.83	60.15	60.13									
C		24.43	28.06	28.07									
C		28.39	32.41	32.50									
C		55.82	60.52	60.58									
C		12	12.94	12.93									
C		20.39	22.08	22.06									
C		36.26	42.69	42.34									
C		18.89	19.16	19.16									
C		28.97	30.88	31.22									
C		31.09	31.12	36.00									
C		75.4	80.58	79.89									
C		33.12	39.41	37.35									
C		16.85	16.95	17.67									
C		17.18	18.52	17.94									
C		51.68	64.15	65.18									
H		1.13	1.19	1.18									
H		1.84	1.88	1.88									
H		1.6	1.62	1.62									
H		1.9	1.83	1.83									
H		3.27	3.20	3.21									
H		4.06	4.09	4.08									
H	x	5.74	6.09	6.10									

Main | Detailed Results

Functional	Solvent?	Basis Set	Type of Data
mPW1PW91	PCM	6-311+G(d,p)	Unscaled Shifts

	Isomer 1	Isomer 2	Isomer 3	Isomer 4	Isomer 5	Isomer 6	Isomer 7	Isomer 8	Isomer 9	Isomer 10	Isomer 11	Isomer 12
sDP4+ (H data)	0.25%	99.75%	-	-	-	-	-	-	-	-	-	-
sDP4+ (C data)	12.83%	87.17%	-	-	-	-	-	-	-	-	-	-
sDP4+ (all data)	0.04%	99.96%	-	-	-	-	-	-	-	-	-	-
uDP4+ (H data)	0.39%	99.61%	-	-	-	-	-	-	-	-	-	-
uDP4+ (C data)	2.53%	97.47%	-	-	-	-	-	-	-	-	-	-
uDP4+ (all data)	0.01%	99.99%	-	-	-	-	-	-	-	-	-	-
DP4+ (H data)	0.00%	100.00%	-	-	-	-	-	-	-	-	-	-
DP4+ (C data)	0.38%	99.62%	-	-	-	-	-	-	-	-	-	-
DP4+ (all data)	0.00%	100.00%	-	-	-	-	-	-	-	-	-	-

(b) Reorganized table for the experimental and calculated NMR chemical shift values (ppm) of compound **3**

Number	Experimental	Diastereomer 24*S*	Diastereomer 24*R*
C-1	36.78	40.12	40.00
C-2	28.18	31.21	31.22
C-3	76.75	81.05	80.96
C-4	75.29	80.35	80.37
C-5	142.16	153.02	153.13
C-6	117.94	127.02	126.95
C-7	31.56	35.28	35.30
C-8	31.66	35.48	35.44
C-9	50.59	54.38	54.40
C-10	38.18	44.54	44.52
C-11	21.04	24.60	24.61
C-12	39.82	43.30	43.28
C-13	42.44	48.35	48.38
C-14	56.83	60.15	60.13
C-15	24.43	28.06	28.07
C-16	28.39	32.41	32.50
C-17	55.82	60.52	60.58
C-18	12.00	12.94	12.93
C-19	20.39	22.08	22.06
C-20	36.26	42.69	42.34
C-21	18.89	19.16	19.16
C-22	28.97	30.88	31.22
C-23	31.09	31.12	36.00
C-24	75.40	80.58	79.89
C-25	33.12	39.41	37.35
C-26	16.85	16.95	17.67
C-27	17.18	18.52	17.94
C-28	51.68	64.15	65.18

H-1a	1.13	1.19	1.18
H-1b	1.84	1.88	1.88
H-2a	1.60	1.62	1.62
H-2b	1.90	1.83	1.83
H-3	3.27	3.20	3.21
H-4	4.06	4.09	4.08
H-6	5.74	6.09	6.10
H-7a	1.58	1.67	1.66
H-7b	2.10	2.14	2.13
H-8	1.44	1.56	1.56
H-9	0.99	1.01	1.00
H-11a	1.49	1.46	1.46
H-11b	1.49	1.54	1.54
H-12a	1.16	1.24	1.25
H-12b	2.02	2.04	2.05
H-14	1.00	1.10	1.09
H-15a	1.10	1.19	1.18
H-15b	1.62	1.60	1.59
H-16a	1.27	1.37	1.34
H-16b	1.89	1.90	1.92
H-17	1.14	1.15	1.15
H-18a	0.69	0.57	0.57
H-18b	0.69	0.67	0.66
H-18c	0.69	1.04	1.04
H-19a	1.02	0.88	0.87
H-19b	1.02	0.92	0.89
H-19c	1.02	1.35	1.36
H-20	1.40	1.35	1.33
H-21a	0.95	0.43	0.61
H-21b	0.95	0.96	1.00
H-21c	0.95	1.24	1.07

H-22a	1.07	0.78	0.81
H-22b	1.42	1.82	1.54
H-23a	1.41	1.35	1.40
H-23b	1.70	1.52	1.73
H-25	1.95	2.00	1.92
H-26a	0.93	0.82	0.80
H-26b	0.93	0.73	0.81
H-26c	0.93	1.45	1.48
H-27a	0.94	0.75	0.73
H-27b	0.94	0.93	0.96
H-27c	0.94	0.98	1.00
H-28a	3.56	3.94	3.78
H-28b	3.68	3.94	4.00
